# Fluorine‐Directed Automated Mannoside Assembly

**DOI:** 10.1002/anie.202213304

**Published:** 2022-12-12

**Authors:** Charlotte S. Teschers, Ryan Gilmour

**Affiliations:** ^1^ Organisch-Chemisches Institut Westfälische Wilhelms-Universität Münster Corrensstr. 36 48149 Münster Germany

**Keywords:** Automated Glycan Assembly, Carbohydrates, Fluorination, Oligomannose, Stereoselective Synthesis

## Abstract

Automated glycan assembly (AGA) on solid support has become invaluable in reconciling the biological importance of complex carbohydrates with the persistent challenges associated with reproducible synthesis. Whilst AGA platforms have transformed the construction of many natural sugars, validation in the construction of well‐defined (site‐selectively modified) glycomimetics is in its infancy. Motivated by the importance of fluorination in drug discovery, the biomedical prominence of 2‐fluoro sugars and the remarkable selectivities observed in fluorine‐directed glycosylation, fluorine‐directed automated glycan assembly (FDAGA) is disclosed. This strategy leverages the fluorine atom for stereocontrolled glycosylation on solid support, thereby eliminating the reliance on *O*‐based directing groups. The logical design of C2‐fluorinated mannose building blocks, and their application in the fully (α‐)stereocontrolled automated assembly of linear and branched fluorinated oligomannosides, is disclosed. This operationally simple strategy can be integrated into existing AGA and post‐AGA protocols to augment the scope of programmed carbohydrate synthesis.

## Graphical abstract

Motivated by the importance of fluorination in drug discovery, fluorine‐directed automated glycan assembly (AFGA) is disclosed. This strategy leverages the C(sp^3^)−F bond at C2 to enable α‐stereocontrolled glycosylation that eliminates the reliance on *O*‐based directing groups. Reactions display extremely high levels of efficiency and selectivity, and the method has been demonstrated in the synthesis of linear and branched fluorinated oligomannoses.

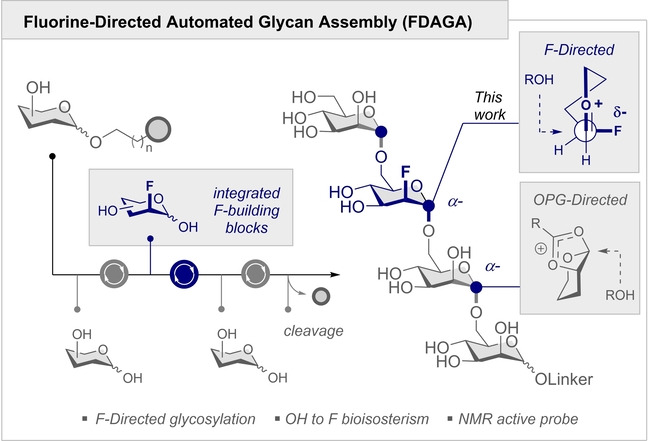

## Introduction

**Figure 1:**
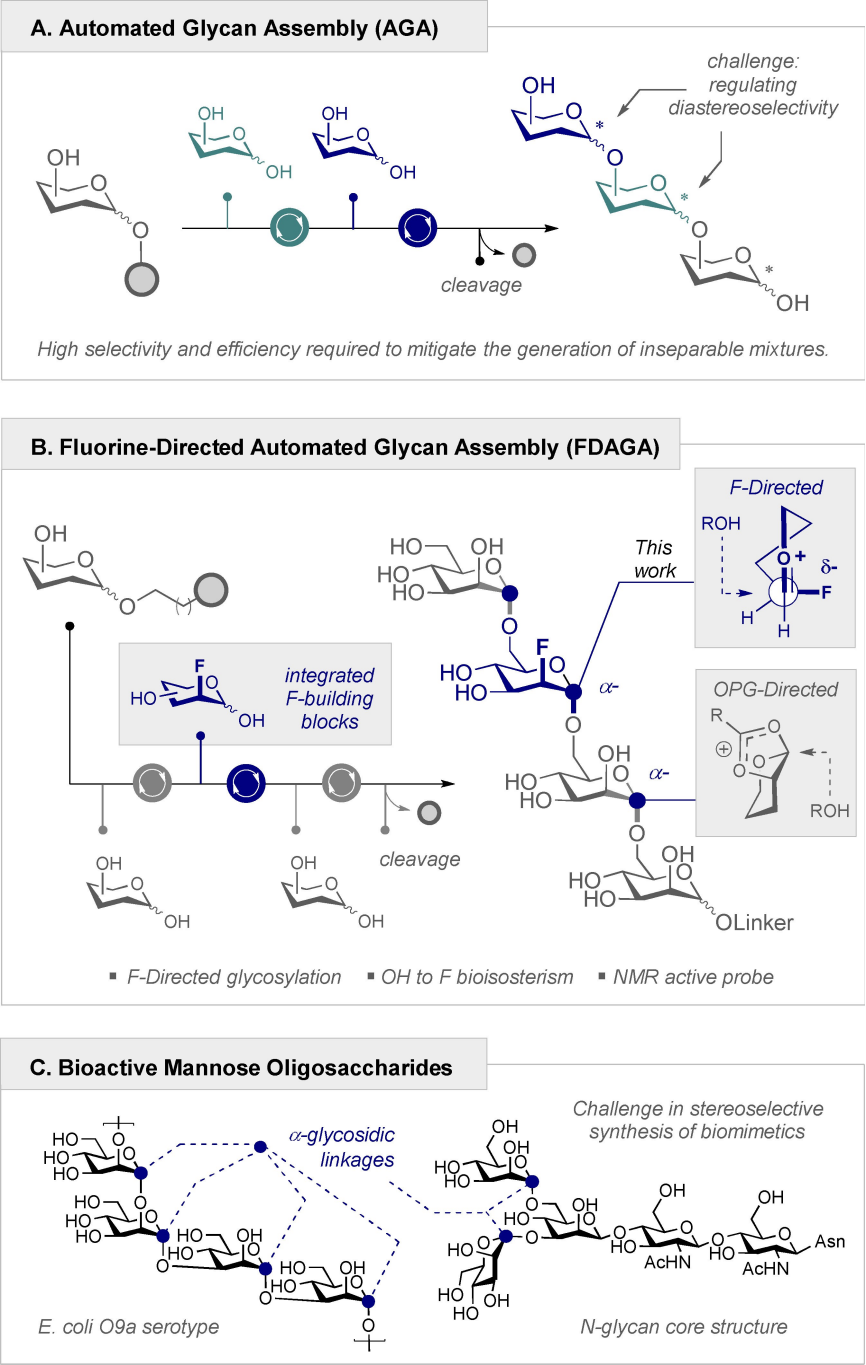
A) An overview of automated glycan assembly (AGA). B) Fluorine‐directed automated glycan assembly (FDAGA) and the role of F in orchestrating selectivity. C) Selected examples of bioactive mannose‐containing oligosaccharides containing α‐configured glycosidic linkages. PG=protecting group.

As the glycoscience revolution continues to build momentum, the ubiquity and diversity of carbohydrates in all biological kingdoms creates opportunities for creative endeavor in the stereocontrolled synthesis of complex glycans.^[1]^ The biological importance of oligosaccharides, combined with the conspicuous absence of biosynthesis blueprints that can be reproduced in the laboratory, provide compelling arguments for the development of enabling synthesis platforms. Emulating earlier triumphs in solid phase biopolymer synthesis, automated glycan assembly (AGA) has transformed the construction of many complex carbohydrates, providing a platform to engineer biologically important glycostructures in a highly reproducible and time‐efficient manner.[^2^, ^3^] Although technological innovations have partially reconciled this disparity, achieving stereocontrol on solid phase remains a core challenge.^[4]^ In contrast to the automated synthesis of peptides and nucleic acids, each iterative glycan coupling forges a new C(sp^3^)−O bond with concomitant formation of a stereogenic center. Successful protocols are therefore contingent on being efficient and highly diastereoselective (α/β) to prevent mixtures being amplified across synthesis algorithms (Figure 1A). Emulating solution phase approaches, the most efficient strategies to regulate selectivity are encoded at the building block level through appropriate *O*‐based protecting group (PG) regimes.^[5]^ Whilst successful, this significantly limits the scope of AGA to naturally occurring glycans and presents a challenge for biomimetic design.^[6]^ Motivated by the medical importance of fluorinated sugars,^[7]^ the hydrolytic stability that C2 fluorination conveys,^[8]^ and the effectiveness of fluorine‐directed glycosylation by Anh–Eisenstein‐like induction models (stereoelectronic preference for the new σ‐bond to form *anti* to the C−F bond (low‐lying σ_C−F_*), see top insert Figure 1B),^[9]^ extending AGA to fluorinated modules would expand the current boundaries of glycospace. Inspired by the profusion of α‐linked mannose repeats units in human,^[10]^ bacterial,^[11]^ fungal^[12]^ and viral glycans,^[13]^ this motif was selected to validate fluorine‐directed automated glycan assembly (FDAGA) on a Glyconeer 2.1® synthesizer (Figures 1B/C).^[14]^

The selection of target structure was informed by the importance of bacterial mannan repeat units, which are structurally distinct from human glycans,^[11]^ and hold promise for vaccine development. Their human congeners, *N‐*glycans, decorate around 50 % of all proteins^[15]^ and are essential in cell signaling and trafficking.^[16]^ Furthermore, oligomannose structures have also recently emerged as promising antiviral vaccine candidates^[17]^ due to the high density of high‐mannose *N*‐glycans on the surfaces of HIV[^13a^, ^18^] and SARS‐CoV‐2.^[19]^ The multifaceted nature of this repeat unit, the venerable history of fluorinated glycans in mechanistic glycobiology,^[8]^ and the convenience of a ^19^F NMR probe,^[20]^ provided the impetus for reaction development. The elegant work by Delbianco and co‐workers in exploiting automated glycan assembly (AGA) to generate a library of singly fluorinated oligosaccharides (C3‐fluorinated glucose) for high throughput screenings,[^20a^, ^21^] provided further inspiration. To the best of our knowledge, the strategic deployment of (C2) fluorine‐directed glycosylation to address the pressing issue of stereoselectivity in AGA has not been reported.

## Results and Discussion

**Scheme 1:**
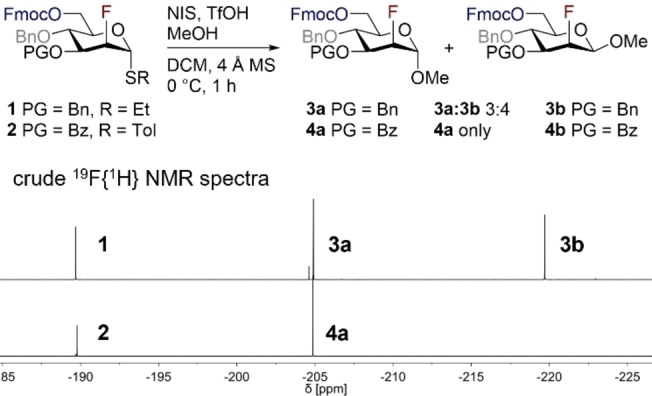
Evaluation of building block design for the AGA of C2‐fluorinated mannosides. Top: glycosylation of donors **1** and **2** to MeOH. Bottom: crude ^19^F{^1^H} NMR spectra.

To validate the conceptual framework in an automated glycan assembly paradigm, and cognizant of the prominence of C2‐fluorinated glycan mimetics,[^1d^, ^8c^, ^22^] fluorinated mannose building blocks were designed to supplement the existing portfolio of AGA‐primed monosaccharides (Scheme 1). A major challenge to be circumvented in the automated glycosylation of C2‐fluorinated carbohydrate donors is regulating the α‐diastereoselectivity in the absence of anchimeric assistance. Since a powerful synergy exists between the electronic nature of the protecting groups and the configuration at C2 in fluorine‐directed glycosylation,[^9a^, ^9b^] a systematic study of building block (BB) designs was undertaken to identify candidates that were compatible with AGA. BBs **1** and **2**, bearing orthogonal PGs commonly employed in AGA, were identified as the most promising candidates (Scheme 1).

**Scheme 2:**
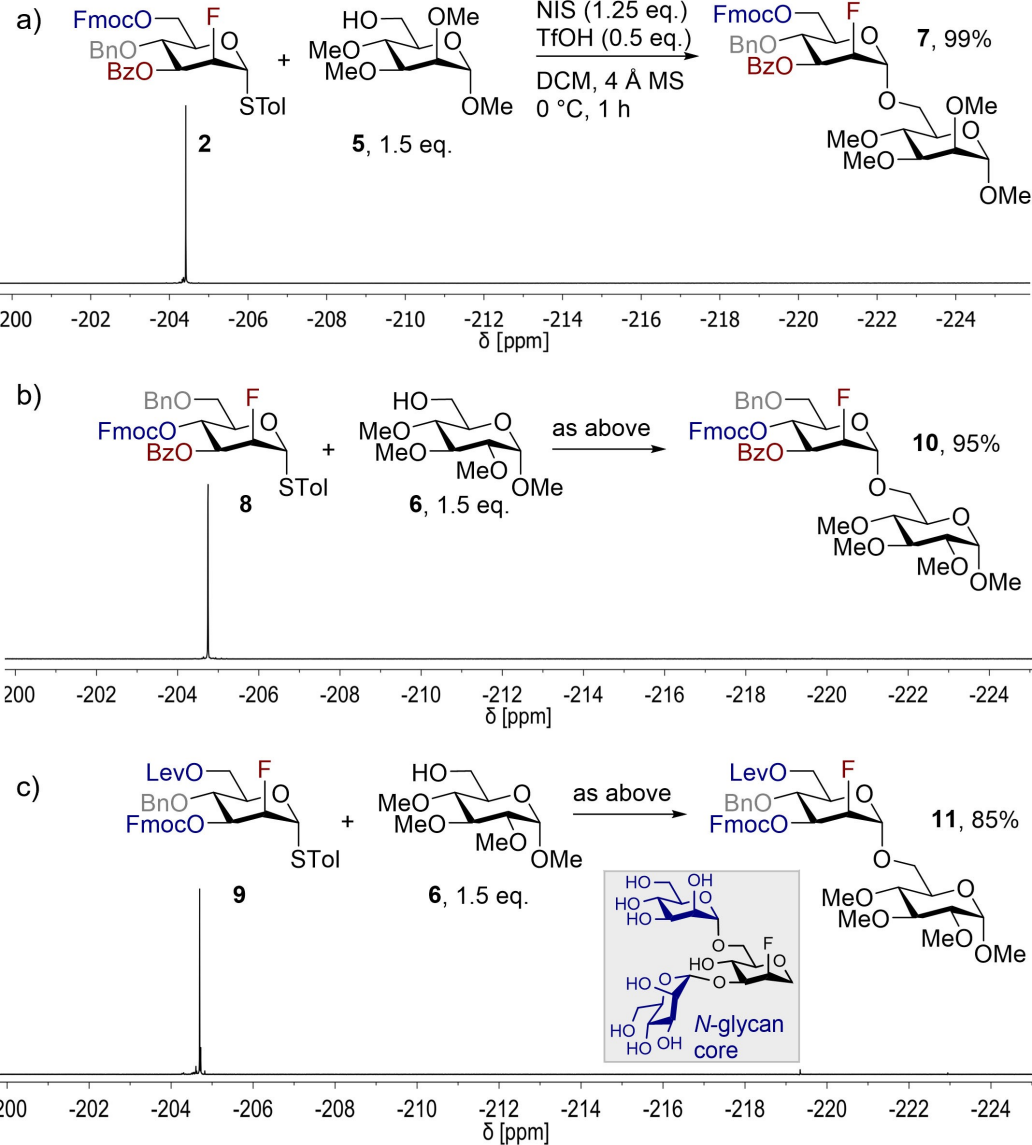
Building blocks for the construction of 1,6‐ (a), 1,4‐ (b) and 1,3‐ and 1,6‐glycosidic linkages (c). Crude ^19^F{^1^H} NMR spectra indicate that all building blocks undergo glycosylation in solution with complete α‐diastereoselectivity and complete conversion.

To glean an initial qualitative insight into their behavior, BBs **1** and **2** were subjected to solution phase glycosylations with MeOH or *i*PrOH as acceptors (NIS/TfOH, DCM, 4 Å MS, 0 °C, 1 h; then Na_2_S_2_O_3_, Scheme 1). The reaction outcome was evaluated based on the crude ^19^F{^1^H} NMR spectra, which revealed a distinct dependence of the diastereoselectivity on the BB design. Gratifyingly, BB **2** bearing a polar C3−Bz group furnished the α‐glycosylated product **4 a** exclusively, whereas BB **1** exhibited poor selectivity, with the β‐anomer **3 b** dominating (**3 a**:**3 b** 3 : 4). This difference in selectivity is a consequence of competing conformational preferences in the transient oxocarbenium ion.[^9b^, ^23^] Despite the promising selectivity, neither of the donors were fully converted to the desired glycoside (comparable reaction outcomes for MeOH and *i*PrOH), with the characteristic signal of the thiodonor (ca. −190 ppm) remained clearly visible in both scenarios (Scheme 1). Further optimization was then conducted with donor **2** until full conversion was achieved in a timeframe that was suitable for AGA (see the Supporting Information for details). Glycosylation of **2** with acceptor **5**, under modified conditions, proceeded smoothly to furnish disaccharide **7** with complete α‐selectivity and 99 % yield (Scheme 2a). Deprotection of the temporary Fmoc group and subsequent glycosylation also proceeded smoothly in solution (see Supporting Information for details). With optimized glycosylation conditions in hand (1.5 equiv acceptor, NIS (1.25 equiv), TfOH (0.5 equiv), DCM, 4 Å MS, 0 °C, 1 h; then Na_2_S_2_O_3_), the generality of the Fmoc/Bn/ester‐design was evaluated. BB **8**, to permit subsequent construction of 1,4‐glycosidic linkages, and orthogonally protected BB **9**, to enable the construction of 1,3‐ and 1,6‐glycosidic linkages such as those found in *N*‐glycans, both reacted smoothly with acceptor **6** to furnish the α‐glycosylated products **10** (95 %, Scheme 2b) and **11** (85 %, Scheme 2c) exclusively.

**Scheme 3:**
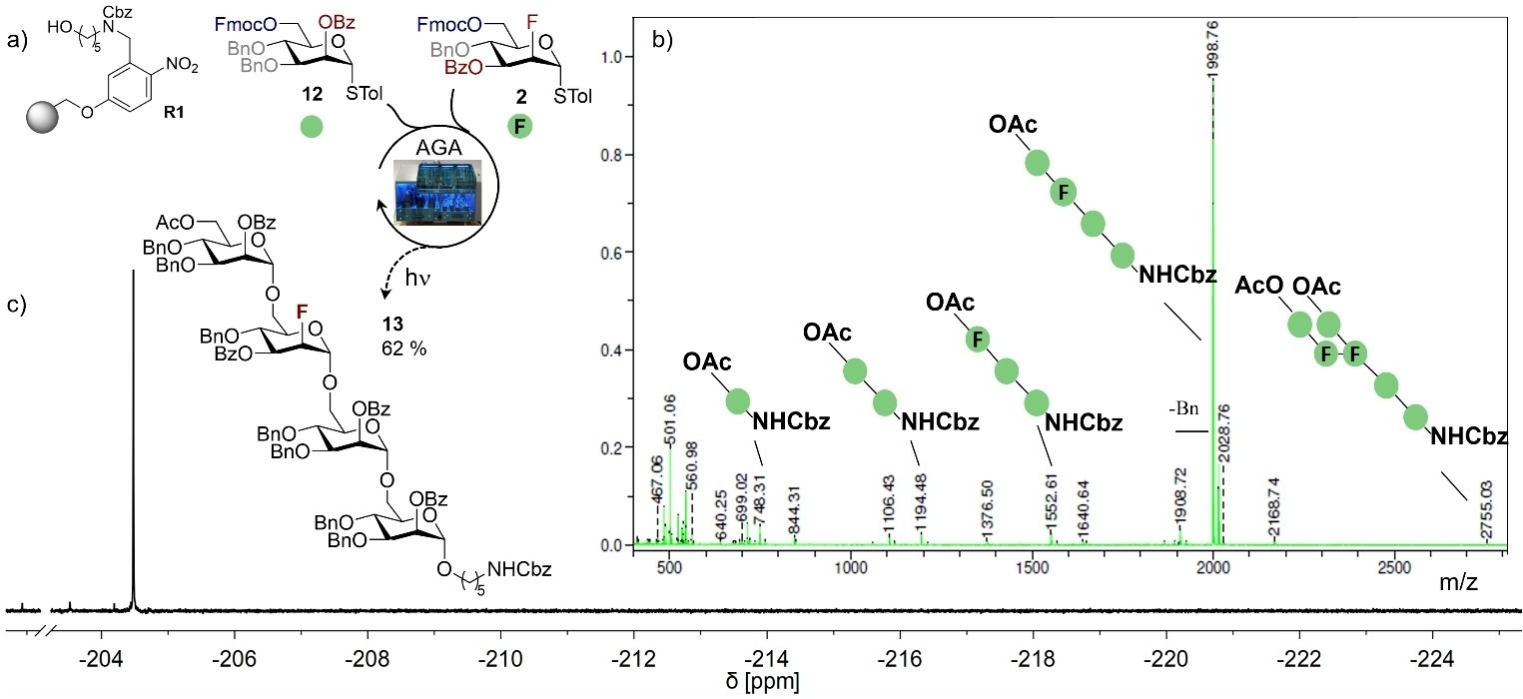
FDAGA of tetramannoside **13**. a) Automated assembly of fluorinated tetramannoside **13** on photocleavable Merrifield resin **R1**. Glycosylation conditions: donor **12** or **2** (8.0 equiv), NIS (1.5 equiv relative to donor), TfOH (0.4 equiv relative to donor), 0 °C for 5 min, 20 °C for 20 min. b) MALDI‐MS of the crude carbohydrate mixture after photocleavage reveals a clean reaction profile with only traces of deletion sequences. The structures of the identified species are indicated in symbol nomenclature (green circle=mannose). c) The crude ^19^F{^1^H} NMR spectrum of **13** shows no resonances in the β‐region (−215 to −225 ppm) indicating complete α‐selectivity.

**Scheme 4:**
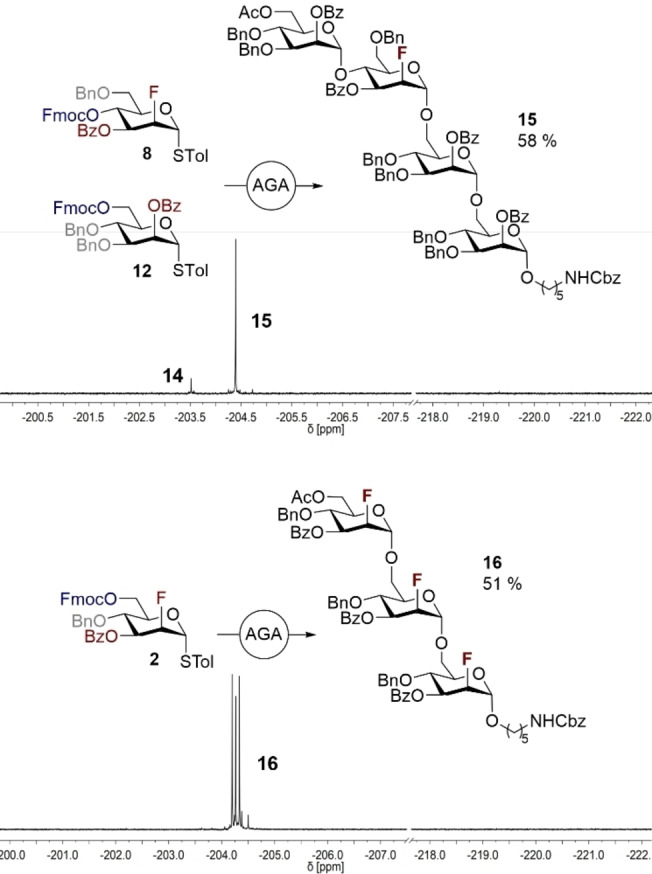
FDAGA of tetramannoside **15** and the trifluorinated trimannoside **16**. Selected parts of the α‐ and β‐regions of the crude ^19^F{^1^H} NMR spectra demonstrate the high levels of diastereoselectivity observed in coupling.

These conditions were then translated to an automation paradigm using a Glyconeer® 2.1 synthesizer. Following a brief process of optimization to integrate the well‐established, non‐fluorinated BB **12** (see Supporting Information for full details), the all α‐1,6‐linked tetramannoside **13** was selected as a first target structure (Scheme 3). The C2‐fluorinated BB **2** was smoothly glycosylated under identical conditions to those used for the non‐fluorinated species (donor (8.0 equiv), NIS (1.5 equiv relative to donor), TfOH (0.4 equiv relative to donor), 0 °C for 5 min, 20 °C for 20 min). Compound **13** was successfully assembled on photocleavable Merrifield resin **R1** and the overall efficiency was evaluated by MALDI‐MS and ^19^F{^1^H} NMR analysis of the crude glycan mixture after photocleavage using a procedure previously reported by this group.^[23]^ Gratifyingly, a clean reaction profile was revealed by MALDI‐MS, yielding the desired tetramannoside **13** as the major reaction product. The satisfactory performance of C2‐fluorinated BB **2** in AGA was also confirmed by ^19^F{^1^H} NMR analysis, showing a single resonance of the major fluorinated species (94 % of all fluorinated species in the crude mixture) corresponding to **13**. Importantly, no resonance was observed in the β‐region (−215 to −225 ppm) of the spectrum, allowing a diastereoselectivity of >88 : 1 to be established based on the ^19^F{^1^H} NMR spectrum. Collectively these data indicate that the fluorinated BB **2** and the parent BB **12** show comparable levels of diastereoselectivity and reactivity as both donors and acceptors in AGA. This is an encouraging validation of fluorine‐directed glycosylation on solid support. Similar results were obtained for BB **8** in the construction of tetramannoside **15**, thereby extending the generality of the concept to use C2‐fluorinated BBs for fluorine‐directed AGA (Scheme 4, top). Although traces of the doubly fluorinated hexasaccharide product **14** were detected from the initial exploratory work (ratio **15 : 14** 22 : 1 by ^19^F NMR), the un‐desired β‐diastereomer (around −220 ppm) was never detected, and it was possible to isolate **15** in 58 % yield over ten steps.

As a proof of concept for the AGA of multiply fluorinated oligosaccharides, homotrimer **16** was synthesized in a single operation. MALDI‐MS and ^19^F{^1^H} NMR analysis again revealed the smooth reaction of BB **2**, enabling the target trimannoside to be generated in 87 % yield after cleavage from the solid support and with complete (α‐)diastereocontrol.

**Scheme 5:**
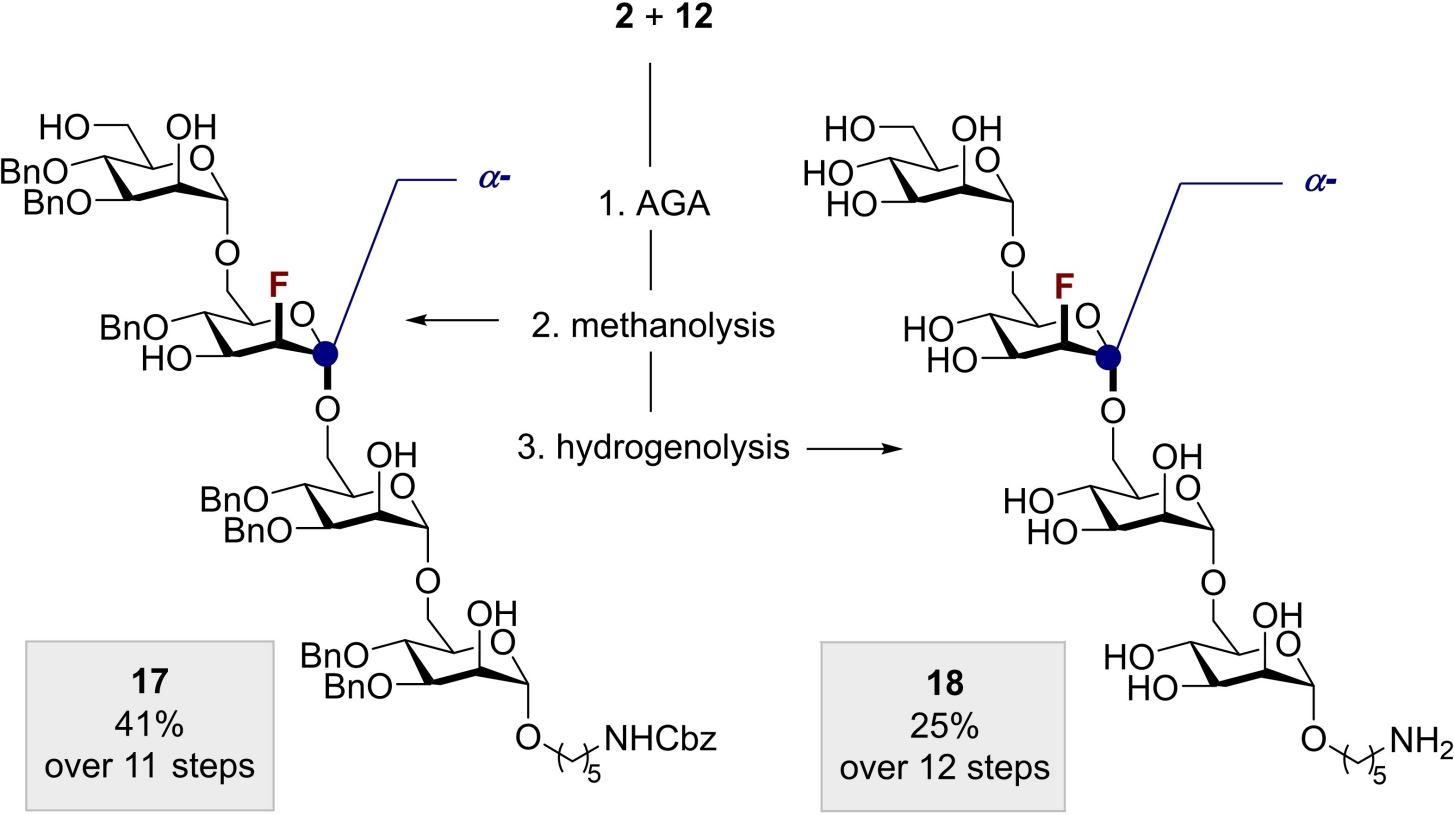
Deprotection of fluorinated oligosaccharides **17** and **18** prepared by FDAGA.

To further demonstrate the synthetic utility of C2‐fluorinated BBs in AGA, standard downstream manipulations (purification and global deprotection) were integrated into the sequence (Scheme 5). Purification by normal‐phase size exclusion chromatography (SEC) afforded **13** (from **2**+**12**) in 62 % yield (15.2 mg). NMR structural characterization confirmed the α‐1,6‐connectivity of **13**, and subsequent solvolysis under Zemplén conditions (NaOMe, MeOH) proceeded smoothly to afford semi‐protected tetrasaccharide **17** in 67 % yield (41 % based on resin, see Scheme 5). The final hydrogenolysis of the remaining PGs required the use of pre‐treated Pd(C) for the deprotection of complex saccharides.^[25]^ The global deprotection could be performed directly from crude **13** to afford 2.3 mg (25 % based on resin) of fluorinated tetramannoside **18** after HPLC purification. These results compare well to the previously reported yields (16 %)^[20a]^ of C3‐fluorinated tetrasaccharides produced by AGA without intermediate purification. Similar results were obtained for the purification and deprotection of **15** and **16** (please see the Supporting Information for full experimental details), thereby demonstrating the utility of fluorine‐directed glycosylation in the automated preparation of site‐selectively modified glycans.

**Figure 2:**
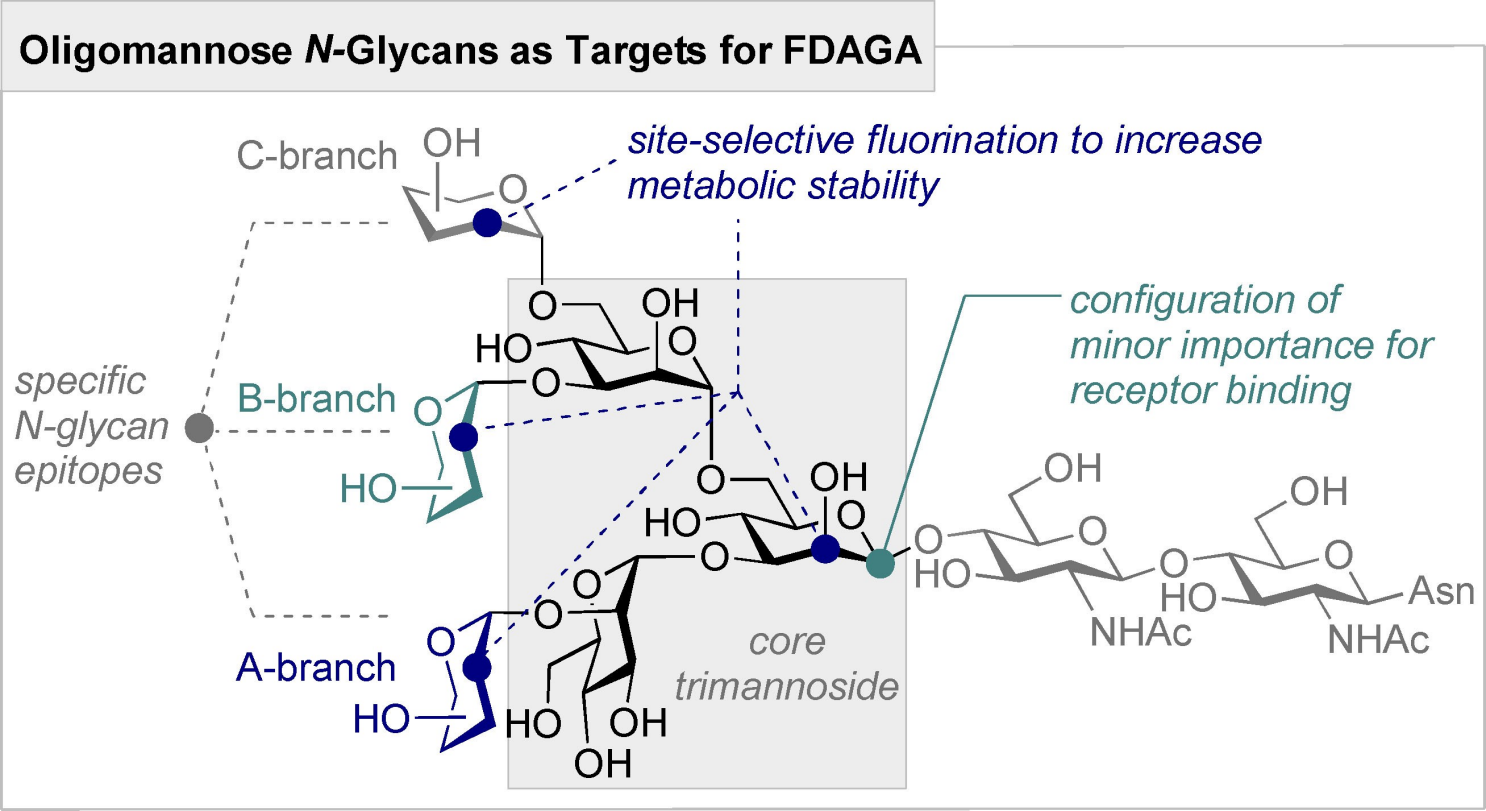
Design considerations for the FDAGA of *N*‐glycan oligomannosides.

Having validated C2‐fluorinated building blocks for the synthesis of linear glycans by FDAGA, preliminary studies towards the synthesis of branched bioactive oligomannosides were initiated. Cognizant of the ubiquity of oligomannosides in human[^10^, ^15^] and viral *N*‐glycans,^[13]^ and their importance for HIV vaccine development,^[17]^ selectively fluorinated α‐Man(1→3)[α‐Man(1→6)]‐α‐Man glycans were selected as target structures for FDAGA (Figure 2). Site selective fluorine introduction at the pivotal branching core of the trimannoside, and on the peripheral branch termini, would be highly enabling on account of the metabolic liabilities that these units present in vitro and in vivo.

To establish the versatility of building blocks **2** and **9** in accessing both linear and branched targets by FDAGA, the doubly fluorinated pentamannoside **20** was conceived as a target: this F‐glycan contains the core trimannoside (see Figure 2) and the full A‐branch of high‐mannose type *N*‐glycans. Specifically, the fluorination of the reducing end branching unit and the non‐reducing end terminal residue of the A‐branch serves two purposes: firstly, fluorination provides a ^19^F NMR probe for binding studies and epitope mapping.^[20]^ Previous studies have established the enhanced binding of the linear A‐, B‐ and C‐branches relative to the core trimannoside (Figure 2).[^17a^, ^18^] The configuration of the reducing‐end plays only a minor role in DC‐SIGN recognition.^[26]^ Secondly, fluorination of the core and terminal positions is anticipated to increase the hydrolytic stability of the oligomannoside.^[8]^ To date, the limited serum stability of high‐mannose type *N‐*glycans continues to impede attempts to raise monoclonal antibodies in vivo rendering this scaffold highly relevant.^[27]^ It is important to note that site‐selective fluorination has been shown to increase the immunogenicity of glycan‐based conjugate vaccines while retaining antibody cross‐reactivity.^[28]^

**Scheme 6:**
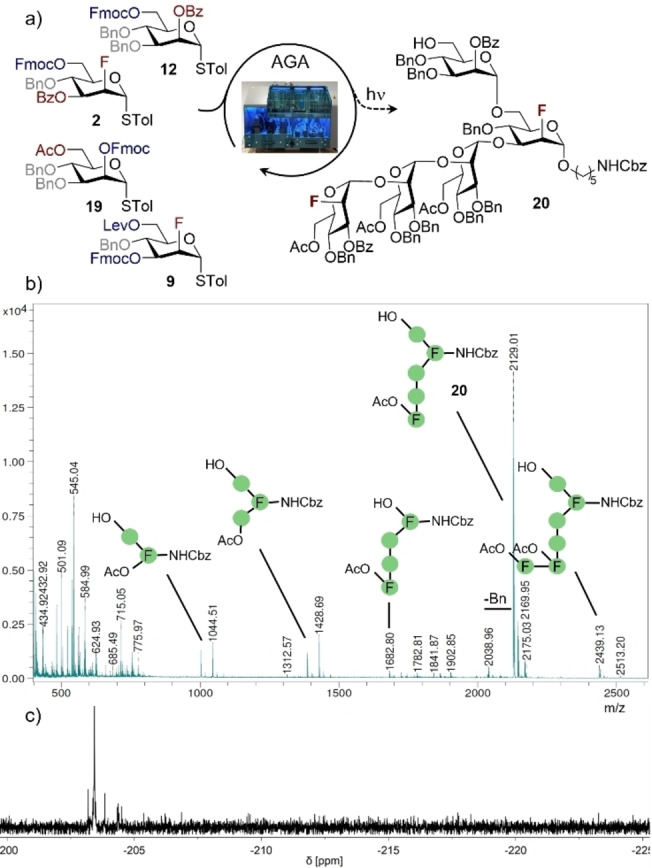
FDAGA of the doubly fluorinated *N*‐glycan core pentamannoside **20**. a) Automated assembly of **20** on photocleavable Merrifield resin. For glycosylation conditions, see the Supporting Information. b) MALDI‐MS of the crude mixture after photocleavage shows the target structure as the major product. The structures of the identified species are indicated in symbol nomenclature (green circle=mannose). c) The crude ^19^F{^1^H} NMR spectrum of **20** shows no resonances in the β‐region (−215 to −225 ppm), indicating complete α‐selectivity.

To that end, pentasaccharide **20** was assembled from monosaccharides **9**, **19**, **2** and **12** on photolabile Merrifield resin (Scheme 6a). MALDI‐MS and NMR analyses confirm the successful synthesis of **20** (see Schemes 6b and c). From this case study, it was noted that the C2‐fluorinated scaffolds proved to be less challenging in the automated assembly of fluorinated *N*‐glycan mimetics than the non‐fluorinated building blocks. In the case of the non‐fluorinated mannose donors, it was possible to identify trace deletion sequences resulting from incomplete glycosylation. In stark contrast, the fluorinated BBs reacted with full α‐selectivity and high efficiency (Scheme 6b). Optimisation and further exploitation of FDAGA for the construction of bioactive mannosides are currently under investigation in our laboratory and will be reported in due course.

## Conclusion

In addressing the persistent challenge of stereocontrol in automated glycan assembly, the C(sp^3^)−F bond has been validated as a steering group to regulate the anomeric configuration for the synthesis of linear and branched targets. Through judicious building block design, C‐2 fluorinated mannose modules can be integrated into established automation protocols to expand the scope of this enabling technology. Reactions are characterized by extremely high levels of efficiency and selectivity (>88 : 1 α:β), and a comparative analysis demonstrates that the C−F bond is an excellent substitute for the C−OBz motif (**8** and **12**) in directing the nucleophile to the α‐face. Given the prominence of fluorine in molecular editing, and the ubiquity of oligomannoses in human biomedicine, it is envisaged that this enabling advance will be effortlessly integrated into automated synthesis algorithms.

## Conflict of interest

The authors declare no conflict of interest.

1

## Supporting information

As a service to our authors and readers, this journal provides supporting information supplied by the authors. Such materials are peer reviewed and may be re‐organized for online delivery, but are not copy‐edited or typeset. Technical support issues arising from supporting information (other than missing files) should be addressed to the authors.

Supporting Information

## Data Availability

The data that support the findings of this study are available from the corresponding author upon reasonable request.
