# Supplementary material for: Fluorine‐Directed Automated Mannoside Assembly
Source: Angew Chem Int Ed Engl. 2022 Dec 12;62(3):e202213304. doi: 10.1002/anie.202213304 (PMC10108063; doi:10.1002/anie.202213304)

Angewandte Chemie
Eine Zeitschrift der Gesellschaft Deutscher Chemiker

Supporting Information

Fluorine-Directed Automated Mannoside Assembly

*C. S. Teschers, R. Gilmour**

Supporting Information
©Wiley-VCH 2022
69451 Weinheim, Germany

Fluorine-Directed Automated Mannoside Assembly

Charlotte S. Teschers and Ryan Gilmour*

DOI: 10.1002/anie.202213304

Table of Contents

General Information S3
Experimental Procedures S4
Building Block Syntheses S4
Evaluation of the Building Blocks in Solution S10
Fluorine-Directed Automated Glycan Assembly (FDAGA) S14
Stock Solutions for AGA S14
Modules for AGA S15
Post-Automation Manipulations S15
Global deprotection S16
Automated Syntheses S16
References S23
NMR Spectra S24

General Information

All chemicals were purchased as reagent grade and used without further purification unless stated otherwise. *N*-iodosuccinimide (NIS, 97 % purity) was purchased from ABCR and recrystallised from benzene/dioxane. Building block **19** was purchased from GlycoUniverse and used as delivered. Solvents for purification (extraction and chromatography) were purchased as technical grade and distilled on the rotary evaporator prior to use. Dry solvents were taken from a custom-build chromatographic solvent drying system. For column chromatography SiO2 (40-63 µm for Flash-Chromatography, VWR Chemicals) was used as stationary phase. Analytical thin layer chromatography (TLC) was performed on aluminum foil pre-coated with SiO2-60 F254 (Merck) and visualised with a UV-lamp (254 nm) and CAM solution. Concentration in vacuo was performed at ~10 mbar and 42 °C, drying at ~10-2 mbar and room temperature. NMR spectra were measured by the NMR service of the Organisch-Chemisches Institut, Westfaelische Wilhelms-Universitaet Muenster on a Bruker BZH 200/52, Bruker AV300, Bruker AV400, Agilent DD2 500 or an Agilent DD2 600 spectrometer at room temperature. The chemical shifts are referenced to the residual solvent peak as internal standard (CDCl3 δH = 7.26 ppm, δC = 77.16 ppm; CD2Cl2 δH = 5.32 ppm, δC = 54.00 ppm; CD3OD δH = 3.31 ppm, δC = 49.00 ppm; D2O δH = 4.79 ppm). The resonance multiplicity is abbreviated as: s (singlet), d (doublet), t (triplet), q (quadruplet), p (pentet), sext (sextet), sep (septet), m (multiplet) and br (broad). Assignments of unknown compounds are based on COSY (HH), HMBC, HSQC, TOCSY and NOESY spectra. Melting points were measured on a Büchi B-545 melting-point apparatus in open capillaries. IR spectra were recorded on a Perkin-Elmer 100 FT-IR spectrometer, selected adsorption bands are reported in wavenumbers (cm–1) and intensities are reported as: w (weak), m (medium), s (strong) and br (broad). High-resolution mass spectra (HRMS-ESI) and MADLI-MS spectra were measured by the MS service of the Organisch-Chemisches Institut, Westfaelische Wilhelms-Universitaet Muenster on a Thermo Fisher Orbitrap LTQ XL, a Thermo Fisher Orbitrap Velos Pro or a Bruker Autoflex Speed MALDI-TOF spectrometer. Optical rotations were measured on a Perkin-Elmer 341 polarimeter. Flow photoreactions were performed using a custom-build 365 nm LED flow reactor. The photoreactor comprises eight LEDs (LED Engin LZ4-04UV00, 2000-3800 mW radiant flux @ 750 mA), which are dimmed to 350 mA.[1]
Automated glycan syntheses were performed with a Glyconeer2.1TM using the GlycoSoft software. Dry DCM and dry 1,4-dioxane were used to dissolve building blocks and prepare reagent solutions for AGA. DMF, THF and MeOH for AGA were used as HPLC grade. Building blocks were co-evaporated three times with dry toluene and dried in vacuo for minimum 1 h prior to use. All reagents were freshly prepared and kept under argon during the automated syntheses. Solutions B and C were kept at ca. 2 °C during the automation run, all other reagents, building blocks and solvents were stored at room temperature. All automated syntheses were performed on a 12.5 µmol scale.
For flash chromatography, a Büchi Pure C-850 FlashPrep system and Büchi FlashPure ID Silica cartridges with CyH/EtOAc or CyH/iPrOH gradients were used. For preparative HPLC, a Büchi Pure C-850 FlashPrep system or an Agilent Infinity 1260 HPLC system and Zorbax Eclipse Plus C18 (3.5 µm, 4.6 x 100 mm) columns with hexanes/*i*PrOH (normal phase) or water/MeCN gradients were used. Detection was either performed at 210 nm or using an ELSD.

Experimental Procedures

Functionalised Merrifield resin **R1** and building block **12** were synthesized as described previously.[1] 1,3,4,6-Tetra-*O*-acetyl-2-deoxy-2-fluoro-α-D-mannopyranoside (**S1**) was synthesised according to a previously reported procedure.[2]

Building Block Syntheses

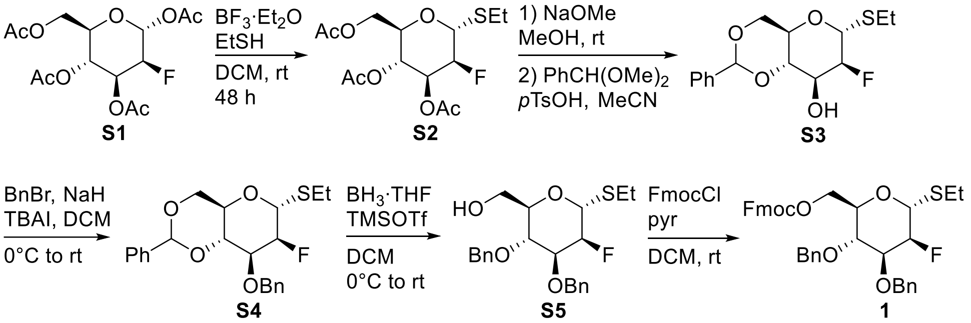

Ethyl 3,4,6-tri-*O*-acetyl-2-deoxy-2-fluoro-1-thio-α-D-mannopyranoside (S2)

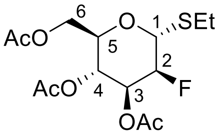

S2

A solution of **S1** (3.97 g, 11.6 mmol, 1.0 eq.), ethanethiol (1.0 mL, 14 mmol, 1.2 eq.) and $BF_3 \cdot Et_2O$ (4.3 mL, 35 mmol, 3.0 eq.) in dry DCM (30 mL) was stirred at rt for 2 d. The pale orange solution was carefully quenched with sat. aqueous $NaHCO_3$ and the aqueous layer was extracted with DCM (3 x 15 mL). The combined organic layers were dried over $MgSO_4$ and the volatiles were removed by distillation. The residue was purified by column chromatography (SiO_2, CyH/EtOAc 9/1) to afford **S2** as a pale-yellow syrup (2.04 g, 5.79 mmol, 50 %) as a 9:1 mixture with the 1,2-dithio glucose derivative.

Due to the impurities, no complete spectroscopic data were collected. The resonances in the ^{1}H-NMR were assigned based on the observed chemical shifts and coupling constants. **R$_f$** (CyH/EtOAc 2/1) 0.47; **^{1}H NMR** (400 MHz, $CDCl_3$) δ 5.46 (br d, J_{FH} = 15.1 Hz, 1H, H-C1), 5.36 (t, J = 10 Hz, 1H, H-C4), 5.15 (dd, J_{FH} = 28.3 Hz and J = 10 Hz, 1H, H-C3), 4.89 (dd, J_{FH} = 50.0 Hz and J = 2.0 Hz, 1H, H-C2), 4.36 (ddd, J = 9.4, 5, 2 Hz, 1H, H-C5), 4.31 (br dd, J = 12.2, 5 Hz, 1H, H-C6), 4.10 (br d, J = 12.1 Hz, 1H, H-C6), 2.68 (m, 2H, Et), 2.08 (s, 3H, Ac), 2.08 (s, 3H, Ac), 2.04 (s, 3H, Ac), 1.32 (t, J = 7.4 Hz, 3H, Et) ppm; **^{19}F{^{1}H} NMR** (376 MHz, $CDCl_3$) δ -189.79 ppm; **HRMS** (ESI) m/z $[M+Na]^+$ calcd for $C_{14}H_{21}FO_7SNa^+$ 375.0884; found 375.0899.

Ethyl 4,6-*O*-benzylidene-2-deoxy-2-fluoro-1-thio-α-D-mannopyranoside (S3)

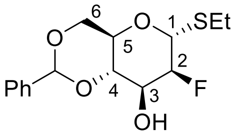

S3

NaOMe (123 mg, 2.28 mmol, 0.4 eq.) was added to a solution of **S2** (2.01 g, 5.71 mmol, 1.0 eq.) in MeOH (30 mL) at rt and stirred overnight. The yellow solution was neutralised with Amberlyst[H^+]®, concentrated *in vacuo* and the residue was co-evaporated with toluene (2 x) to remove residual methanol. The residue was dissolved in acetonitrile and benzaldehyde dimethyl acetal (1.70 mL, 11.4 mmol, 2.0 eq.) and *p*-toluenesulfonic acid (98 mg, 0.57 mmol, 0.1 eq.) were added. After stirring overnight, the greenish solution was quenched with NEt_3 (0.15 mL), concentrated *in vacuo* and purified by column chromatography (SiO_2, CyH/EtOAc 19/1) to afford **S3** as a yellow oil (1.11 g, 3.53 mmol, 62 %) as a mixture with a side product.

Due to the impurities, no complete spectroscopic data were collected. The resonances in the ^{1}H-NMR were assigned based on the observed chemical shifts and coupling constants. **R$_f$** (CyH/EtOAc 4/1) 0.25; **^{1}H NMR** (400 MHz, CD_2Cl_2) δ 7.52 – 7.36 (m, 5H, benzylidene), 5.58 (s, 1H, C*H*Ph), 5.46 (dd, J_{FH} = 16.1 Hz and J = 1.5 Hz, 1H, H-C1), 4.88 (ddd, J_{FH} = 49.4 Hz and J = 2.5, 1.5 Hz, 1H, H-C2), 4.31 – 4.15 (m, 2H, H-C5, H-C6), 4.03 (ddd, J_{FH} = 27.2 Hz and J = 10.2, 2.5 Hz, 1H, H-C3), 3.96 – 3.83 (m, 1H, H-C4), 3.89 – 3.78 (m, 1H, H-C6), 2.84 – 2.59 (m, 2H, Et), 2.54 (br s, 1H, OH), 1.31 (t, J = 7.4 Hz, 3H, Et) ppm; **^{19}F{^{1}H } NMR** (376 MHz, CD_2Cl_2) δ -190.52 ppm; **HRMS** (ESI) m/z $[M+Na]^+$ calcd for $C_{15}H_{19}FO_4SNa^+$ 337.0880; found 337.0892.

Ethyl 3-*O*-benzyl-4,6-*O*-benzylidene-2-deoxy-2-fluoro-1-thio-α-D-mannopyranoside (S4)

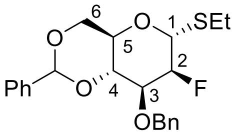

S4

To a solution of **S3** (585 mg, 1.86 mmol, 1.0 eq.) in dry DCM (20 mL) were added NaH (60% dispersion in mineral oil, 111 mg, 2.79 mmol, 1.5 eq.), TBAI (68 mg, 0.18 mmol, 0.1 eq.) and benzyl bromide (0.37 mL, 2.79 mmol, 1.5 eq.) at 0 °C under argon. After stirring overnight (16 h), the reaction was quenched with water. The organic layer was dried over $MgSO_4$ and concentrated *in vacuo*. The residue was purified by column chromatography (SiO_2, CyH → CyH/EtOAc 9/1) to afford **S4** as a yellow oil (748 mg, 1.85 mmol, 99%).

$[\alpha]_D^{24}$ +156.7 (c = 1.0, CH_2Cl_2); **m.p.** 85 °C; **^{1}H NMR** (599 MHz, CD_2Cl_2) δ 7.51 – 7.47 (m, 2H, Bn), 7.41 – 7.37 (m, 3H, Bn), 7.37 – 7.27 (m, 5H, benzylidene), 5.63 (s, 1H, C*H*Ph), 5.44 (dd, J_{FH} = 15.8 Hz and *J* = 1.6 Hz, 1H, H-C1), 4.91 (ddd, J_{FH} = 49.2 Hz and *J* = 2.6, 1.6 Hz, 1H, H-C2), 4.80 (d, *J* = 11.7 Hz, 1H, C*H*$_2$Ph), 4.70 (d, *J* = 11.7 Hz, 1H, C*H*$_2$Ph), 4.23 (dd, *J* = 9.7, 4.9 Hz, 1H, H-C6), 4.21-4.17 (m, 1H, H-C5), 4.11 (td, *J* = 9.7 Hz, J_{FH} = 1.5 Hz, 1H, H-C4), 3.88 (ddd, J_{FH} = 27.5 Hz and *J* = 9.9, 2.6 Hz, 1H, H-C3), 3.90 – 3.82 (m, 1H, H-C6), 2.76 – 2.60 (m, 2H, Et), 1.30 (t, *J* = 7.4 Hz, 3H, Et) ppm; **^{13}C NMR** (151 MHz, CD_2Cl_2) δ 138.6 (Bn), 138.2 (benzylidene), 129.5 (Bn), 128.9 (benzylidene), 128.7 (Bn), 128.4 (benzylidene), 128.3 (benzylidene), 126.7 (Bn), 102.3 (benzylidene), 90.9 (d, *J* = 186.3 Hz, C2), 83.9 (d, *J* = 23.8 Hz, C1), 79.4 (d, *J* = 2.3 Hz, C4), 75.4 (d, *J* = 17.2 Hz, C3), 73.4 (CH_2Ph), 69.1 (C6), 65.1 (C5), 26.1 (Et), 15.3 (Et) ppm; **^{19}F NMR** (564 MHz, CD_2Cl_2) δ -189.82 (ddd, J_{FH} = 49.2, 27.4, 15.9 Hz) ppm; **IR** (neat) $\tilde{\nu}_{max}$ / cm^{-1} 2877 (w), 1455 (w), 1376 (m), 1344 (w), 1283 (w), 1216 (w), 1165 (w), 1147 (w), 1096 (s), 1028 (m), 994 (s), 979 (s), 912 (m), 870 (m), 834 (w), 804 (m), 791 (m), 751 (s), 694 (s), 667 (m); **HRMS** (ESI) m/z $[M+Na]^+$ calcd for $C_{22}H_{25}FO_4SNa^+$ 427.1350; found 427.1359.

Ethyl 3,4-di-*O*-benzyl-2-deoxy-2-fluoro-1-thio- α -D-mannopyranoside (S5)

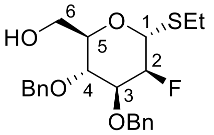

S5

Borane (1 M in THF, 3.7 mL, 3.7 mmol, 3.0 eq.) and TMSOTf (0.11 mL, 0.61 mmol, 0.5 eq.) were successively added to a solution of **S4** (497 mg, 1.23 mmol, 1.0 eq.) in dry DCM (13 mL) at 0 °C under argon. After stirring for 8 hours at 0 °C, the mixture was quenched with NEt_3 (0.2 mL) and methanol (0.15 mL). The solution was washed with water and brine, dried over $MgSO_4$ and concentrated *in vacuo*. The residue was purified by column chromatography (SiO_2, CyH/EtOAc 19/1) to afford **S5** as a colourless semi solid (431 mg, 1.06 mmol, 86 %).

$[\alpha]_D^{23}$ +102.2 (c = 0.75, $CHCl_3$); **^{1}H NMR** (599 MHz, $CDCl_3$) δ 7.41 – 7.29 (m, 10H, Bn), 5.39 (dd, J_{FH} = 14.9 Hz and *J* = 1.8 Hz, 1H, H-C1), 4.92 (d, *J* = 11.0 Hz, 1H, C*H*$_2$Ph), 4.82 (dt, J_{FH} = 49.9 Hz and *J* = 2.1 Hz, 1H, H-C2), 4.74 (d, *J* = 11.6 Hz, 1H, C*H*$_2$Ph), 4.70 (d, *J* = 11.6 Hz, 1H, C*H*$_2$Ph), 4.67 (d, *J* = 11.0 Hz, 1H, C*H*$_2$Ph), 4.01 (dt, *J* = 9.7, 3.5 Hz, 1H, H-C5), 3.93 (t, *J* = 9.5 Hz, 1H, H-C4), 3.82 (ddd, J_{FH} = 29.4 Hz and *J* = 9.4, 2.4 Hz, 1H, H-C3), 3.83 – 3.77 (m, 2H, H-C6), 2.71 – 2.56 (m, 2H, Et), 1.28 (t, *J* = 7.4 Hz, 3H, Et); **^{13}C NMR** (151 MHz, $CDCl_3$) δ 138.2 (Bn), 137.8 (Bn), 128.7 (Bn), 128.6 (Bn), 128.2 (Bn), 128.11 (Bn), 128.10 (Bn), 128.0 (Bn), 88.9 (d, *J* = 184.9 Hz, C2), 82.4 (d, *J* = 22.6 Hz, C1), 79.0 (d, *J* = 18.0 Hz, C3), 75.5 (CH_2Ph), 74.4 (d, *J* = 1.7 Hz, C4), 72.6 (C5), 72.4 (CH_2Ph), 62.1 (C6), 25.6 (Et), 15.0 (Et) ppm; **^{19}F NMR** (564 MHz, $CDCl_3$) δ -189.67 (ddd, J_{FH} = 50.0, 29.4, 14.9 Hz) ppm; **IR** (neat) $\tilde{\nu}_{max}$ / cm^{-1} 3487 (w), 2927 (w), 2873 (w), 1497 (w), 1455 (m), 1370 (w), 1351 (w), 1209 (m), 1091 (s), 1069 (s), 1027 (s), 967 (s), 912 (s), 882 (s), 792 (m), 734 (2), 732 (s), 696 (s), 667 (m); **HRMS** (ESI) m/z $[M+Na]^+$ calcd for $C_{22}H_{27}FO_4SNa^+$ 429.1506; found 429.1522:

Ethyl 3,4-di-*O*-benzyl-6-*O*-fluorenylmethoxycarbonyl-2-deoxy-2-fluoro-1-thio-α-D-mannopyranoside (1)

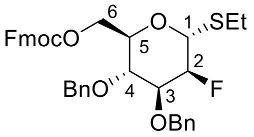

1

FmocCl (400 mg, 1.55 mmol, 1.5 eq.) and pyridine (0.25 mL, 3.10 mmol, 3.0 eq.) were added to a solution of **S5** (407 mg, 1.03 mmol, 1.0 eq.) in DCM (20 mL) at rt and stirred for 3 days. The mixture was washed with 1 m HCl (2 x 20 mL) and brine. The organic layer was dried over $MgSO_4$ and concentrated *in vacuo*. The residue was purified by column chromatography (SiO_2, CyH/EtOAc 19/1) to afford **1** as a colourless semi solid (578 mg, 0.914 mmol, 88 %).

$[\alpha]_D^{24}$ +105.3 (c = 1.0, $CHCl_3$); **^{1}H NMR** (599 MHz, $CDCl_3$) δ 7.78 – 7.71 (m, 2H, Fmoc), 7.69 – 7.56 (m, 2H, Fmoc), 7.44 – 7.27 (m, 14H, Fmoc, Bn), 5.44 (dd, J_{FH} = 14.9 Hz and *J* = 1.7 Hz, 1H, H-C1), 4.94 (d, *J* = 10.9 Hz, 1H, C*H*$_2$Ph), 4.84 (dt, J_{FH} = 49.9 Hz and *J* = 2.1 Hz, 1H, H-C2), 4.75 (d, *J* = 11.5 Hz, 1H, C*H*$_2$Ph), 4.70 (d, *J* = 11.6 Hz, 1H, C*H*$_2$Ph), 4.63 (d, *J* = 10.9 Hz, 1H, C*H*$_2$Ph), 4.45 (dd, *J* = 11.7, 2.4 Hz, 1H, H-C6), 4.41 (dd, *J* = 11.6, 4.9 Hz, 1H, H-C6), 4.40 (dd, *J* = 10.4, 7.6 Hz, 1H, C*H*$_2$ Fmoc), 4.35 (dd, *J* = 10.4, 7.6 Hz, 1H, C*H*$_2$ Fmoc), 4.25 (t, *J* = 7.7 Hz, 1H, C*H* Fmoc), 4.25 (ddd, *J* = 9.7, 4.9, 2.4 Hz, 1H, H-C5), 3.92 (t, *J* = 9.4 Hz, 1H, H-C4), 3.84 (ddd, J_{FH} = 29.1 Hz and *J* = 9.3, 2.4 Hz, 1H, H-C3), 2.74 – 2.57 (m, 2H, Et), 1.28 (t, *J* = 7.4 Hz, 3H, Et) ppm; **^{13}C NMR** (151 MHz, $CDCl_3$) δ 155.2 (C=O, Fmoc), 143.6 (Fmoc), 143.5 (Fmoc), 141.44 (Fmoc), 141.41 (Fmoc), 138.0 (Bn), 137.6 (Bn), 128.7 (Bn), 128.6 (Bn), 128.3 (Bn), 128.18 (Bn), 128.15 (Bn), 128.1 (Bn), 128.0 (Fmoc), 127.33 (Fmoc), 127.31 (Fmoc), 125.43 (Fmoc), 125.35 (Fmoc), 120.2 (Fmoc), 88.5 (d, *J* = 185.6 Hz, C2), 82.3 (d, *J* = 22.8 Hz, C1), 79.2 (d, *J* = 18.2 Hz, C3), 75.5 (CH_2Ph), 74.4 (d, *J* = 1.4 Hz, C4), 72.3 (CH_2Ph), 70.4 (C5), 70.1 (CH_2 Fmoc), 66.7

(C6), 46.8 (CH Fmoc), 25.6 (Et), 15.1 (Et) ppm; **^{19}F NMR** (564 MHz, $CDCl_3$) δ -189.76 (ddd, J_{FH} = 49.9, 29.1, 15.0 Hz) ppm; **IR** (neat) $\tilde{\nu}_{max}$ / cm^{-1} 3035 (w), 2919 (w), 1743 (s), 1497 (w), 1478 (w), 1450 (m), 1412 (w), 1384 (w), 1348 (w), 1329 (m), 1281 (s), 1255 (s), 1103 (s), 1035 (m), 968 (m), 951 (m), 911 (w), 874 (w), 789 (m), 755 (m), 733 (s), 697 (s), 673 (m); **HRMS** (ESI) m/z $[M+Na]^+$ calcd for $C_{37}H_{37}FO_6SNa^+$ 651.2187; found 651.2187.

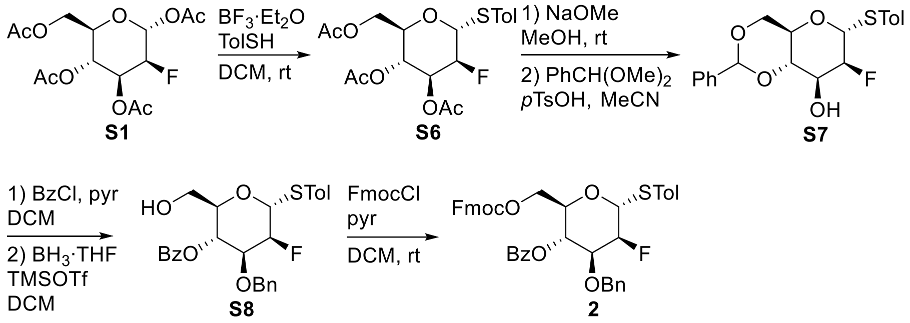

4-Methylphenyl 3,4,6-tri-*O*-acetyl-2-deoxy-2-fluoro-1-thio-α-D-mannopyranoside (S6)

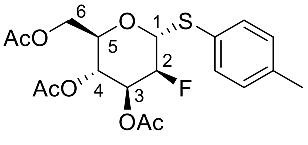

S6

S1 (5.700 g, 16.27 mmol, 1.0 eq.) and *p*-toluenethiol (2.63 g, 21.2 mmol, 1.3 eq.) were dissolved in dry DCM (60 mL) and cooled to 0 °C. Boron trifluoride diethyl etherate (6.0 mL, 6.0 mmol, 3.0 eq.) was added slowly and the mixture was allowed to warm to rt while stirring for 3 d. The brown solution was quenched by careful addition of sat. aqueous $NaHCO_3$, and the aqueous layer was extracted with DCM (3 x 80 mL). The combined organic layers were washed with brine, dried over $MgSO_4$ and concentrated *in vacuo*. The crude product was purified by column chromatography (SiO_2, CyH/EtOAc 9/1 → 7/1) to afford thioglycoside **S6** (5.028g, 12.13 mmol, 75 %) as a viscous yellow syrup.

R_f (CyH/EtOAc 7/3) 0.33; **$[\alpha]_D^{24}$** +185 (c = 1.0, $CHCl_3$); **^{1}H NMR** (599 MHz, $CDCl_3$) δ 7.41 – 7.36 (m, 2H, Tol), 7.16 – 7.12 (m, 2H, Tol), 5.57 (dd, J_{FH} = 14.4 Hz and J = 1.6 Hz, 1H, H-C1), 5.38 (t, J = 10.0 Hz, 1H, H-C4), 5.22 (ddd, J_{FH} = 28.1 Hz and J = 10.0, 2.5 Hz, 1H, H-C3), 5.03 (ddd, J_{FH} = 50.0 Hz and J = 2.5, 1.7 Hz, 1H, H-C2), 4.51 (ddd, J = 10, 5.4, 2.3 Hz, 1H. H-C5), 4.29 (dd, J = 12.3, 5.4 Hz, 1H, H-C6), 4.11 (dd, J = 12.3, 2.3 Hz, 1H, H-C6), 2.33 (s, 3H, Tol), 2.10 (s, 3H, Ac), 2.07 (s, 3H, Ac), 2.06 (s, 3H, Ac) ppm; **^{13}C NMR** (151 MHz, $CDCl_3$) δ 170.7, 170.2, 169.6 (C=O, Ac), 138.8, 132.7, 130.2, 128.5 (Tol), 88.5 (d, J = 188.8 Hz, C2), 86.0 (d, J = 22.4 Hz, C1), 70.4 (d, J = 17.6 Hz, C3), 69.7 (C5), 66.1 (d, J = 1.3 Hz, C4), 62.3 (C6), 21.2 (Tol), 20.8 (3 x Ac) ppm; **^{19}F NMR** (564 MHz, $CDCl_3$) δ -189.71 (ddd, J_{FH} = 50.0, 28.1, 14.4 Hz) ppm; **IR** (neat) $\tilde{\nu}_{max}$ / cm^{-1} 2940 (w), 2333 (w), 2109 (w), 1746 (s), 1494 (m), 1436 (m), 1369 (s), 1225 (s), 1104 (s), 1062 (s), 1018 (m), 977 (m), 917 (m), 860 (m), 811 (m), 795 (m), 771 (m); **HRMS** (ESI) m/z $[M+Na]^+$ calcd for $C_{19}H_{23}FO_7SNa^+$ 437.1041; found 437.1042.

4-Methylphenyl 4,6-*O*-benzylidene-2-deoxy-2-fluoro-1-thio-α-D-mannopyranoside (S7)

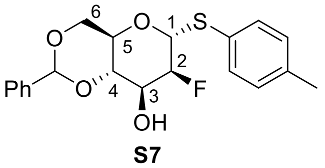

S7

NaOMe (82.0 mg, 1.52 mmol, 0.4 eq.) was added to a solution of **S6** (1.573 g, 3.795 mmol, 1.0 eq.) in MeOH (30 mL) at rt and stirred for 3 h. The yellow solution was neutralised with Amberlyst[H^+]®, concentrated *in vacuo* and the residue was co-evaporated with toluene (3 x) to remove residual methanol. The residue was dissolved in acetonitrile (38 mL) and benzaldehyde dimethyl acetal (1.14 mL, 7.59 mmol, 2.0 eq.) and *p*-toluenesulfonic acid (65.4 mg, 0.380 mmol, 0.1 eq.) were added. After stirring overnight (14 h), the solution was quenched with NEt_3 (0.2 mL), concentrated *in vacuo* and purified by column chromatography (SiO_2, CyH/EtOAc 19/1 → 9/1) to afford **S7** as a white amorphous solid (1.245 g, 3.308 mmol, 87%).

R_f (CyH/EtOAc 4/1) 0.27; **m.p.** 146 °C; **$[\alpha]_D^{23}$** +284 (c = 1.0, CH_2Cl_2); **^{1}H NMR** (599 MHz, CD_2Cl_2) δ 7.53 – 7.47 (m, 2H, benzylidene), 7.43 – 7.36 (m, 5H, benzylidene, Tol), 7.21 – 7.15 (m, 2H, Tol), 5.60 (s, 1H, C*H*Ph), 5.56 (dd, J_{FH} = 15.4 Hz and J = 1.5 Hz, 1H, H-C1), 5.05 (ddd, J_{FH} = 49.4 Hz and J = 2.7, 1.5 Hz, 1H, H-C2), 4.33 (ddd, J = 10.2, 9.4, 5.0 Hz, 1H, H-C5), 4.24 (dd, J = 10.3, 5.0 Hz, 1H, H-C6), 4.11 (dddd, J_{FH} = 27.4 Hz and J = 10.1, 5.8, 2.7 Hz, 1H, H-C3), 3.95 (td, J = 9.7 Hz and J_{FH} = 1.6 Hz, 1H, H-C4), 3.83 (t, J = 10.3 Hz, 1H, H-C6), 2.51 (d, J = 5.9 Hz, 1H, OH), 2.35 (s, 3H, Tol) ppm; **^{13}C NMR** (151 MHz, CD_2Cl_2) δ 139.4 (Tol), 137.9 (benzylidene), 133.5 (Tol), 130.6 (Tol), 129.8 (benzylidene), 129.4 (Tol), 128.8 (benzylidene), 126.8 (benzylidene), 102.8 (CHPh), 92.0 (d, J = 185.8 Hz, C2), 87.8 (d, J = 23.7 Hz, C1), 79.5 (d, J = 2.2 Hz, C4), 69.1 (d, J = 17.9 Hz, C3), 68.9 (C6), 65.3 (C5), 21.4 (Tol) ppm; **^{19}F NMR** (564 MHz, CD_2Cl_2) δ -190.33 (ddd, J_{FH} = 49.4, 27.4, 15.4 Hz) ppm; **IR** (neat) $\tilde{\nu}_{max}$ / cm^{-1} 2952 (w), 2899 (w), 2868 (w), 1713 (s), 1493 (w), 1453 (w), 1412 (w), 1382 (m), 1356 (w), 1311 (w), 1279 (m),

1267 (m), 1216 (w), 1179 (w), 1156 (w), 1099 (s), 1091 (s), 1071 (s), 1036 (s), 1019 (s), 998 (s), 977 (m), 914 (m), 876 (w), 859 (m), 811 (m), 794 (m), 774 (s), 749 (m), 698 (s), 675 (m); **HRMS** (ESI) m/z $[M+Na]^+$ calcd for $C_{20}H_{21}FO_4SNa^+$ 399.1037; found 399.1040.

4-Methylphenyl 3-*O*-benzoyl-4-*O*-benzyl-2-deoxy-2-fluoro-1-thio-α-D-mannopyranoside (S8)

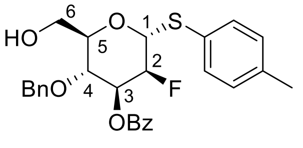

S8

A solution of **S7** (1.646 g, 4.372 mmol, 1.0 eq.), benzoyl chloride (2.03 mL, 17.5 mmol, 4.0 eq.) and pyridine (3.5 mL, 43.7 mmol, 10 eq.) in DCM (43 mL) was stirred at rt for 24 h. Upon completion of the reaction, the mixture was diluted with DCM (50 mL), quenched with water and the organic layer was washed with 2 m NaOH (2 x). The aqueous layer was extracted with DCM (2 x 30 mL) and the combined organic layers were washed with 1 m HCL (2 x), sat. aqueous $NaHCO_3$ and brine, dried over $MgSO_4$ and concentrated *in vacuo*. The off-white solid residue was dissolved in dry DCM (50 mL) and cooled to 0 °C under argon. Borane (1 M in THF, 13.1 mL, 13.1 mmol, 3.0 eq.) and TMSOTf (237 µL, 1.31 mmol, 0.3 eq.) were added successively and the mixture was stirred at 0 °C for 8.5 h. The reaction mixture was neutralised with NEt_3 (0.6 mL) and the borane was quenched by careful addition of MeOH. The mixture was concentrated *in vacuo* and purified by column chromatography (SiO_2, CyH/EtOAc 19/1 → 9/1 → 6/1) to afford benzylether **S8** (1.225 g, 2.537 mmol, 58 %) as a colourless syrup, along with 27 % of the recovered intermediate C3-benzoate benzylidene.

R_f (CyH/EtOAc 4/1) 0.29; **$[\alpha]_D$**^{21} +114 (c = 1.0, $CHCl_3$); **^{1}H NMR** (599 MHz, $CDCl_3$) δ 8.13 – 8.08 (m, 2H, Bz), 7.64 – 7.58 (m, 1H, Bz), 7.51 – 7.46 (m, 2H, Bz), 7.43 – 7.38 (m, 2H, Tol), 7.28 – 7.20 (m, 5H, Bn), 7.16 (d, J = 8.1 Hz, 2H, Tol), 5.57 (dd, J_{FH} = 14.2 Hz and J = 1.7 Hz, 1H, H-C1), 5.54 (ddd, J_{FH} = 28.7 Hz and J = 9.8, 2.4 Hz, 1H, H-C3), 5.19 (dt, J_{FH} = 50.4 Hz and J = 2.1 Hz, 1H, H-C2), 4.78 (d, J = 11.0 Hz, 1H, CH_2Ph), 4.71 (d, J = 11.0 Hz, 1H, CH_2Ph), 4.33 (dt, J = 9.8, 3.1 Hz, 1H, H-C5), 4.25 (t, J = 9.8 Hz, 1H, H-C4), 3.93 – 3.85 (m, 2H, H-C6), 2.35 (s, 3H, Tol), 1.87 (br s, 1H, OH) ppm; **^{13}C NMR** (151 MHz, $CDCl_3$) δ 165.7 (C=O, Bz), 138.7 (Tol), 137.6 (Bn), 133.6 (Bz), 133.0 (Tol), 130.2 (Tol), 130.0 (Bz), 129.6 (Bz), 129.0 (Tol), 128.7 (Bz), 128.6 (Bn), 128.2 (Bn), 128.1 (Bn), 89.2 (d, J = 186.4 Hz, C2), 86.3 (d, J = 22.2 Hz, C1), 75.4 (CH_2Ph), 73.6 (d, J = 17.6 Hz, C3), 73.3 (C5), 72.8 (d, J = 1.2 Hz, C4), 61.7 (C6), 21.3 (Tol) ppm; **^{19}F NMR** (564 MHz, $CDCl_3$) δ -189.66 (ddd, J_{FH} = 50.4, 28.7, 14.4 Hz) ppm; **IR** (neat) $\tilde{\nu}_{max}$ / cm^{-1} 2921 (w), 1721 (s), 1602 (w), 1493 (m), 1452 (m), 1352 (m), 1315 (m), 1266 (s), 1210 (w), 1178 (w), 1088 (s), 1026 (s), 912 (w), 846 8m), 796 (m), 768 (m), 735 (m), 712 (s); **HRMS** (ESI) m/z $[M+Na]^+$ calcd for $C_{27}H_{27}FO_5SNa^+$ 505.1455; found 505.1462.

4-Methylphenyl 3-*O*-benzoyl-4-*O*-benzyl-6-*O*-fluorenylmethoxycarbonyl-2-deoxy-2-fluoro-1-thio-α-D-mannopyranoside (2)

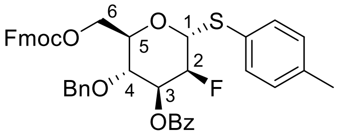

2

Pyridine (390 µL, 4.81 mmol, 3.0 eq.) was added to a solution of **S8** (773.7 mg, 1.603 mmol, 1.0 eq.), DMAP (9.8 mg, 0.08 mmol, 0.05 eq.) and Fmoc chloride (622.1 mg, 2.405 mmol, 1.5 eq.) in DCM (20 mL) at rt and the rose solution was stirred overnight (16 h). The colourless solution was diluted with DCM (35 mL), washed with 1 m HCl (2 x 15 mL) and brine, dried over $MgSO_4$ and concentrated *in vacuo*. The crude product was purified by column chromatography (SiO_2, CyH/EtOAc 19/1) to afford **2** (1.090 g, 1.546 mmol, 96 %) as a white solid foam.

R_f (CyH/EtOAc 9/1) 0.25; **m.p.** 54 °C; **$[\alpha]_D$**^{23} +56.7 (c = 1.0, $CHCl_3$); **^{1}H NMR** (599 MHz, $CDCl_3$) δ 8.15 – 8.10 (m, 2H, Bz), 7.79 (ddt, J = 7.6, 1.9, 0.9 Hz, 2H, Fmoc), 7.69 – 7.60 (m, 3H, Bz, Fmoc), 7.52 – 7.47 (m, 2H, Bz), 7.45 – 7.39 (m, 4H, Tol, Fmoc), 7.32 (tdd, J = 7.5, 3.6, 1.1 Hz, 2H, Fmoc), 7.26 – 7.20 (m, 5H, Bn), 7.15 – 7.10 (m, 2H, Tol), 5.60 (dd, J_{FH} = 14.4 Hz and J = 1.8 Hz, 1H, H-C1), 5.55 (ddd, J_{FH} = 28.5 Hz and J = 9.9, 2.7 Hz, 1H, H-C3), 5.21 (dt, J_{FH} = 50.4 Hz and J = 2.1 Hz, 1H, H-C2), 4.78 (d, J = 10.9 Hz, 1H, CH_2Ph), 4.66 (d, J = 10.9 Hz, 1H, CH_2Ph), 4.58 (ddd, J = 9.9, 5.0, 2.1 Hz, 1H, H-C5), 4.53 (dd, J = 11.7, 2.2 Hz, 1H, H-C6), 4.48 (dd, J = 11.7, 4.9 Hz, 1H, H-C6), 4.46 (dd, J = 10.4, 7.4 Hz, 1H, CH_2 Fmoc), 4.39 (dd, J = 10.4, 7.6 Hz, 1H, CH_2 Fmoc), 4.29 (t, J = 7.5 Hz, 1H, CH Fmoc), 4.22 (t, J = 9.9 Hz, 1H, H-C4), 2.31 (s, 3H, Tol) ppm; **^{13}C NMR** (151 MHz, $CDCl_3$) δ 165.7 (C=O, Bz), 155.2 (C=O, Fmoc), 143.6 (Fmoc), 143.4 (Fmoc), 141.44 (Fmoc), 141.43 (Fmoc), 138.7 (Tol), 137.3 (Bn), 133.7 (Bz), 132.9 (Tol), 130.2 (Tol), 130.0 (Bz), 129.5 (Bz), 129.0 (Tol), 128.7 (Bz), 128.6 (Bn), 128.24 (Bn), 128.21 (Bn), 128.1 (Fmoc), 128.0 (Fmoc), 127.4 (Fmoc), 127.3 (Fmoc), 125.4(Fmoc), 125.3 (Fmoc), 120.2 (2 x Fmoc), 89.1 (d, J = 187.1 Hz, C2), 86.3 (d, J = 22.4 Hz. C1), 75.4 (CH_2Ph), 73.7 (d, J = 17.6 Hz, C3), 73.1 (C4), 71.0 (C5), 70.2 (CH_2 Fmoc), 66.5 (C6), 46.9 (CH Fmoc), 21.3 Tol) ppm; **^{19}F NMR** (564 MHz, $CDCl_3$) δ -189.55 (ddd, J_{FH} = 50.3, 28.5, 14.4 Hz) ppm; **IR** (neat) $\tilde{\nu}_{max}$ / cm^{-1} 2952 (w), 1723 (s), 1601 (w), 1493 (w), 1451 (m), 1395 (w), 1316 (w), 1252 (s), 1178 (m), 1098 (s), 1027 (m), 969 (m), 917 (w), 849 (w), 786 (m), 740 (s), 712 (s); **HRMS** (ESI) m/z $[M+Na]^+$ calcd for $C_{42}H_{37}FO_7SNa^+$ 727.2136; found 727.2137.

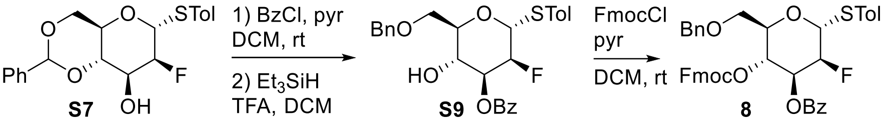

4-Methylphenyl 3-*O*-benzoyl-6-*O*-benzyl-2-deoxy-2-fluoro-1-thio-α-D-mannopyranoside (S9)

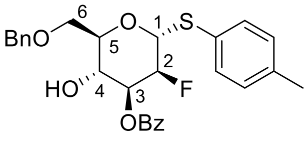

S9

The recovered intermediate 3-*O*-benzoate (560.7 mg, 1.167 mmol, 1.0 eq.) from the synthesis of **S8** was dissolved in dry DCM (10 mL) and cooled to 0 °C under argon. Triethyl silane (0.93 mL, 5.8 mmol, 5.0 eq.) and trifluoroacetic acid (0.45 mL, 5.8 mmol, 5.0 eq.) were added successively and the mixture was stirred at 0 °C for 0.5 h. The reaction mixture was allowed to warm to rt and stirred at rt until TLC indicated full conversion (4 h). The mixture was diluted with DCM, washed with sat. aqueous $NaHCO_3$ and the aqueous layer was extracted with DCM. The combined organic layers were washed with brine, dried over $MgSO_4$ and concentrated *in vacuo*. The crude product was purified by column chromatography (SiO_2, CyH/EtOAc 19/1 → 9/1) to afford benzylether **S9** (374.2 mg, 0.775mmol, 66 %) as a crystalline white solid.

R_f (CyH/EtOAc 4/1) 0.29; **m.p.** 108 °C; **$[\alpha]_D^{24}$** +134 (c = 1.0, $CHCl_3$); **^{1}H NMR** (599 MHz, $CDCl_3$) δ 8.16 – 8.09 (m, 2H, Bz), 7.63 – 7.58 (m, 1H, Bz), 7.51 – 7.44 (m, 2H, Bz), 7.43 – 7.39 (m, 2H, Tol), 7.38 – 7.33 (m, 4H, Bn), 7.33 – 7.28 (m, 1H, Bn), 7.14 – 7.05 (m, 2H, Tol), 5.60 (dd, J_{FH} = 14.4 Hz and J = 1.5 Hz, 1H, H-C1), 5.36 (ddd, J_{FH} = 28.5 Hz and J = 9.8, 2.5 Hz, 1H, H-C3), 5.14 (dt, J_{FH} = 50.3 Hz and J = 1.8 Hz, 1H, H-C2), 4.65 (d, J = 11.9 Hz, 1H, C*H*₂Ph), 4.57 (d, J = 11.8 Hz, 1H, C*H*₂Ph), 4.44 (dt, J = 9.3, 4.5 Hz, 1H, H-C5), 4.29 (t, J = 9.7 Hz, 1H, H-C4), 3.88 (dd, J = 10.5, 4.9 Hz, 1H, H-C6), 3.84 (dd, J = 10.5, 4.2 Hz, 1H, H-C6), 2.75 (s, 1H, HO-C4), 2.33 (s, 3H, Tol) ppm; **^{13}C NMR** (151 MHz, $CDCl_3$) δ 166.6 (C=O, Bz), 138.5 (Tol), 137.9 (Bn), 133.7 (Bz), 132.8 (Tol), 130.16 (Bz), 130.15 (Tol), 129.5 (Bz), 129.2 (Tol), 128.64 (Bz), 128.59 (Bn), 128.0 (Bn), 127.9 (Bn), 88.9 (d, J = 187.6 Hz, C2), 86.4 (d, J = 22.3 Hz, C1), 73.9 (CH_2Ph), 73.5 (d, J = 17.3 Hz, C3), 72.3 (C5), 70.1 (C6), 67.7 (d, J = 1.4 Hz, C4), 21.3 (Tol) ppm; **^{19}F NMR** (564 MHz, $CDCl_3$) δ -188.90 (ddd, J_{FH} = 50.3, 28.5, 14.4 Hz) ppm; **IR** (neat) $\tilde{\nu}_{max}$ / cm^{-1} 3477 (w), 2923 (m), 1722 (s), 1602 (m), 1493 (m), 1452 (m), 1351 (m), 1316 (m), 1278 (s), 1179 (m), 1097 (s), 1027 (m), 855 (m), 808 (m), 771 (m), 737 (m), 713 (s); **HRMS** (ESI) m/z $[M+Na]^+$ calcd for $C_{27}H_{27}FO_5SNa^+$ 505.1455; found 505.1416.

4-Methylphenyl 3-*O*-benzoyl-6-*O*-benzyl-4-*O*-fluorenylmethoxycarbonyl-2-deoxy-2-fluoro-1-thio-α-D-mannopyranoside (8)

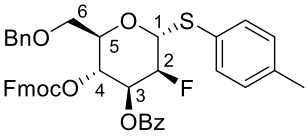

8

Pyridine (240 µL, 2.98 mmol, 4.0 eq.) was added to a solution of **S9** (359 mg, 0.744 mmol, 1.0 eq.) and Fmoc chloride (250.2 mg, 0.967 mmol, 1.3 eq.) in DCM (7.5 mL) at rt and the yellow solution was stirred overnight (20 h). The colourless solution was diluted with DCM (40 mL), washed with 1 m HCl (2 x 15 mL) and brine, dried over $MgSO_4$ and concentrated *in vacuo*. The crude product was purified by column chromatography (SiO_2, CyH/EtOAc 19/1) to afford **8** (500 mg, 0.709 mmol, 95 %) as a white solid foam.

R_f (CyH/EtOAc 9/1) 0.25; **m.p.** 112 °C; **$[\alpha]_D^{24}$** +86.5 (c = 1.0, $CHCl_3$); **^{1}H NMR** (599 MHz, $CDCl_3$) δ 8.07 – 8.02 (m, 2H, Bz), 7.72 (tt, J = 7.5, 0.9 Hz, 2H, Fmoc), 7.53 – 7.49 (m, 1H, Bz), 7.47 – 7.43 (m, 4H, Tol, Fmoc), 7.39 – 7.34 (m, 4H, Bz, Fmoc), 7.34 – 7.32 (m, 2H, Bn), 7.31 – 7.28 (m, 2H, Bn), 7.25 – 7.22 (m, 1H, Bn), 7.21 (td, J = 7.4, 1.1 Hz, 1H, Fmoc), 7.17 (td, J = 7.5, 1.1 Hz, 1H, Fmoc), 7.11 – 7.08 (m, 2H, Tol), 5.64 (dd, J_{FH} = 14.2 Hz and J = 1.7 Hz, 1H, H-C1), 5.57 – 5.49 (m, 2H, H-C3, H-C4), 5.25 (dt, J_{FH} = 50.1 Hz and J = 2.0 Hz, 1H, H-C2), 4.70 (ddd, J = 8.6, 5.1, 2.8 Hz, 1H, H-C5), 4.61 (d, J = 11.9 Hz, 1H, C*H*₂Ph), 4.51 (d, J = 11.9 Hz, 1H, C*H*₂Ph), 4.32 (dd, J = 10.5, 7.3 Hz, 1H, C*H*₂ Fmoc), 4.22 (dd, J = 10.5, 7.7 Hz, 1H, C*H*₂ Fmoc), 4.03 (t, J = 7.5 Hz, 1H, C*H* Fmoc), 3.76 (dd, J = 11.0, 5.2 Hz, 1H, H-C6), 3.72 (dd, J = 11.0, 2.9 Hz, 1H, H-C6), 2.33 (s, 3H, Tol) ppm; **^{13}C NMR** (151 MHz, $CDCl_3$) δ 165.8 (Bz), 154.4 (Fmoc), 143.3 (Fmoc), 143.1 (Fmoc), 141.32 (Fmoc), 141.29 (Fmoc), 138.7 (Tol), 138.0 (Bn), 133.7 (Bz), 133.0 (Tol), 130.2 (Tol), 130.1 (Bz), 129.1 (Bz), 128.8 (Tol), 128.6 (Bz), 128.4 (Bn), 128.0 (Fmoc), 127.9 (Bn), 127.7 (Bn), 127.3 (Fmoc), 125.2 (Fmoc), 125.1 (Fmoc), 120.11 (Fmoc), 120.09 (Fmoc), 88.7 (d, J = 188.6 Hz, C2), 86.2 (d, J = 22.0 Hz, C1), 73.7 (CH_2Ph), 71.5 (d, J = 17.7 Hz, C3), 70.9 (d, J = 1.1 Hz, C4), 70.8 (C5), 70.4 (CH_2 Fmoc), 68.9 (C6), 46.7 (CH Fmoc), 21.3 (Tol) ppm; **^{19}F NMR** (564 MHz, $CDCl_3$) δ -189.92 – -190.12 (m) ppm; **IR** (neat) $\tilde{\nu}_{max}$ / cm^{-1} 2868 (w), 1755 (s), 1726 (s), 1602 (w), 1493 (m), 1451 (m), 1386 (m), 1272 (s), 1249 (s), 1179 (m), 1101 (s), 1027 (m), 992 (s), 909 (w), 857 (w), 806 (m), 782 (m), 760 (m), 742 (s), 713 (s); **HRMS** (ESI) m/z $[M+Na]^+$ calcd for $C_{42}H_{37}FO_7SNa^+$ 727.2136; found 727.2137.

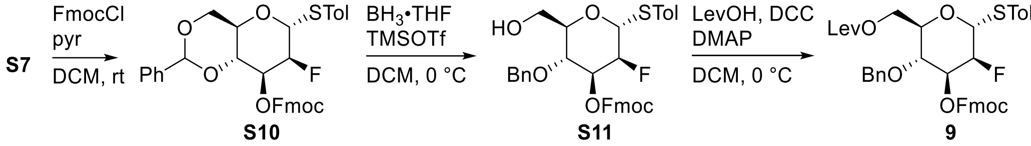

4-Methylphenyl 4,6-*O*-benzylidene-3-*O*-fluorenylmethoxycarbonyl-2-deoxy-2-fluoro-1-thio-α-D-mannopyranoside (S10)

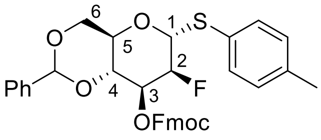

S10

Pyridine (2.3 mL, 28 mmol, 4.0 eq.) was added to a solution of benzylidene **S7** (2.653 g, 7.048 mmol, 1.0 eq.) and Fmoc chloride (2.735 g, 10.57 mmol, 1.5 eq.) in DCM (70 mL) at rt and the yellow solution was stirred overnight (16 h). The colourless solution was diluted with DCM (150 mL), washed with 0.5 M HCl (50 mL), sat. aqueous $NaHCO_3$ and brine, dried over $MgSO_4$ and concentrated *in vacuo*. The crude product was purified by column chromatography (SiO_2, CyH → CyH/EtOAc 19/1) to afford **S10** (4.094 g, 6.838 mmol, 97 %) as a white solid foam.

R_f (CyH/EtOAc 9/1) 0.30; **m.p.** 66 °C; **$[\alpha]_D^{25}$** +90.2 (c = 2.0, CH_2Cl_2); **^{1}H NMR** (599 MHz, CD_2Cl_2) δ 7.79 (d, *J* = 7.6 Hz, 2H, Fmoc), 7.62 (d, *J* = 7.5 Hz, 1H, Fmoc), 7.60 (d, *J* = 7.5 Hz, 1H, Fmoc), 7.51 – 7.46 (m, 2H, benzylidene), 7.45 – 7.39 (m, 4H, Fmoc, Tol), 7.39 – 7.36 (m, 3H benzylidene), 7.31 (tt, *J* = 7.5, 1.0 Hz, 1H, Fmoc), 7.27 (tt, *J* = 7.5, 1.0 Hz, 1H, Fmoc), 7.19 (d, *J* = 7.9 Hz, 2H, Tol), 5.62 (s, 1H, C*H*Ph), 5.59 (br d, J_{FH} = 15.7 Hz, 1H, H-C1), 5.19 (dt, J_{FH} = 49.7 Hz and *J* = 2.1 Hz, 1H, H-C2), 5.16 (ddd, J_{FH} = 26.2 Hz and *J* = 10.4, 2.6 Hz, 1H, H-C3), 4.53 (dd, *J* = 10.6, 7.0 Hz, 1H, C*H*2 Fmoc), 4.49 (dd, *J* = 10.7, 7.1 Hz, 1H, C*H*2 Fmoc), 4.45 (td, *J* = 9.8, 4.8 Hz, 1H, H-C5), 4.29 (t, *J* = 7.0 Hz, 1H, C*H* Fmoc), 4.27 (dd, *J* = 10.4, 4.7 Hz, 1H, H-C6), 4.22 (t, *J* = 10.0 Hz, 1H, H-C4), 3.88 (t, *J* = 10.3 Hz, 1H, H-C6), 2.36 (s, 3H, Tol) ppm; **^{13}C NMR** (151 MHz, CD_2Cl_2) δ 154.6 (C=O, Fmoc), 143.8 (Fmoc), 143.6 (Fmoc), 141.69 (Fmoc), 141.68 (Fmoc), 139.4 (Tol), 137.6 (benzylidene), 133.4 (Tol), 130.5 (Tol), 129.6 (benzylidene), 128.8 (Tol), 128.6 (benzylidene), 128.3 (Fmoc), 127.60 (Fmoc), 127.59 (Fmoc), 126.7 (benzylidene), 125.44 (Fmoc), 125.39 (Fmoc), 120.5 (Fmoc), 120.4 (Fmoc), 102.4 (CHPh), 89.4 (d, *J* = 188.3 Hz, C2), 87.4 (d, *J* = 23.1 Hz, C1), 76.2 (d, *J* = 2.0 Hz, C4), 73.5 (d, *J* = 17.1 Hz, C3), 70.6 (CH_2 Fmoc), 68.7 (C6), 65.6 (C5), 47.1 (CH Fmoc), 21.3 (Tol) ppm; **^{19}F NMR** (564 MHz, CD_2Cl_2) δ -188.55 (ddd, J_{FH} = 48.6, 26.2, 15.1 Hz) ppm; **IR** (neat) $\tilde{\nu}_{max}$ / cm^{-1} 2922 (w), 1749 (s), 1492 (w), 1478 (w), 1451 (m), 1385 (m), 1327 (w), 1272 (s), 1253 (s), 1209 (m), 1100 (s), 986 (m), 970 (s), 912 (w), 867 (w), 811 (m), 796 (m), 759 (s), 738 (s), 699 (s), 667 (w); **HRMS** (ESI) m/z $[M+K]^+$ calcd for $C_{35}H_{31}FO_6SK^+$ 637.1457; found 637.1460.

4-Methylphenyl 4-*O*-benzyl-3-*O*-fluorenylmethoxycarbonyl-2-deoxy-2-fluoro-1-thio-α-D-mannopyranoside (S11)

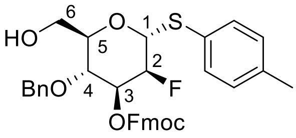

S11

Borane (1 M in THF, 3.7 mL, 3.7 mmol, 3.0 eq.) and TMSOTf (67.6 µL, 0.374 mmol, 0.3 eq.) were added successively to a solution of benzylidene **S10** (745.5 mg, 1.245 mmol, 1.0 eq.) in dry DCM (12.5 mL) at 0 °C under argon. The solution was stirred at 0 °C for 7.5 h and quenched by careful addition of water. The layers were separated and the aqueous layers was extracted with DCM (3 x 30 mL). The combined organic layers were washed with brine, dried over $MgSO_4$ and concentrated *in vacuo*. The crude product was purified by column chromatography (SiO_2, CyH/EtOAc 9/1) to afford **S11** (596.4 mg, 0.9928 mmol, 80 %) as a white solid foam.

R_f (CyH/EtOAc 4/1) 0.28; **m.p.** 48 °C; **$[\alpha]_D^{25}$** +58.8 (c = 1.0, $CHCl_3$); **^{1}H NMR** (599 MHz, $CDCl_3$) δ 7.78 (dd, *J* = 7.6, 2.7 Hz, 2H, Fmoc), 7.65 – 7.62 (m, 1H, Fmoc), 7.62 – 7.60 (m, 1H, Fmoc), 7.41 (t, *J* = 7.5 Hz, 2H, Fmoc), 7.39 – 7.36 (m, 2H, Tol), 7.35 – 7.27 (m, 7H, Bn, Fmoc), 7.15 (d, *J* = 7.9 Hz, 2H, Tol), 5.55 (dd, J_{FH} = 14.4 Hz and *J* = 1.6 Hz, 1H, H-C1), 5.15 (dt, J_{FH} = 50.1 Hz and *J* = 2.1 Hz, 1H, H-C2), 5.12 (ddd, J_{FH} = 28.4 Hz and *J* = 9.8, 2.5 Hz, 1H, H-C3), 4.83 (d, *J* = 11.1 Hz, 1H, C*H*2Ph), 4.70 (d, *J* = 11.2 Hz, 1H, C*H*2Ph), 4.48 (dd, *J* = 10.5, 7.5 Hz, 1H, C*H*2 Fmoc), 4.44 (dd, *J* = 10.5, 7.4 Hz, 1H, C*H*2 Fmoc), 4.28 (t, *J* = 7.5 Hz, 1H, C*H* Fmoc), 4.25 (dt, *J* = 9.8, 3.3 Hz, 1H, H-C5), 4.12 (t, *J* = 9.8 Hz, 1H, H-C4), 3.89 – 3.80 (m, 2H, H-C6), 2.35 (s, 3H, Tol), 1.76 (t, *J* = 6.7 Hz, 1H, OH) ppm; **^{13}C NMR** (151 MHz, $CDCl_3$) δ 154.5 (C=O, Fmoc), 143.4 (Fmoc), 143.3 (Fmoc), 141.47 (Fmoc), 141.45 (Fmoc), 138.8 (Tol), 137.7 (Bn), 133.1 (Tol), 130.3 (Tol), 128.8 (Bn), 128.2 (Bn), 128.11 (Fmoc), 128.10 (Bn), 127.4 (Fmoc), 125.3 (Fmoc), 125.2 (Fmoc), 120.3 (Fmoc), 88.8 (d, *J* = 187.0 Hz, C2), 86.2 (d, *J* = 22.1 Hz, C1), 77.1 (d, *J* = 17.9 Hz, C3), 75.5 (CH_2Ph), 73.2 (C5), 72.6 (C4), 70.5 (CH_2 Fmoc), 61.7 (C6), 46.8 (CH Fmoc), 21.3 (Tol) ppm; **^{19}F NMR** (564 MHz, $CDCl_3$) δ -189.97 (ddd, J_{FH} = 49.9, 28.1, 14.4 Hz) ppm; **IR** (neat) $\tilde{\nu}_{max}$ / cm^{-1} 2923 (w), 1750 (m), 1493 (w), 1451 (m), 1389 (w), 1358 (w), 1260 (s), 1153 (w), 1100 (m), 1088 (s), 968 (w), 886 (w), 847 (w), 800 (w), 760 (m), 739 (s), 699 (m); **HRMS** (ESI) m/z $[M+Na]^+$ calcd for $C_{35}H_{33}FO_6SNa^+$ 623.1874; found 623.1877.

4-Methylphenyl 4-*O*-benzyl-3-*O*-fluorenylmethoxycarbonyl-2-deoxy-2-fluoro-6-*O*-levulinoyl-1-thio-α-D-mannopyranoside (9)

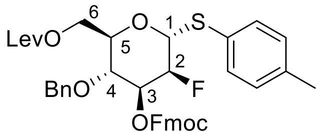

9

DCC (1.245 g, 6.034 mmol, 2.3 eq.), DMAP (96.2 mg, 0.787 mmol, 0.3 eq.) and levulinic acid (618 µL, 6.03 mmol, 2.3 eq.) were successively added to a solution of **S11** (1.576 g, 2.624 mmol, 1.0 eq.) in dry DCM (27 mL) at 0 °C under argon and the solution was stirred for 0.5 h at 0 °C. The resulting white suspension was filtered over Celite®, washed with DCM and the collected filtrate was washed with 1 m HCl, and the aqueous layer was extracted with DCM (30 mL). The combined organic layers were washed with brined, dried over $MgSO_4$, concentrated *in vacuo* and purified by column chromatography (SiO_2, CyH/EtOAc 8/1 → 4/1) to afford levulinoyl ester **9** (1.669 g, 2.389 mmol, 91 %) as a white solid foam.

R$_f$ (CyH/EtOAc 4/1) 0.25; **m.p.** 54 °C; **[α]$_D^{25}$** +128.7 (c = 1.0, $CHCl_3$); **^{1}H NMR** (500 MHz, $CDCl_3$) δ 7.79 – 7.75 (m, 2H, Fmoc), 7.64 – 7.59 (m, 2H, Fmoc), 7.41 (td, *J* = 7.5, 1.1 Hz, 2H, Fmoc), 7.38 – 7.35 (m, 2H, Tol), 7.34 – 7.26 (m, 7H, Bn, Fmoc), 7.16 – 7.11 (m, 2H, Tol), 5.55 (dd, J_{FH} = 14.4 Hz and *J* = 1.8 Hz, 1H, H-C1), 5.14 (dt, J_{FH} = 50.4 Hz and *J* = 2.1 Hz, 1H, H-C2), 5.09 (ddd, J_{FH} = 28.3 Hz and *J* = 9.6, 2.4 Hz, 1H, H-C3), 4.81 (d, *J* = 11.1 Hz, 1H, C*H*$_2$Ph), 4.63 (d, *J* = 11.0 Hz, 1H, C*H*$_2$Ph), 4.49 (dd, *J* = 10.5, 7.4 Hz, 1H, C*H*$_2$ Fmoc), 4.44 (dd, *J* = 10.5, 7.3 Hz, 1H, C*H*$_2$ Fmoc), 4.44 – 4.41 (m, 1H, H-C5), 4.39 (dd, *J* = 11.7, 4.7 Hz, 1H, H-C6), 4.32 (dd, *J* = 11.7, 1.9 Hz, 1H, H-C6), 4.28 (t, *J* = 7.4 Hz, 1H, C*H* Fmoc), 4.01 (t, *J* = 9.6 Hz, 1H, H-C4), 2.80 – 2.66 (m, 2H, Lev), 2.59 (t, *J* = 6.6 Hz, 2H, Lev), 2.34 (s, 3H, Tol), 2.19 (s, 3H, Lev) ppm; **^{13}C NMR** (126 MHz, $CDCl_3$) δ 206.5 (C=O, Lev), 172.5 (C=O, Lev), 154.4 (C=O, Fmoc), 143.3 (Fmoc), 143.2 (Fmoc), 141.48 (Fmoc), 141.46 (Fmoc), 138.7 (Tol), 137.5 (Bn), 132.8 (Tol), 130.2 (Tol), 128.8 (Tol), 128.7 (Bn), 128.24 (Bn), 128.19 (Bn), 128.13 (Fmoc), 128.12 (Fmoc), 127.4 (Fmoc), 125.3 (Fmoc), 125.2 (Fmoc), 120.3 (Fmoc), 88.6 (d, *J* = 187.5 Hz, C2), 86.0 (d, *J* = 22.1 Hz, C1), 77.3 (d, *J* = 18.0 Hz, C3), 75.4 (CH_2Ph), 72.7 (C4), 70.9 (C5), 70.5 (CH_2 Fmoc), 63.0 (C6), 46.8 (CH Fmoc), 38.0 (Lev), 30.0 (Lev), 28.0 (Lev), 21.3 (Tol) ppm; **^{19}F NMR** (470 MHz, $CDCl_3$) δ -190.02 (ddd, J_{FH} = 50.3, 28.3, 14.3 Hz) ppm; **IR** (neat) $\tilde{v}_{max}$ / cm^{-1} 3032 (w), 2950 (w), 1745 (s), 1718 (m), 1494 (w), 1451 (m), 1389 (w), 1359 (m), 1260 (s), 1208 (m), 1182 (m), 1159 (m), 1101 (s), 1088 (s), 970 (m), 887 (w), 854 (w), 811 (m), 798 (m), 760 (m), 740 (s), 699 (m); **HRMS** (ESI) m/z $[M+Na]^+$ calcd for $C_{40}H_{39}FO_8SNa^+$ 721.2242; found 721.2243.

Evaluation of the Building Blocks in Solution

General procedure for glycosylations with MeOH

To a suspension of the donor (1.0 eq., 0.5 to 0.8 mmol) and freshly activated 4 Å MS (~1 mg per mg donor) in dry DCM (2 mL) were added NIS (1.5 eq.), MeOH (10 eq.) and TfOH (0.5 eq.) successively at 0 °C. The mixture was sonicated for 1 h at 0 °C. The reaction was quenched with solid $Na_2S_2O_3$, diluted with DCM and filtered. The filtrate was washed with sat. aqueous $NaHCO_3$ and the organic layer was dried over $MgSO_4$ and concentrated *in vacuo*. The crude reaction mixture was analysed by ^{1}H and ^{19}F NMR spectroscopy to determine the reaction outcome. All ^{19}F-NMR spectra were manually phase corrected and baseline corrected with the ablative method (points: 5; passes: 10) using the MestReNova software package (version 12.0.2-20910). All resonances in the region between -150 ppm and -230 ppm were integrated for the analysis. For isolated products, the products were purified by column chromatography (SiO_2, CyH/EtOAc 19/1 → 9/1). Selected conditions were also tested with *i*PrOH instead of MeOH giving similar results.

A selection of the conditions tested to achieve full conversion of the thiodonor **2** are listed in Table S1. None of the conditions tested lead to full conversion in a single glycosylation, not even when the reaction mixture was quenched with 100 eq. MeOH prior to adding $Na_2S_2O_3$.

Table S1. Optimisation of glycosylation of donor **2** with MeOH as acceptor in solution.

entry	deviation from general procedure	**2**/**4a**/**4b**/other[a]
1	TfOH (0.1 eq.) added as stock solution; stirred overnight	100/0/0/0
2	TfOH (0.1 eq.) added as stock solution (freshly prepared from a new bottle of TfOH); NIS not recrystallised; stirred overnight	88/4/0/8
3	NIS not recrystallised; stirred overnight	61/33/0/6
4	NIS not recrystallised; stirred overnight; MeOH added in two portions	47/44/0/9
5	MeOH added in two portions; NIS not recrystallised	44/52/0/4

6	MeOH added as two portions	43/56/0/1
7	0.25 eq. TfOH; NIS added in two portions (1.0 eq. + 0.5 eq. after 30 min)	63/36/0/1
8	30 min sonication	68/32/0/0
9	5.0 eq. NIS.; MeOH added in two portions	0/25/0/75
10	2.5 eq. NIS; MeOH added in two portions	17/77/0/6
11	1,4-dioxane as solvent; NIS not recrystallised; rt to 55 °C; 4 h sonication;	27/60/6/7
12	0.25 TfOH used	61/39/0/0
13	1.0 eq. TfOH, NIS not recrystallised	59/36/1/5
14	0.2 eq. TMSOTf used as promoter; NIS not recrystallised	63/36/0/1

[a] determined by $^{19}F\{^1H\}$ NMR.

Using a high excess of NIS lead to the formation of side products, mainly by bisiodination of the Fmoc PG (for all carbohydrate species in the mixture). The use of recrystallized NIS had a slight benefit for the reaction outcome, with less unidentified side products formed during the reaction.

Glycosylation of di-benzylated donor **1** to methanol resulted in the formation of a 3:4 mixture of **3a**:**3b**, along with unreacted donor (23%) and small amounts of the alpha (5%) and beta (1%) lactols, as can be seen from the following crude $^{19}F\{^1H\}$ NMR spectrum (376 MHz, $CDCl_3$).

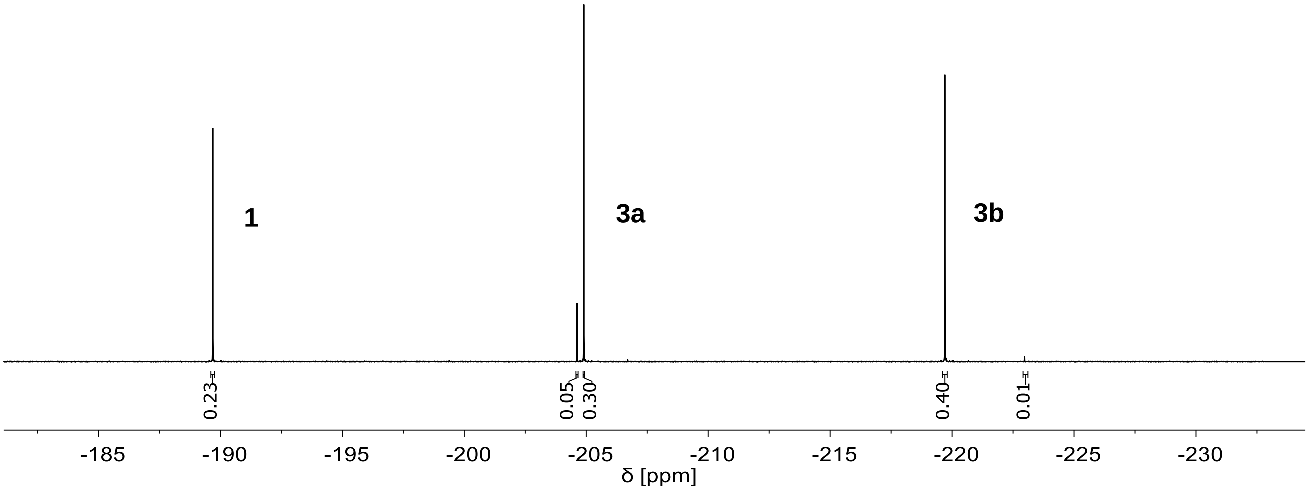

Methyl 3-*O*-benzoyl-4-*O*-benzyl-6-*O*-fluorenylmethoxycarbonyl-2-deoxy-2-fluoro-α-D-mannopyranoside (4a)

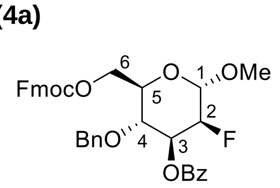

4a

R_f (CyH/EtOAc 4/1) 0.45; **$[\alpha]_D^{22}$** +21.5 (c = 1.0, $CHCl_3$); **^{1}H NMR** (599 MHz, $CDCl_3$) δ 8.11 (dd, *J* = 8.4, 1.3 Hz, 2H, Bz), 7.78 (dt, *J*= 7.6, 1.0 Hz, 2H, Fmoc), 7.65 (ddd, *J* = 11.1, 7.5, 1.0 Hz, 2H, Fmoc), 7.63 – 7.59 (m, 1H, Bz), 7.50 – 7.46 (m, 2H, Bz), 7.45 – 7.39 (m, 2H, Fmoc), 7.33 (tdd, *J* = 7.6, 3.2, 1.1 Hz, 2H, Fmoc), 7.24 – 7.18 (m, 5H, Bn), 5.56 (ddd, J_{FH} = 29.2 Hz and *J* = 9.6, 2.6 Hz, 1H, H-C3), 4.93 (dd, J_{FH} = 6.9 Hz and *J* = 1.9 Hz, 1H, H-C1), 4.90 (dt, J_{FH} = 50.3 Hz and *J* = 2.2 Hz, 1H, H-C2), 4.74 (d, *J* = 10.9 Hz, 1H, C*H*$_2$Ph), 4.63 (d, *J*= 10.9 Hz, 1H, C*H*$_2$Ph), 4.55 (dd, *J* = 11.7, 2.2 Hz, 1H, H-C6), 4.47 (dd, *J* = 10.5, 7.4 Hz, 1H, C*H*$_2$ Fmoc), 4.45 (dd, *J* = 11.6, 4.7 Hz, 1H, H-C6), 4.40 (dd, *J* = 10.4, 7.6 Hz, 1H, C*H*$_2$ Fmoc), 4.30 (t, *J* = 7.5 Hz, 1H, C*H* Fmoc), 4.15 (t, *J* = 9.8 Hz, 1H, H-C4), 4.01 (ddd, *J* = 9.9, 4.8, 2.2 Hz, 1H, H-C5), 3.46 (s, 3H, OMe) ppm; **^{13}C NMR** (151 MHz, $CDCl_3$) δ 165.7 (C=O, Bz), 155.2 (C=O, Fmoc), 143.6 (Fmoc), 143.4 (Fmoc), 141.5 (Fmoc), 141.4 (Fmoc), 137.4 (Bn), 133.6 (Bz), 130.0 (Bz), 129.7 (Bz), 128.7 (Bz), 128.6 (Bn), 128.2 (Bn), 128.1(Bn), 128.04 (Fmoc), 128.03 (Fmoc), 127.33 (Fmoc), 127.32 (Fmoc), 125.4 (Fmoc), 125.3 (Fmoc), 120.2 (Fmoc), 98.4 (d, *J* = 28.8 Hz, C1), 87.6 (d, *J* = 177.5 Hz, C2), 75.3 (CH_2Ph), 73.4 (d, *J* = 16.7 Hz, C3), 72.9 (C4), 70.2 (CH_2 Fmoc), 69.9 (C5), 66.5 (C6), 55.5 (OMe), 46.9 (CH Fmoc) ppm; **^{19}F NMR** (564 MHz, $CDCl_3$) δ -204.90 (ddd, J_{FH} = 50.3, 29.2, 6.9 Hz) ppm; **IR** (neat) $\tilde{\nu}_{max}$ / cm^{-1} 2933 (w), 1747 (s), 1720 (s), 1602 (w), 1451 (m), 1393 (m), 1315 (m), 1250 (s), 1177 (m), 1070 (s), 971 (s), 915 (m), 849 (m), 786 8(m), 758 (s), 740 (s), 712 (s); **HRMS** (ESI) m/z $[M+Na]^+$ calcd for $C_{36}H_{33}FO_8Na^+$ 635.2052; found 635.2043.

3-*O*-benzoyl-4-*O*-benzyl-6-*O*-fluorenylmethoxycarbonyl-2-deoxy-2-fluoro-α-D-mannopyranoside-(1→6)-1,2,3,4-tetra-*O*-methyl-α-D-mannopyranoside (7)

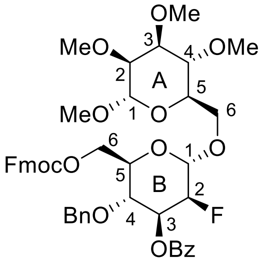

7

Donor **2** (62.2 mg, 96.8 µmol, 1.55 eq.) and 1,2,3,4-tetra-*O*-methyl-α-D-mannopyranoside **5**[3] (14.8 mg, 62.6 µmol, 1.0 eq.) were co-evaporated twice with toluene and dried *in vacuo*. Freshly activated 4 Å MS (80 mg) and dry DCM (2.5 mL) were added, and the mixture was cooled to 0 °C. NIS (31.7 mg, 0.141 mmol, 2.25 eq.) and TfOH (4.14 µL, 47.0 µmol, 0.75 eq.) were successively added at 0 °C under argon, and the dark purple mixture was stirred at 0 °C for 1 h. The reaction was quenched by addition of solid $NaHCO_3$ and solid $Na_2S_2O_3$ and was allowed to warm to rt. The colourless suspension was filtered, concentrated *in vacuo* and purified by column chromatography (SiO_2, CyH/EtOAc 4/1) to afford disaccharide **7** (50.8 mg, 62.2 µmol, 99 %) as a white foam.

R$_f$ (CyH/EtOAc 2/1) 0.30; **[α]$_D^{23}$** +23.1 (c = 1.0, $CHCl_3$); **^{1}H NMR** (500 MHz, $CDCl_3$) δ 8.13 – 8.08 (m, 2H, Bz), 7.78 (ddt, *J* = 7.5, 1.8, 0.9 Hz, 2H, Fmoc), 7.67 (dq, *J* = 7.5, 0.9 Hz, 1H, Fmoc), 7.65 (dq, *J* = 7.5, 1.0 Hz, 1H, Fmoc), 7.64 – 7.56 (m, 1H, Bz), 7.51 – 7.44 (m, 2H, Bz), 7.42 (tdd, *J* = 7.5, 1.9, 1.1 Hz, 2H, Fmoc), 7.33 (tdd, *J* = 7.5, 2.7, 1.2 Hz, 2H, Fmoc), 7.25 – 7.15 (m, 5H, Bn), 5.61 (ddd, J_{FH} = 29.3 Hz and *J* = 9.2, 2.6 Hz, 1H, H-C3B), 5.23 (dd, J_{FH} = 7.0 Hz and *J* = 1.9 Hz, 1H, H-C1B), 4.96 (dt, J_{FH} = 50.2 Hz and *J* = 2.2 Hz, 1H, H-C2B), 4.75 (d, *J* = 1.8 Hz, 1H, H-C1A), 4.72 (d, *J* = 10.9 Hz, 1H, C*H*$_2$Ph), 4.62 (d, *J* = 10.9 Hz, 1H, C*H*$_2$Ph), 4.57 (dd, *J* = 11.7, 2.0 Hz, 1H, H-C6B), 4.47 (dd, *J* = 10.3, 7.5 Hz, 1H, C*H*$_2$ Fmoc), 4.46 (dd, *J* = 11.6, 3.8 Hz, 1H, H-C6B), 4.39 (dd, *J* = 10.4, 7.6 Hz, 1H, C*H*$_2$ Fmoc), 4.31 (t, *J* = 7.5 Hz, 1H, C*H* Fmoc), 4.18 (t, *J* = 9.6 Hz, 1H, H-C4B), 4.14 (ddd, *J* = 9.9, 3.6, 2.0 Hz, 1H, H-C5B), 3.94 (dd, *J* = 11.2, 5.6 Hz, 1H, H-C6A), 3.82 (dd, *J* = 11.2, 1.8 Hz, 1H, H-C6A), 3.61 (ddd, *J* = 9.8, 5.7, 1.7 Hz, 1H. H-C5A), 3.57 – 3.55 (m, 1H, H-C2A), 3.55 (s, 3H, OMe), 3.52 (dd, *J* = 9.3, 3.2 Hz, 1H, H-C3A), 3.49 (s, 3H, OMe), 3.46 (s, 3H, OMe), 3.43 (t, *J* = 9.5 Hz, 1H, H-C4A), 3.38 (s, 3H, OMe) ppm; **^{13}C NMR** (126 MHz, $CDCl_3$) δ 165.5 (C=O, Bz), 155.2 (C=O, Fmoc), 143.6 (Fmoc), 143.4 (Fmoc), 141.43 (Fmoc), 141.42 (Fmoc), 137.5 (Bn), 133.5 (Bz), 130.0 (Bz), 129.8 (Bz), 128.7 (Bz), 128.5 (Bn), 128.2 (Bn), 128.1 (Bn), 128.02 (Fmoc), 128.01 (Fmoc), 127.34 (Fmoc), 127.33 (Fmoc), 125.5 (Fmoc), 125.4 (Fmoc), 120.2 (Fmoc), 98.0 (C1A), 97.5 (d, *J* = 29.0 Hz, C1B), 87.8 (d, *J* = 177.7 Hz, C2B), 81.5 (C3A), 77.1 (C4A), 76.4 (C2A), 75.3 (CH_2Ph), 73.2 (d, *J* = 16.9 Hz, C3B), 72.9 (C4B), 71.5 (C5A), 70.2 (CH_2 Fmoc), 70.0 (C5B), 66.9 (C6A), 66.4 (C6B), 61.0 (OMe), 59.0 (OMe), 57.7 (OMe), 55.1 (OMe), 46.9 (CH Fmoc) ppm; **^{19}F NMR** (470 MHz, $CDCl_3$) δ -204.38 (ddd, J_{FH} = 50.2, 29.2, 7.0 Hz) ppm; **IR** (neat) $\tilde{\nu}_{max}$ / cm^{-1} 2929 (w), 1748 (m), 1726 (m), 1602 (w), 1451 (m), 1392 (w), 1315 (w), 1253 (s), 1192 (m), 1095 (s), 1062 (s), 1026 (m), 972 (m), 910 (m), 788 (w), 760 (m), 732 (s), 713 (s); **HRMS** (ESI) m/z $[M+Na]^+$ calcd for $C_{45}H_{49}FO_{13}Na^+$ 839.3049; found 839.3070.

3-*O*-benzoyl-6-*O*-benzyl-4-*O*-fluorenylmethoxycarbonyl-2-deoxy-2-fluoro-α-D-mannopyranoside-(1→6)-1,2,3,4-tetra-*O*-methyl-α-D-glucopyranoside (10)

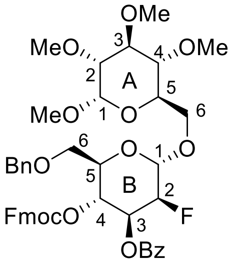

10

Donor **8** (30.9 mg, 43.8 µmol, 1.0 eq.) and acceptor **6**[4] (15.5 mg, 65.8 µmol, 1.5 eq.) were co-evaporated twice with toluene and dried *in vacuo*. Freshly activated 4 Å MS (30 mg) and dry DCM (1 mL) were added, and the mixture was cooled to 0 °C. NIS (12.3 mg, 54.8 µmol, 1.25 eq.) and TfOH (1.93 µL, 21.9 µmol, 0.5 eq.) were successively added at 0 °C under argon, and the dark purple mixture was stirred at 0 °C for 1 h. The reaction was allowed to warm to rt and was quenched by addition of solid $NaHCO_3$ and solid $Na_2S_2O_3$. The colourless suspension was filtered, concentrated *in vacuo* and purified by column chromatography (SiO_2, CyH/EtOAc 4/1 → 3/2) to afford disaccharide **10** (34.0 mg, 41.6 µmol, 95 %) as a white sticky foam.

R$_f$ (CyH/EtOAc 3/2) 0.25; **[α]$_D^{24}$** +45.8 (c = 1.0, CH_2Cl_2); **^{1}H NMR** (500 MHz, $CDCl_3$) δ 8.06 – 8.01 (m, 2H, Bz), 7.71 (ddt, *J* = 7.6, 6.8, 0.9 Hz, 2H, Fmoc), 7.49 (ddt, *J* = 8.7, 7.1, 1.3 Hz, 1H, Bz), 7.42 (tq, *J* = 7.6, 0.9 Hz, 2H, Fmoc), 7.38 – 7.31 (m, 6H, Bz, Fmoc, Bn), 7.31 – 7.27 (m, 2H, Bn), 7.24 – 7.18 (m, 2H, Bn, Fmoc), 7.15 (td, *J* = 7.5, 1.1 Hz, 1H, Fmoc), 5.57 (ddd, J_{FH} = 28.1 HZ and *J* = 10.2, 2.5 Hz, 1H, H-C3B), 5.49 (td, *J* = 10.1, 1.2 Hz, 1H, H-C4B), 5.20 (dd, J_{FH} = 6.9 Hz and *J* = 1.9 Hz, 1H, H-C1B), 5.01 (dt, J_{FH} = 50.0 Hz and *J* = 2.2 Hz, 1H, H-C2B), 4.81 (d, *J* = 3.6 Hz, 1H, H-C1A), 4.64 (d, *J* = 12.1 Hz, 1H, C*H*$_2$Ph), 4.56 (d, *J* = 12.1 Hz, 1H, C*H*$_2$Ph), 4.29 (dd, *J* = 10.4, 7.3 Hz, 1H, C*H*$_2$ Fmoc), 4.22 (dt, *J* = 9.9, 3.9 Hz, 1H, H-C5B), 4.17 (dd, *J* = 10.5, 7.7 Hz, 1H, C*H*$_2$ Fmoc), 4.00 (t, *J* = 7.6 Hz, 1H, C*H* Fmoc), 3.98 (dd, *J* = 11.2, 4.7 Hz, 1H, H-C6A), 3.79 (dd, *J* = 11.2, 1.9 Hz, 1H, H-C6A), 3.74 (dd, *J* = 10.9, 4.7 Hz, 1H, H-C6B), 3.71 (dd, *J* = 11.0, 3.3 Hz, 1H, H-C6B), 3.68 (ddd, *J* = 10.2, 4.7, 1.9 Hz, 1H, H-C5A), 3.65 (s, 3H, OMe), 3.60 (s, 3H, OMe), 3.55 (t, *J* = 9.2 Hz, 1H, H-C3A), 3.54 (s, 3H, OMe), 3.45 (s, 3H, OMe), 3.23 (dd, *J* = 9.6, 3.6 Hz, 1H, H-C2A), 3.15 (dd, *J* = 10.1, 8.8 Hz, 1H, H-C4A) ppm; **^{13}C NMR** (126 MHz, $CDCl_3$) δ 165.7 (Bz), 154.4 (Fmoc), 143.3 (Fmoc), 143.1 (Fmoc), 141.3 (Fmoc), 141.2 (Fmoc), 138.0 (Bn), 133.6 (Bz), 130.1 (Bz), 129.2 (Bz), 128.5 (Bz), 128.4 (Bn), 127.9 (Bn), 127.8 (Fmoc), 127.7 (Bn), 127.23 (Fmoc), 127.21 (Fmoc), 125.2 (Fmoc), 125.1 (Fmoc), 125.05 (Fmoc), 120.06 (Fmoc), 97.6 (d, *J* = 29.0 Hz, C1B), 97.5 (C1A), 87.1 (d, *J* = 179.5 Hz, C2B), 83.7 (C3A), 81.9 (C2A), 79.6 (C4A), 73.7 (CH_2Ph), 71.1 (d, *J* = 16.8 Hz, C3B), 70.8 (C4B), 70.3

(CH_2 Fmoc), 69.9 (C5A), 69.8 (C5B), 68.9 (C6B), 66.7 (C6A), 61,0 (OMe), 60.7 (OMe), 59.2 (OMe), 55.4 (OMe), 46.6 (CH Fmoc) ppm; **^{19}F NMR** (470 MHz, $CDCl_3$) δ -204.77 (ddd, J_{FH} = 50.0, 28.3, 7.0 Hz) ppm; **IR** (neat) $\tilde{\nu}_{max}$ / cm^{-1} 2927 (w), 2247 (w), 1755 (s), 1726 (s), 1602 (w), 1451 (m), 1385 (w), 1316 (w), 1271 (s), 1248 (s), 1195 (m), 1139 (s), 1098 (s), 1069 (s), 1047 (s), 1027 (s), 994 (s), 910 (m), 855 (w), 816 (w), 783 (w), 760 (m), 736 (s), 713 (s), 662 (w); **HRMS** (ESI) m/z $[M+Na]^+$ calcd for $C_{45}H_{49}FO_{13}Na^+$ 839.3049; found 839.3050.

4-*O*-benzyl-3-*O*-fluorenylmethoxycarbonyl-2-deoxy-2-fluoro-6-*O*-levulinoyl-α-D-mannopyranoside-(1→6)-1,2,3,4-tetra-*O*-methyl-α-D-glucopyranoside (11)

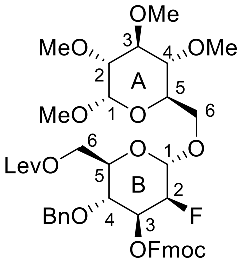

11

Donor **9** (40.3 mg, 57.7 µmol, 1.0 eq.) and **6**[4] (20.4 mg, 86.5 µmol, 1.5 eq.) were co-evaporated twice with toluene and dried *in vacuo*. Freshly activated 4 Å MS (40 mg) and dry DCM (1 mL) were added, and the mixture was cooled to 0 °C. NIS (16.2 mg, 72.1 µmol, 1.25 eq.) and TfOH (2.55 µL, 28.9 µmol, 0.5 eq.) were successively added at 0 °C under argon, and the dark purple mixture was stirred at 0 °C for 1 h. The reaction was allowed to warm to rt and was quenched by addition of solid $NaHCO_3$ and solid $Na_2S_2O_3$. The colourless suspension was filtered, concentrated *in vacuo* and purified by column chromatography (SiO_2, CyH/EtOAc 3/1 → 2/1) to afford disaccharide **11** (39.7 mg, 49.0 µmol, 85 %) as a colourless semi-solid along with a second fraction containing the lactol hydrolysis product (α and β anomers).

R_f (CyH/EtOAc 1/1) 0.22; **$[\alpha]_D^{22}$** +72.5 (c = 1.0, $CHCl_3$); **^{1}H NMR** (599 MHz, $CDCl_3$) δ 7.76 (dd, *J* = 7.6, 3.5 Hz, 2H, Fmoc), 7.62 (d, *J* = 7.8 Hz, 1H, Fmoc), 7.60 (d, *J* = 7.5 Hz, 1H, Fmoc), 7.42 – 7.37 (m, 2H, Fmoc), 7.32 – 7.24 (m, 7H, Fmoc, Bn), 5.11 (ddt, J_{FH} = 28.9 Hz and *J* = 5.9, 2.6 Hz, 1H, H-C3B), 5.09 (dd, J_{FH} = 6.9 Hz and *J* = 2.1 Hz, 1H, H-C1B), 4.89 (dt, J_{FH} = 50.3 Hz and *J* = 2.3 Hz, 1H,H-C2B), 4.78 (d, *J* = 11.1 Hz, 1H, C*H*$_2$Ph), 4.76 (d, *J* = 3.6 Hz, 1H, H-C1A), 4.61 (d, *J* = 11.1 Hz, 1H, C*H*$_2$Ph), 4.45 (dd, *J* = 10.5, 7.4 Hz, 1H, C*H*$_2$ Fmoc), 4.40 (dd, *J* = 10.4, 7.5 Hz, 1H, C*H*$_2$ Fmoc), 4.38 (d, *J* = 12.0 Hz, 1H, H-C6B), 4.34 (dd, *J* = 11.9, 2.9 Hz, 1H, H-C6B), 4.26 (t, *J* = 7.4 Hz, 1H, C*H* Fmoc), 4.00 – 3.94 (m, 2H, H-C4B, H-C5B), 3.87 (dd, *J* = 11.2, 4.9 Hz, 1H, H-C6A), 3.71 (dd, *J* = 11.3, 1.9 Hz, 1H, H-C6A), 3.63 – 3.60 (m, 1H, H-C5A), 3.61 (s, 3H, OMe), 3.53 (s, 3H, OMe), 3.50 (s, 3H, OMe), 3.49 (t, *J* = 9.1 Hz, 1H, H-C3A), 3.38 (s, 3H, OMe), 3.17 (dd, *J* = 9.6, 3.6 Hz, 1H, H-C2A), 3.06 (dd, *J* = 10.1, 8.8 Hz, 1H, H-C4A), 2.83 – 2.68 (m, 2H, Lev), 2.66 – 2.57 (m, 2H, Lev), 2.20 (s, 3H, Lev) ppm; **^{13}C NMR** (151 MHz, $CDCl_3$) δ 206.5 (C=O, Lev), 172.6 (C=O, Lev), 154.4 (C=O, Fmoc), 143.4 (Fmoc), 143.3 (Fmoc), 141.5 (Fmoc), 141.4 (Fmoc), 137.6 (Bn), 128.6 (Bn), 128.2 (Bn), 128.1 (Bn, Fmoc), 127.3 (Fmoc), 125.3 (Fmoc), 125.2 (Fmoc), 120.2 (Fmoc), 97.5 (d, *J* = 28.8 Hz, C1B), 97.4 (C1A), 87.1 (d, *J* = 177.7 Hz; C2B), 83.7 (C3A), 81.9 (C2A), 79.6 (C4A), 77.0 (d, *J* = 16.3 Hz, C3B), 75.3 (CH_2Ph), 72.5 (C4B), 70.4 (CH_2 Fmoc), 69.9 (C5B), 69.8 (C5A), 66.6 (C6A), 62.9 (C6B), 61.0 (OMe), 60.7 (OMe), 59.1 (OMe), 55.3 (OMe), 46.8 (CH Fmoc), 38.0 (Lev), 30.0 (Lev), 28.0 (Lev) ppm; **^{19}F NMR** (564 MHz, $CDCl_3$) δ -204.74 (ddd, J_{FH} = 50.3, 28.9, 7.0 Hz) ppm; **IR** (neat) $\tilde{\nu}_{max}$ / cm^{-1} 2933 (w), 1747 (m), 1718 (s), 1452 (w), 1358 (w), 1260 (s), 1157 (m), 1098 (s), 975 (m), 813 (w), 786 (w), 742 (m), 700 (w); **HRMS** (ESI) m/z $[M+Na]^+$ calcd for $C_{43}H_{51}FO_{14}Na^+$ 833.3155; found 833.3154.

Fmoc deprotection in solution:
Isopropyl 3-*O*-benzoyl-4-*O*-benzyl-2-deoxy-2-fluoro-α-D-mannopyranoside (S12)

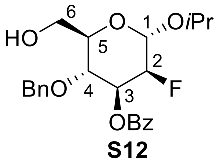

S12

2 (129.7 mg, 0.2018 mmol, 1.0 eq.) was glycosylated to 2-propanol (328 µL, 4.29 mmol, 21 eq.) according to the general procedure for glycosylations with methanol. As the reaction did not go to completion as judged by crude NMR analysis, the crude mixture was re-submitted to the glycosylation conditions until full conversion to the α-glycosylation product was observed. The crude glycosylation product was dissolved in DMF (2 mL) and piperidine (50 µL, 0.506 mmol, 2.5 eq.) was added. The mixture was stirred at rt for 30 min, diluted with DCM (40 mL), washed with 1 m HCl and brine, dried over $MgSO_4$ and concentrated *in vacuo*. The crude product was purified by column chromatography (SiO_2, CyH/EtOAc 9/1 → 7/1) to afford the desired alcohol **S12** (82.4 mg, 0.197 mmol, 98 %) as a colourless syrup.

R_f (CyH/EtOAc 4/1) 0.13; **$[\alpha]_D^{22}$** +9.2 (c = 1.0, $CHCl_3$); **^{1}H NMR** (500 MHz, $CDCl_3$) δ 8.11 – 8.06 (m, 2H, Bz), 7.62 – 7.56 (m, 1H, Bz), 7.50 – 7.44 (m, 2H, Bz), 7.25 – 7.16 (m, 5H, Bn), 5.58 (ddd, J_{FH} = 29.5 Hz and *J* = 9.9, 2.6 Hz, 1H, H-C3), 5.09 (dd, J_{FH} = 7.4 Hz and *J* = 2.0 Hz, 1H, H-C1), 4.82 (dt, J_{FH} = 50.6 Hz and *J* = 2.3 Hz, 1H, H-C2), 4.73 (d, *J* = 10.9 Hz, 1H, C*H*$_2$Ph), 4.66 (d, *J* = 10.9 Hz, 1H, C*H*$_2$Ph), 4.16 (t, *J* = 9.8 Hz, 1H, H-C4), 3.96 (sept, *J* = 6.2 Hz, 1H, O*i*Pr), 3.91 – 3.86 (m, 2H, H-C5, H-C6), 3.84 (dd, *J* = 12.0, 3.9 Hz, 1H, H-C6), 1.23 (d, *J* = 6.2 Hz, 3H, O*i*Pr), 1.18 (d, *J* = 6.1 Hz, 3H, O*i*Pr) ppm; **^{13}C NMR** (126 MHz, $CDCl_3$) δ 165.8 (C=O, Bz), 137.7 (Bn), 133.5 (Bz), 130.0 (Bz), 129.8 (Bz), 128.7 (Bz), 128.6 (Bn), 128.2 (Bn), 128.1 (Bn), 95.6 (d, *J* = 28.8 Hz, C1), 88.4 (d, *J* = 177.4 Hz, C2), 75.4 (CH_2Ph), 73.4 (d, *J* = 16.7 Hz. C3), 73.0 (C4), 72.0 (C5), 70.4 (O*i*Pr), 61.9 (C6), 23.3 (O*i*Pr), 21.4 (O*i*Pr) ppm; **^{19}F NMR** (470 MHz, $CDCl_3$) δ -203.52 (ddd, J_{FH} = 50.6, 29.5, 7.4 Hz) ppm; **IR** (neat) $\tilde{\nu}_{max}$ / cm^{-1} 3418 (w), 2973 (w), 2924 (w), 1722 (s), 1602 (w), 1586 (w), 1496 (w), 1452 (m), 1382 (w), 1315 (w), 1267 (s), 1178

(w), 1115 (s), 1067 (s), 1027 (s), 975 (m), 925 (m), 846 (m), 798 (m), 749 (m), 712 (s), 699 (s); **HRMS** (ESI) m/z $[M+Na]^+$ calcd for $C_{23}H_{27}FO_6Na^+$ 441.1684; found 441.1704.

3-*O*-benzoyl-4-*O*-benzyl-6-*O*-fluorenylmethoxycarbonyl-2-deoxy-2-fluoro-α-D-mannopyranoside-(1→6)- 3-*O*-benzoyl-4-*O*-benzyl-2-deoxy-2-fluoro-1-*O*-isopropyl-α-D-mannopyranoside (S13)

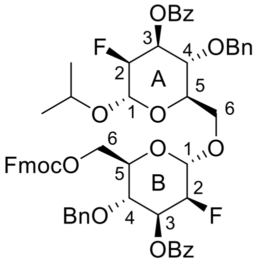

S13

Donor **2** (162.4 mg, 0.252 mmol, 6.0 eq.) and acceptor **S12** (17.6 mg, 42.1 µmol, 1.0 eq.) were co-evaporated twice with toluene and dried *in vacuo*. Freshly activated 4 Å MS (150 mg) and dry DCM (3 mL) were added, and the mixture was cooled to 0 °C. NIS (85.2 mg, 0.379 mmol, 9.0 eq.) and TfOH (22.2 µL, 0.252 mmol, 6.0 eq.) were successively added at 0 °C under argon, and the dark purple mixture was stirred at 0 °C for 2 h. The reaction was quenched by addition of solid $NaHCO_3$ and solid $Na_2S_2O_3$ and was allowed to warm to rt. The colourless suspension was filtered, concentrated *in vacuo* and purified by column chromatography (SiO_2, CyH/EtOAc 9/1) to afford disaccharide **S13** (23.7 mg, 23.7 µmol, 56 %) as a white semi solid. In addition to the desired disaccharide and hydrolysed donor, the 1,1-homocoupling product (23.6 mg, 20.0 µmol, 16 % relative to donor, mixture of α,α- and α,β-anomers) of donor **2** was isolated.

$\mathbf{R_f}$ (CyH/EtOAc 4/1) 0.43; $\mathbf{[\alpha]_D^{22}}$ +15.8 (c = 1.0, $CHCl_3$); ^{1}**H NMR** (599 MHz, $CDCl_3$) δ 8.15 – 8.11 (m, 4H, Bz), 7.78 (dd, *J* = 7.6, 1.6 Hz, 2H, Fmoc), 7.67 (dq, *J* = 7.5, 0.9 Hz, 1H, Fmoc), 7.65 (dq, *J* = 7.5, 0.9 Hz, 1H, Fmoc), 7.63 – 7.58 (m, 2H, Bz), 7.51 – 7.45 (m, 4H, Bz), 7.43 – 7.40 (m, 2H, Fmoc), 7.33 (td, *J* = 7.5, 1.2 Hz, 1H, Fmoc), 7.33 (td, *J* = 7.5, 1.2 Hz, 1H, Fmoc), 7.24 – 7.15 (m, 10H, Bn), 5.63 (ddd, J_{FH} = 29.3 Hz and *J* = 9.7, 2.6 Hz, 1H, H-C3B), 5.57 (ddd, J_{FH} = 29.4 Hz and *J* = 9.7, 2.6 Hz, 1H, H-C3A), 5.24 (dd, *J* = 7.0, 1.9 Hz, 1H, H-C1B), 5.05 (dd, *J* = 7.5, 1.9 Hz, 1H, H-C1A), 4.99 (dt, J_{FH} = 50.0 Hz and *J* = 2.3 Hz, 1H, H-C2B), 4.80 (dt, J_{FH} = 50.5 Hz and *J* = 2.1 Hz, 1H, H-C2A), 4.77 (d, *J* = 11.0 Hz, 1H, C*H*$_2$Ph), 4.74 (d, *J* = 10.9 Hz, 1H, C*H*$_2$Ph), 4.63 (d, *J* = 11.0 Hz, 2H, C*H*$_2$Ph), 4.53 (dd, *J* = 11.7, 2.2 Hz, 1H, H-C6B), 4.46 (dd, *J* = 10.4, 7.4 Hz, 1H, C*H*$_2$ Fmoc), 4.39 (dd, *J* = 11.7, 4.3 Hz, 1H, H-C6B), 4.38 (dd, *J* = 10.4, 7.7 Hz, 1H, C*H*$_2$ Fmoc), 4.30 (t, *J* = 7.5 Hz, 1H, C*H* Fmoc), 4.17 (t, *J* = 9.8 Hz, 1H, H-C4B), 4.11 (t, *J* = 9.9 Hz, 1H, H-C4A), 4.06 (ddd, *J* = 10.1, 4.4, 2.1 Hz, 1H, H-C5B), 4.02 – 3.93 (m, 3H, H-C5A, H-C6A, O*i*Pr), 3.87 – 3.83 (m, 1H, H-C6A), 1.25 (d, *J* = 6.2 Hz, 3H, O*i*Pr), 1.16 (d, *J* = 6.1 Hz, 3H, O*i*Pr) ppm; ^{13}**C NMR** (151 MHz, $CDCl_3$) δ 165.8 (C=O, Bz), 165.7 (C=O, Bz), 155.2 (C=O, Fmoc), 143.6 (Fmoc), 143.5 (Fmoc), 141.5 (Fmoc), 141.4 (Fmoc), 137.6 (Bn), 137.5 (Bn), 133.6 (Bz), 133.5 (Bz), 130.1 (Bz), 130.0 (Bz), 129.79 (Bz), 129.78 (Bz), 128.72 (Bz), 128.66 (Bz), 128.58 (Bn), 128.57 (Bn), 128.2 (Bn), 128.12 (Bn), 128.10 (Bn), 128.05 (Bn), 128.04 (Fmoc), 127.4 (Fmoc), 127.3 (Fmoc), 125.5 (Fmoc), 125.4 (Fmoc), 120.2 (Fmoc), 97.8 (d, *J* = 28.9 Hz, C1B), 95.5 (d, *J* = 29.0 Hz, C1A), 88.3 (d, *J* = 177.7 Hz, C2A), 87.6 (d, *J* = 177.1 Hz, C2B), 75.43 (CH_2Ph), 75.38 (CH_2Ph), 73.5 (d, *J* = 16.8 Hz, C3A), 73.21 (d, *J* = 16.5 Hz, C3B), 73.20 (C4A), 73.0 (C4B), 71.5 (C5A), 70.7 (O*i*Pr), 70.3 (CH_2 Fmoc), 70.1 (C5B), 66.4 (C6B), 66.2 (C6A), 46.9 (CH Fmoc), 23.3 (O*i*Pr), 21.5 (O*i*Pr) ppm; ^{19}**F NMR** (564 MHz, $CDCl_3$) δ -203.16 (ddd, J_{FH} = 50.6, 29.4, 7.5 Hz, F-C2A), -204.30 (ddd, J_{FH} = 50.1, 29.3, 7.0 Hz, F-C2B) ppm; **IR** (neat) $\tilde{\nu}_{max}$ / cm^{-1} 2927 (w), 1736 (m), 1725 (s), 1452 (m), 1264 (s), 1094 (s), 1069 (s), 1026 (m), 974 (m), 742 (m), 713 (s); **HRMS** (ESI) m/z $[M+Na]^+$ calcd for $C_{58}H_{56}F_2O_{13}Na^+$ 1021.3581; found 1021.3576.

Fluorine-Directed Automated Glycan Assembly (FDAGA)

AGA was performed on a Glyconeer 2.1™ using the GlycoSoft Software package. Default conditions for BB **12**[6] and pre-configured reaction cycles (see below) were used as a starting point to optimise conditions for the AGA of known BB **12** in our lab. In brief, glycosylation time, equivalents of acid and equivalents of BB were varied to achieve quantitative yield of a 1,6-linked dimannoside (judged by ^{1}H NMR). As opposed to previous reports from the Seeberger group[6], a higher amount of TfOH (see below, Activator solution C) and 8 eq. of the BB were found to be necessary to achieve reproducible results with our platform. Under these conditions, the fluorinated BBs performed similar to the non-fluorinated parent BBs. The detailed conditions for FDAGA are given in the following.

Stock Solutions for AGA

A. Building block solutions: The required amount of building block (0.10 mmol or 0.0625 mmol) was dissolved in 1 mL dry DCM.
B. Acid wash solution: TMSOTf (450 µL) was dissolved in dry DCM (40 mL) under argon and kept at 2 °C during the automated synthesis.
C. Activator solution: Recrystallised NIS (1.35 g) was dissolved in dry DCM/1,4-dioxane (2/1, v/v, 40 mL) under argon. TfOH (150 µL) was added and the solution was kept under argon at 2 °C during the automated synthesis.

D. Fmoc deprotection solution: Piperidine (20 mL) was dissolved in DMF (80 mL).
E. Pre-capping solution: Pyridine (4 mL) was dissolved in DMF (36 mL).
F. Capping solution: Acetic anhydride (4 mL) and methanesulfonic acid (800 µL) were dissolved in dry DCM (36 mL).

Modules for AGA

Module A: Resin swelling
Resin (12.5 µmol) was placed in the reaction vessel and protected from light throughout the automation run. Prior to the synthesis, the resin was swollen in DCM (2 mL) for 30 min at rt and washed with DMF, THF and DCM (3 x 2 mL for 25 s each). During the resin swelling, all reagent lines were washed and primed.
Module B: Acid wash
The resin was swollen in DCM (2 mL) and cooled to -20 °C. Solution B (1 mL) was added and incubated at -20 °C (2 x 90 s). The solution was drained and the resin was washed with DCM (2 mL for 25 s).
Module C: Thioglycoside Glycosylation
The resin was kept in DCM (2 mL) and the temperature was set to T_1. After the T_1 was reached, the DCM was drained and building block solution A (0.10 mmol, 8.0 eq. in 1 mL dry DCM) was added. Activator solution C (1 mL) was added dropwise and the glycosylation was kept at T_1 for 5 min. Thereon, the mixture was heated to T_2 and glycosylated at this temperature for the specified incubation time. The solution was drained and the resin was washed with DCM/1,4-dioxane (1/1, v/v) and DCM (2 x 2 mL for 25 s each).

Command	Cycles	Solution	Amount	T / °C	Incubation time
**Cool**	--	--	--	T_1	--
**Deliver**	1	BB solution A	1 mL	T_1	--
**Deliver**	1	activator solution C	1 mL	T_1	--
**Incubation**	1	--	--	T_1	5 min
**Incubation**	1	--	--	T_2	20-40 min
**Discharge**	1	--	--	T_2	--
**Heat**	--	--	--	25	--
**Wash**	2	DCM/dioxane (1/1, v/v)	2 mL	T_2 - 25	25 s
**Wash**	2	DCM	2 mL	T_2 - 25	25 s

Module D: Acidic capping
The temperature was set to 25 °C and the resin was washed with DMF (3 x 2 mL for 25 s). Pre-capping solution E (2 mL) was added to the reaction vessel. After 1 min, the solution was drained and the resin was washed with DCM (3 x 2 mL for 25 s). Capping solution F (2 mL) was added to the reaction vessel, incubated to 20 min and drained. After the capping the resin was washed with DCM (3 x 2 ml for 25 s).
Module E: Fmoc deprotection
The temperature was set to 25 °C and the resin was washed with DMF (3 x 2 mL for 60 s). Fmoc deprotection solution D (2 mL) was delivered to the reaction vessel and incubated for 5 min. The deprotection solution was drained to the UV detector and the resin was washed with DMF (3 x 2 mL for 60 s, then 1 x 15 mL) and DCM (5 x 2 mL for 60 s).

Post-Automation Manipulations

Photocleavage: The oligosaccharides were cleaved from the solid support using a custom-build LED-flow-reactor as described previously.[1]
Purification: The crude oligosaccharides were purified by flash chromatography or analytical HPLC (*Büchi Pure C-850 FlashPrep*).
Method A: The crude oligosaccharides were purified by flash chromatography [FlashPure ID Silica 4g; CyH (1 min), linear gradient to 6 % *i*PrOH (6 min), 10 % *i*PrOH (3 min)] to remove grease and excess linker.
Method B: The crude oligosaccharides were purified by flash chromatography [FlashPure ID Silica 4g; CyH / 2 % *i*PrOH (1 min), linear gradient to 10 % *i*PrOH (7 min), 15 % *i*PrOH (8.5 min)].
Method C: The crude oligosaccharides were purified by RP-HPLC [Zorbax Eclipse Plus C18 (4.6 × 100 mm, 3.5 µm), 0.1 % formic acid in H_2O (5 min), linear gradient to 30 % MeCN (30 min), linear gradient to 90 % MeCN (5 min), 90 % MeCN (20 min)].

Global deprotection

Module G: Methanolysis
The fully protected oligosaccharide was dissolved in DCM/MeOH (1/1, v/v, 1 mL) and NaOMe (4 mg) was added. The mixture was stirred at rt for 14 h, neutralised with amberlyst [H^+], filtered and concentrated *in vacuo*.
Module H: Hydrogenolysis
The partially protected oligosaccharide was dissolved in EtOAc/*t*BuOH/water (2/1/1, v/v/v, 2 mL) and Pd/C (10 wt-%, same amount as crude saccharide, pre-washed with HCl[5]) was added. The atmosphere was replaced by hydrogen and the mixture was stirred at rt under an atmosphere of hydrogen for 14 h. The reaction mixture was filtered over celite®, washed with EtOAc, *t*BuOH and water, and concentrated *in vacuo*.

Automated Syntheses

Tetramannoside 13

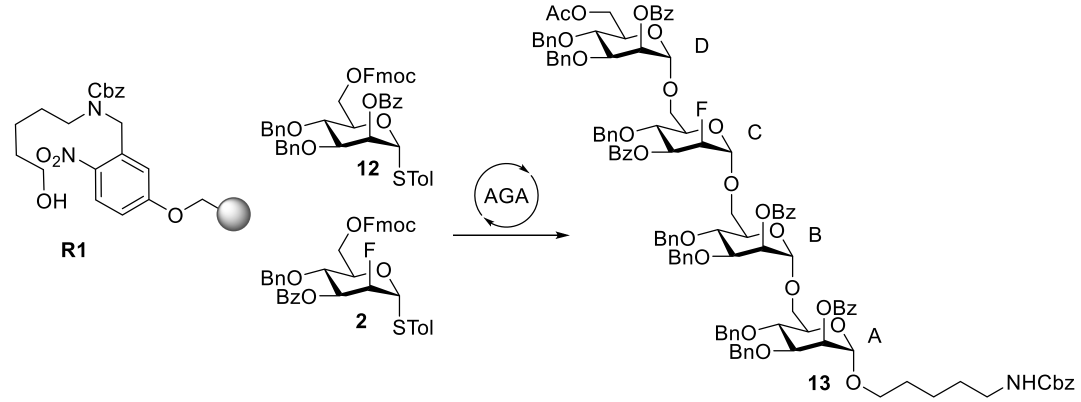

	Module	Conditions
	A: Resin Swelling	
2	B: Acid Wash<br>C: Thioglycoside Glycosylation<br>D: Acidic capping<br>E: Fmoc deprotection	BB **12** (8.0 eq.), 0 °C for 5 min, 20 °C for 20 min
	B: Acid Wash<br>C:Thioglycoside Glycosylation<br>D: Acidic capping<br>E: Fmoc deprotection	BB **2** (8.0 eq.), 0 °C for 5 min, 20 °C for 20 min
	B: Acid Wash<br>C: Thioglycoside Glycosylation<br>D: Acidic capping<br>E: Fmoc deprotection	BB **12** (8.0 eq.), 0 °C for 5 min, 20 °C for 20 min
	D: Acidic capping	

Tetrasaccharide **13** was cleaved from solid support as described in the post-automation manipulations. Removal of excess linker and grease by flash chromatography (Method A) afforded tetrasaccharide **13** as a white semi-solid (15.2 mg, 7.7 µmol, 62 % based on resin).

^{1}H NMR (599 MHz, $CDCl_3$) δ 8.14 – 8.04 (m, 8H, Bz), 7.64 – 7.56 (m, 2H, Bz), 7.50 – 7.39 (m, 10H, Bz), 7.38 – 7.19 (m, 32H, Bn, Cbz), 7.17 – 7.06 (m, 8H, Bn, Cbz), 5.74 (q, J = 3.2 Hz, 2H, H-C2B, H-C2D), 5.63 (dd, J = 3.3, 1.8 Hz, 1H, H-C2A), 5.58 (ddd, J_{FH} = 29.2 Hz and J = 9.9, 2.3 Hz, 1H, H-C3C), 5.17 (dd, J = 7.0, 1.7 Hz, 1H, H-C1C), 5.11 – 5.07 (m, 2H, CH_2 Cbz), 5.06 (d, J = 1.8 Hz, 1H, H-C1B), 5.05 (d, J = 2.0 Hz, 1H, H-C1D), 4.99 (d, J = 11.3 Hz, 1H, CH_2Ph), 4.95 (t, J_{FH} = 50.2 Hz and J = 2.3 Hz, 1H, H-C2C), 4.92 – 4.87 (m, 3H, H-C1A, CH_2Ph), 4.83 (d, J = 11.0 Hz, 1H, CH_2Ph), 4.82 (d, J = 11.6 Hz, 1H, CH_2Ph), 4.74 (d, J = 11.4 Hz, 1H, CH_2Ph), 4.68 (d, J = 11.2 Hz, 1H, CH_2Ph), 4.60 (d, J = 11.4 Hz, 1H, CH_2Ph), 4.59 (d, J = 11.4 Hz, 1H, CH_2Ph), 4.58 (d, J = 11.0 Hz, 1H,

CH_2Ph), 4.56 (d, *J* = 11.0 Hz, 1H, CH_2Ph), 4.48 (d, *J* = 12.0 Hz, 1H, CH_2Ph), 4.46 (d, *J* = 11.5 Hz, 1H, CH_2Ph), 4.45 (d, *J* = 11.2 Hz, 1H, CH_2Ph), 4.22 (d, *J* = 3.1 Hz, 2H, H-C6D), 4.12 (t, *J* = 9.9 Hz, 1H, H-C4C), 4.11 – 4.08 (m, 2H, H-C3A, H-C3D), 4.06 (dd, *J* = 9.3, 3.2 Hz, 1H, H-C3B), 4.00 (t, *J* = 9.6 Hz, 1H, H-C4B), 3.96 (dd, *J* = 11.4, 4.8 Hz, 1H, H-C6A), 3.93 (t, *J* = 9.5 Hz, 1H, H-C4A), 3.89 (t, *J* = 9.6 Hz, 1H, H-C4D), 3.87 – 3.74 (m, 7H, H-C5A, H-C6A, H-C5B, H-C6B, H-C5C, H-C6C, H-C5D), 3.70 – 3.65 (m, 1H, OCH_2 linker), 3.64 (d, *J* = 11.4 Hz, 1H, H-C6B), 3.57 (dd, *J* = 11.8, 1.6 Hz, 1H, H-C6C), 3.41 (dd, *J* = 15.8, 6.6 Hz, 1H, OCH_2 linker), 3.17 (dd, *J* = 13.4, 6.5 Hz, 2H, NCH_2 linker), 2.02 (s, 3H, Ac), 1.62 – 1.46 (m, 4H, linker), 1.40 – 1.31 (m, 2H, linker) ppm; **^{13}C NMR** (151 MHz, $CDCl_3$) δ 170.8 (C=O, Ac), 166.0, 165.8, 165.6, 165.4 (C=O, Bz), 156.5 (C=O, Cbz), 138.6, 138.5, 138.1, 137.82, 137.75, 137.7, 133.5, 133.38, 133.36, 133.3, 130.13, 130.10, 130.05, 129.98, 129.96, 129.9, 129.8, 128.70, 128.67, 128.65, 128.63, 128.58, 128.55, 128.53, 128.50, 128.48, 128.46, 128.43, 128.40, 128.3, 128.24, 128.18, 128.17, 128.0, 127.90, 127.87, 127.78, 127.76, 127.72, 127.71, 127.6 (Carom), 98.7 (C1D), 98.05 (C1B), 97.96 (C1A), 97.7 (d, *J* = 29.5 Hz, C1C), 87.6 (d, *J* = 175.5 Hz, C2C), 78.8 (C3A), 78.2 (C3B), 77.5 (C3D), 75.29, 75.26, 75.2 (CH_2Ph), 74.4 (C4A), 74.0 (C4B), 73.6 (d, *J* = 18.9 Hz, C3C), 73.5 (C4D), 72.7 (C4C), 71.8 (CH_2Ph), 71.5 (C5B), 71.4 (CH_2Ph), 71.3 (C5C), 71.2 (CH_2Ph), 70.9 (C5A), 70.2 (C5D), 69.2 (C2A), 68.8 (C2B), 68.4 (C2D), 67.8 (OCH_2 linker), 66.7 (CH_2 Cbz), 66.4 (C6A), 66.0 (C6C), 65.9 (C6B), 63.2 (C6D), 41.1 (NCH_2 linker), 29.8 (linker), 29.2 (linker), 23.6 (linker), 20.9 (Ac) ppm; **^{19}F NMR** (564 MHz, $CDCl_3$) δ -204.49 (ddd, J_{FH} = 50.4, 29.2, 7.0 Hz) ppm; **HRMS** (ESI) m/z $[M+Na]^+$ calcd for $C_{116}H_{118}FNO_{27}Na^+$ 1999.7801; found 1999.7797.

Crude ^{19}F{^{1}H} NMR of the automated synthesis of **13:**

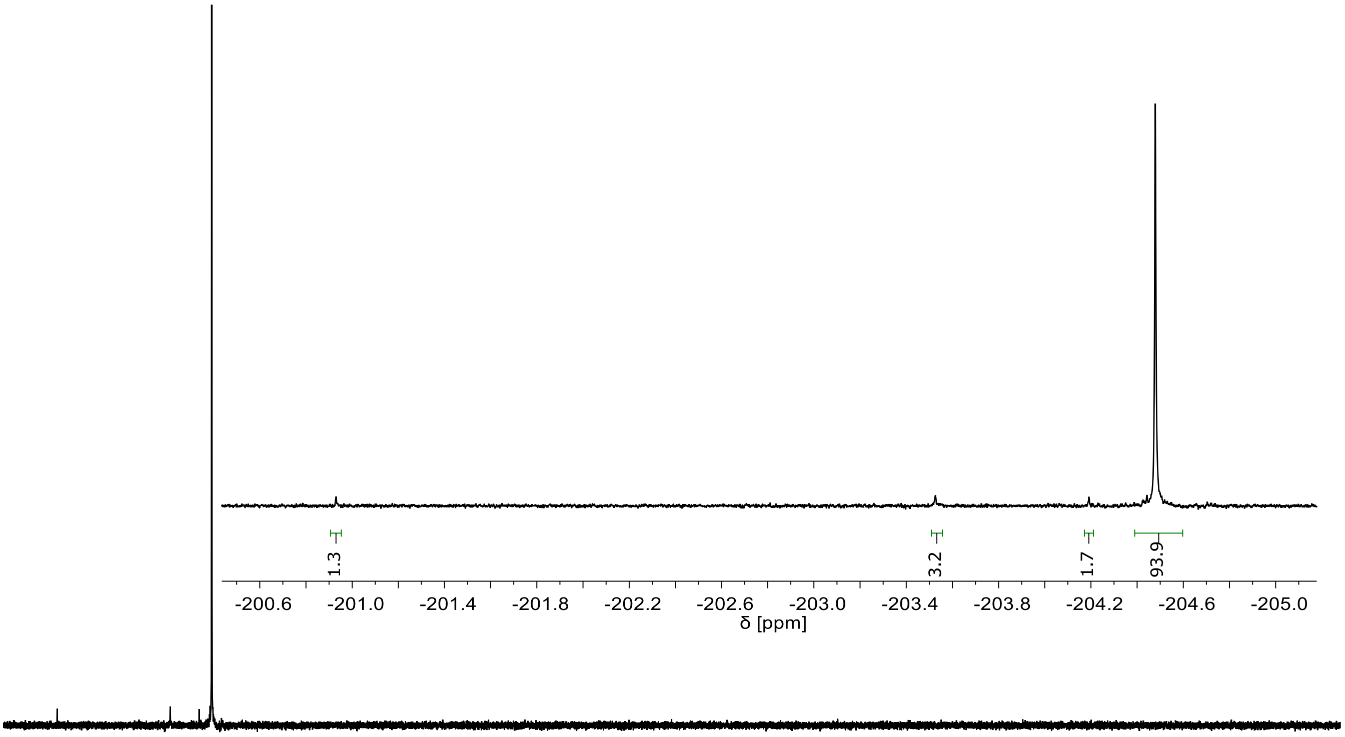

Tetramannoside 17

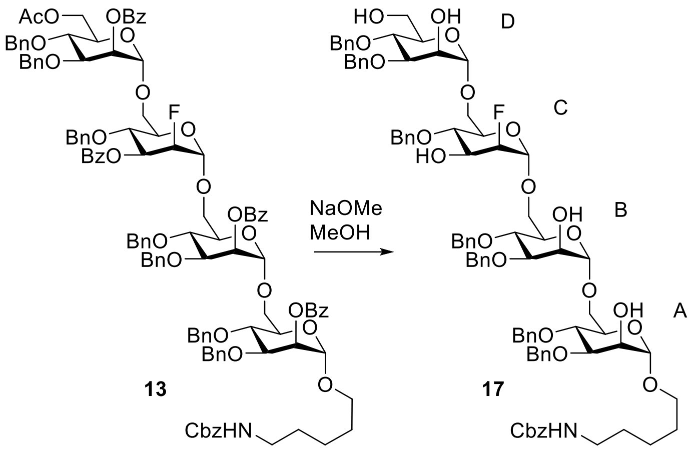

The ester PGs of tetramannoside **13** (15.2 mg, 7.69 µmol, 1.0 eq.) were cleaved using the conditions of module G to give semi-protected tetramannoside **17** (7.8 mg, 5.1 µmol, 67 %) after purification using method B.

^{1}H NMR (599 MHz, CDCl$_3$) δ 7.38 – 7.17 (m, 40H, Bn, Cbz), 5.07 (s, 2H, CH_2 Cbz), 5.03 (d, J = 1.7 Hz, 1H, H-C1D), 4.94 (s, 1H, H-C1B), 4.91 (dd, J_{FH} = 6.7 Hz and J = 1.7 Hz, 1H, H-C1C), 4.89 (d, J = 11.0 Hz, 1H, CH_2Ph), 4.88 (d, J = 11.3 Hz, 1H, CH_2Ph), 4.82 (d, J = 11.4 Hz, 1H, CH_2Ph), 4.79 (d, J = 1.6 Hz, 1H, H-C1A), 4.75 (d, J = 11.1 Hz, 1H, CH_2Ph), 4.72 – 4.61 (m, 7H, CH_2Ph), 4.59 (dt, J_{FH} = 50.2 Hz and J = 2.3 Hz, 1H, H-C2C), 4.57 (d, J = 11.4 Hz, 1H, CH_2Ph), 4.51 (d, J = 11.3 Hz, 1H, CH_2Ph), 4.34 (d, J = 11.0 Hz, 1H, CH_2Ph), 4.11 (t, J = 2.3 Hz, 1H, H-C2D), 4.11 (dd, J_{FH} = 31.3 Hz and J = 10.4 Hz, 1H, H-C3C), 4.07 (dd, J = 3.3, 1.7 Hz, 1H, H-C2B), 4.00 (dd, J = 3.4, 1.6 Hz, 1H, H-C2A), 3.94 (dd, J = 10.1, 5.9 Hz, 1H, H-C5C), 3.89 (dd, J = 9.2, 3.0 Hz, 1H, H-C3D), 3.89 – 3.82 (m, 4H, H-C3A, H-C6A, H-C5B, H-C4D), 3.82 – 3.77 (m, 2H, H-C3B, H-C6C), 3.77 – 3.73 (m, 2H, H-C6B, H-C6D), 3.72 – 3.66 (m, 4H, H-C5A, H-C6A, H-C6C, H-C6D), 3.66 – 3.60 (m, 2H, H-C6B, H-C5D), 3.60 – 3.51 (m, 4H, H-C4A, H-C4B, H-C4C, OCH_2 linker), 3.35 (q, J = 6.9 Hz, 1H, OCH_2 linker), 3.13 (s, 2H, NCH_2 linker), 2.58 (br s, 5H, OH), 1.54 – 1.41 (m, 4H, linker), 1.34 – 1.28 (m, 2H, linker) ppm; **^{13}C NMR** (151 MHz, CDCl$_3$) δ 156.5 (C=O, Cbz), 138.5, 138.4, 138.3, 138.2, 137.9, 137.7, 136.7, 128.73, 128.71, 128.66, 128.62, 128.60, 128.54, 128.52, 128.3, 128.2, 128.14, 128.10, 128.05, 127.98, 127.95, 127.91, 127.86, 127.80, 127.78, 127.75 (Carom), 98.9 (C1B), 98.82 (C1D), 98.78 (C1A), 96.6 (d, J = 29.5 Hz, C1C), 90.2 (d, J = 174.0 Hz, C2C), 80.4 (C3A), 80.1 (C3B), 79.9 (C3D), 76.0 (C4C), 75.3, 75.2, 75.15, 75.07 (CH$_2$Ph), 74.5 (C4B), 74.4 (C4A), 74.3 (C4D), 72.1, 72.0, 71.9 (CH$_2$Ph), 71.9 (C5D), 71.1 C5B), 71.0 (d, J = 17.6 Hz, C3C), 70.8 (C5A), 69.9 (C5C), 68.6 (C2A), 68.23 (C2D), 68.20 (C2B), 67.5 (OCH$_2$ linker), 67.0 (C6A), 66.8 (CH$_2$ Cbz), 66.5 (C6B), 65.7 (C6C), 62.3 (C6D), 41.1 (NCH$_2$ linker), 29.8, 29.0, 23.5 (linker) ppm; **^{19}F NMR** (564 MHz, CDCl$_3$) δ -206.66 (ddd, J_{FH} = 50.1, 31.2, 6.8 Hz) ppm; **HRMS** (ESI) m/z [M+Na]$^+$ calcd for $C_{86}H_{100}FNO_{22}Na^+$ 1540.6613; found 1540.6600.

Tetramannoside 18

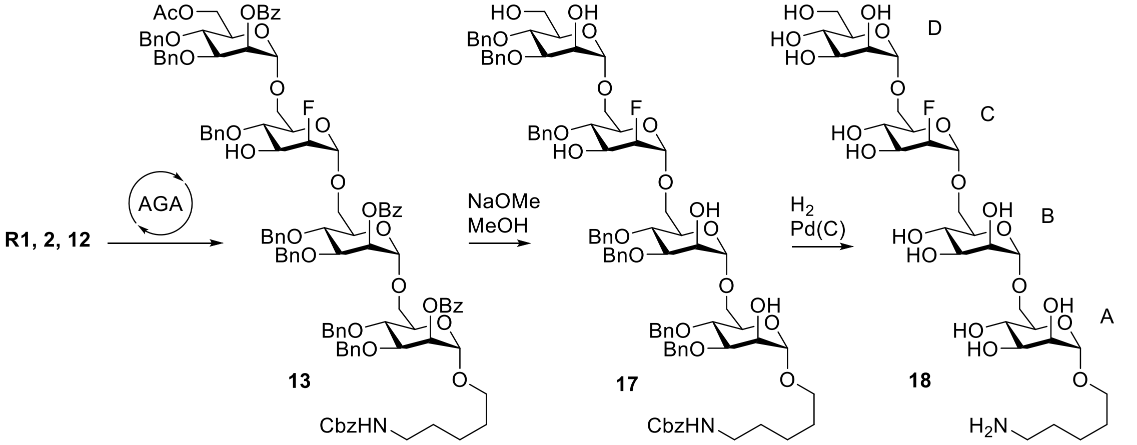

Global deprotection of crude tetramannoside **13** was achieved using modules G and H, and purification with method C afforded tetramannoside **18** (2.3 mg, 13.1 µmol, 25 % based on resin).

Due to the low sample amount and signal overlap, not all ^{13}C resonances of **18** could be detected.
^{1}H NMR (599 MHz, D_2O) δ 8.47 (s, 1H, $HCOO^-$), 5.15 (dd, J_{FH} = 7.6 Hz and J = 1.9 Hz, 1H, H-C1C), 4.94 (d, J = 1.8 Hz, 1H), 4.91 (d, J = 1.8 Hz, 1H), 4.88 (d, J = 1.8 Hz, 1H), 4.86 – 4.84 (m, 1H, H-C2C), 4.82 (d, J = 0.6 Hz, 2H), 4.05 – 3.98 (m, 4H), 3.98 – 3.94 (m, 2H), 3.94 – 3.89 (m, 2H), 3.89 – 3.65 (m, 10H), 3.64 (t, J = 6.5 Hz, 1H), 3.62 – 3.56 (m, 1H), 3.04 – 3.00 (m, 3H), 1.76 – 1.65 (m, 6H), 1.52 – 1.42 (m, 2H) ppm; **^{13}C NMR** (151 MHz, D_2O) δ 171.0 (formeate), 110.0, 99.9, 99.3, 72.7, 70.0, 68.4, 66.6, 66.5, 61.3, 60.8, 39.3, 30.6, 28.0, 22.5 ppm; **^{19}F NMR** (564 MHz, D_2O) δ -205.87 (ddd, J = 49.2, 31.5, 7.5 Hz); **HRMS** (ESI) m/z $[M+H]^+$ calcd for $C_{29}H_{52}FNO_{20}H^+$ 754.3140; found 754.3157; $[M+Na]^+$ calcd for $C_{29}H_{52}FNO_{20}Na^+$ 776.2959; found 776.2979.

Tetramannoside 15

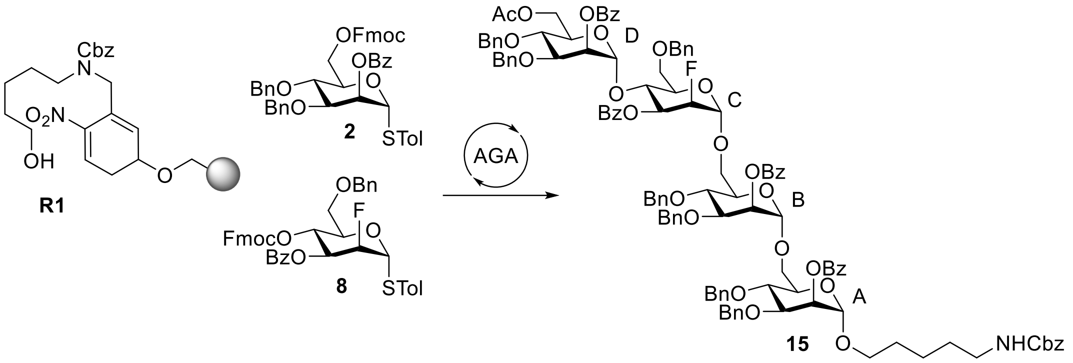

	Module	Conditions
	A: Resin Swelling	
2	B: Acid Wash<br>C: Thioglycoside Glycosylation<br>D: Acidic capping<br>E: Fmoc deprotection	BB **12** (8.0 eq.), 0 °C for 5 min, 20 °C for 20 min
	B: Acid Wash<br>C: Thioglycoside Glycosylation<br>D: Acidic capping<br>E: Fmoc deprotection	BB **4** (8.0 eq.), 0 °C for 5 min, 20 °C for 20 min
	B: Acid Wash<br>C: Thioglycoside Glycosylation<br>D: Acidic capping<br>E: Fmoc deprotection	BB **12** (8.0 eq.), 0 °C for 5 min, 20 °C for 20 min
	D: Acidic capping	

The tetrasaccharide was cleaved from solid support as described in the post-automation manipulations. Removal of excess linker and grease by flash chromatography (Method A) afforded tetrasaccharide **15** as a white semi-solid (14.2 mg, 7.19 µmol, 58 % based on resin).

^{1}H NMR (599 MHz, $CDCl_3$) δ 8.16 – 8.05 (m, 6H, Bz), 7.89 – 7.83 (m, 2H, Bz), 7.59 – 7.53 (m, 1H, Bz), 7.50 – 7.03 (m, 51H, Bz, Bn, Cbz), 5.76 (dd, J = 3.1, 1.9 Hz, 1H, H-C2B), 5.63 (dd, J = 3.3, 1.8 Hz, 1H, H-C2A), 5.51 (ddd, J_{FH} = 28.8 Hz and J = 10.0, 2.5 Hz, 1H, H-C3C), 5.37 (t, J = 2.6 Hz, 1H, H-C2D), 5.22 (q, J = 3.1 Hz, 2H, H-C1C, H-C1D), 5.09 (d, J = 11.7 Hz, 2H, CH_2 Cbz), 5.07 (d, J = 1.8 Hz, 1H, H-C1B), 5.00 (d, J = 11.4 Hz, 1H, CH_2Ph), 4.93 (dt, J_{FH} = 50.1 Hz and J = 2.4 Hz, 1H, H-C2C), 4.94 – 4.89 (m, 2H, H-C1A, CH_2Ph), 4.83 (d, J = 10.9 Hz, 1H, CH_2Ph), 4.80 (d, J = 11.0 Hz, 1H, CH_2Ph), 4.76 (d, J = 11.3 Hz, 1H, CH_2Ph), 4.63 (d, J = 11.4 Hz, 1H, CH_2Ph), 4.62 – 4.54 (m, 3H, CH_2Ph), 4.52 – 4.46 (m, 3H, CH_2Ph), 4.38 (t, J = 9.9 Hz, 1H, H-C4C), 4.29 (d, J = 11.1 Hz, 1H, CH_2Ph), 4.19 (dd, J = 11.7, 3.4 Hz, 1H, H-C6D), 4.16 – 4.11 (m, 2H, H-C6D, CH_2Ph), 4.10 (dd, J = 9.1, 3.4 Hz, 1H, H-C3A), 4.09 – 4.04 (m, 1H, H-C3B), 4.04 (t, J = 9.5 Hz, 1H, H-C4B), 3.96 (dd, J = 11.2, 4.7 Hz, 1H, H-C6A), 3.95 – 3.90 (m, 2H, H-C4A, H-C6B), 3.89-3.84 (m, 2H, H-C5A, H-C3D), 3.82 (t, J = 8.9 Hz, 2H, H-C5B, H-C5C), 3.79 – 3.73 (m, 4H, H-C6A, H-C6C, H-C4D, H-C5D), 3.71 (d, J = 11.6 Hz, 1H, H-C6B), 3.67 (dd, J = 11.0, 1.8 Hz, 2H, H-C6C, OCH_2 linker), 3.46 – 3.40 (m, 1H, OCH_2 linker), 3.28 – 3.10 (m, 2H, NCH_2 linker), 1.99 (s, 3H, Ac), 1.67 – 1.24 (m, 6H, linker) ppm; **^{13}C NMR** (151 MHz, $CDCl_3$) δ 170.7 (C=O, Ac), 166.0, 165.8, 165.6, 164.8 (C=O, Bz), 156.5 (C=O, Cbz), 138.6, 138.5, 138.2, 138.12, 138.10, 137.74, 137.69, 136.8, 133.6, 133.4, 133.3, 133.2, 130.2, 130.1, 130.0, 129.9, 129.7, 129.2, 128.72, 128.68, 128.6, 128.5, 128.44, 128.38, 128.35, 128.3, 128.24, 128.18, 128.1, 128.00, 127.97, 127.9, 127.82, 127.78, 127.75, 127.70, 127.66 (Carom), 100.2 (C1D), 98.1 (C1B), 98.0 (C1A), 97.6 (d, J = 28.7 Hz, C1C), 87.4 (d, J = 178.1 Hz, C2C), 78.8 (C3A), 78.3 (C3B), 77.8 (C3D), 75.3 (CH_2Ph),

75.1 (CH_2Ph), 74.4 (C4A), 73.8 (C4B), 73.7 (C4C), 73.6 (CH_2Ph), 73.3 (C4D), 72.9 (d, J = 17.4 Hz, C3C), 71.8 (CH_2Ph), 71.6 (C5B), 71.5 (CH_2Ph), 71.4 (C5C), 71.1 (CH_2Ph), 70.92 (C5D), 70.85 (C5A), 69.2 (C2A), 68.8 (C2B), 68.7 (C6C), 68.6 (C2D), 67.9 (OCH_2 linker), 66.8 (CH_2 Cbz), 66.5 (C6A), 65.8 (C6B), 63.3 (C6D), 41.1 (NCH_2 linker), 29.8 (linker), 29.2 (linker), 23.6 (linker), 20.9 (Ac) ppm; **^{19}F NMR** (564 MHz, $CDCl_3$) δ -204.40 (ddd, J_{FH} = 49.7, 28.7, 6.9 Hz) ppm; **MALDI-MS** m/z $[M+Na]^+$ calcd for $C_{116}H_{118}FNO_{27}Na^+$ 1998.78; found 1998.71.

Tetramannoside S14

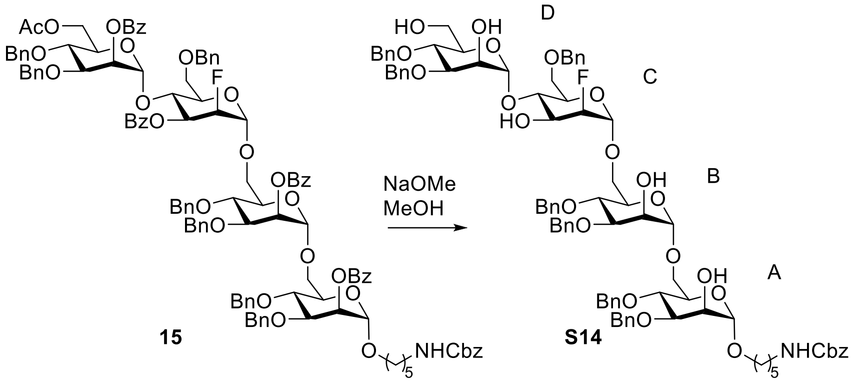

The ester PGs of tetramannoside **15** (14.2 mg, 7.2 µmol, 1.0 eq.) were cleaved using the conditions of module G to give semi-protected tetramannoside **S14** (6.5 mg, 4.3 µmol, 60 %) after purification using method B.
^{1}H NMR (599 MHz, $CDCl_3$) δ 7.39 – 7.19 (m, 40H, Bn, Cbz), 5.33 (s, 1H, H-C1D), 5.07 (s, 2H, CH_2 Cbz), 4.99 (d, J_{FH} = 6.8 Hz, 1H, H-C1C), 4.93 (s, 1H, H-C1B), 4.90 (d, J = 11.2 Hz, 1H, CH_2Ph), 4.87 (d, J = 11.1 Hz, 1H, CH_2Ph), 4.80 (s, 1H, H-C1A), 4.77 (d, J = 11.1 Hz, 1H, CH_2Ph), 4.70 (br d, J_{FH} = 52.0 Hz, 1H, H-C2C), 4.72 – 4.48 (m, 11H, CH_2Ph), 4.10 – 4.08 (m, 1H, H-C2B), 4.07 – 3.99 (m, 2H, H-C3C, H-C2D), 4.01 – 3.98 (m, 1H, H-C2A), 3.96 (t, J = 9.6 Hz, 1H, H-C4C), 3.89 – 3.80 (m, 4H, H-C3A, H-C6A, H-C3B, H-C6B), 3.80 – 3.65 (m, 10H, H-C4A, H-C5A, H-C6A, H-C4B, H-C5B, H-C5C, H-C6C, H-C3D, H-C4D, H-C5D), 3.64 – 3.56 (m, 5H, H-C6B, H-C6C, H-C6D (2x), OCH_2 linker), 3.39 – 3.29 (m, 1H, OCH_2 linker), 3.20 – 3.09 (m, 2H, NCH_2 linker), 1.66 – 1.42 (m, 6H, linker) ppm; **^{13}C NMR** (151 MHz, $CDCl_3$) δ 156.6 (C=O, Cbz), 138.43, 138.41, 138.3, 137.9, 137.8, 136.7, 128.70, 128.66, 128.54, 128.50, 128.4, 128.3, 128.2, 128.12, 128.10, 128.08, 128.06, 128.03, 127.99, 127.86, 127.85, 127.81, 127.78, 127.76, 127.65 (Carom.), 99.7 (C1D), 99.5 (C1B), 99.1 (C1A), 97.6 (d, J = 29.5 Hz, C1C), 90.0 (d, J = 173.1 Hz, C2C), 80.4 (C3A), 80.0 (C3B), 79.5 (C3D), 75.2 (CH_2Ph), 75.1 (CH_2Ph), 74.7 (CH_2Ph), 74.4 (C4A), 74.3 (C4C), 74.1 (C4D), 74.0 (C4B), 73.6 (CH_2Ph), 73.2 (C5D), 72.2 (CH_2Ph), 72.1 (CH_2Ph), 71.9 (CH_2Ph), 71.0 (C5B), 70.79 (C5A), 70.75 (C5C), 70.7 (m, C3C), 68.9 (C2D), 68.8 (C6C), 68.4 (C2A), 68.1 (C2B), 67.6 (OCH_2 linker), 66.9 (C6B), 66.8 (CH_2 Cbz), 66.7 (C6A), 61.7 (C6D), 41.1 (NCH_2 linker), 29.8 (linker), 29.1 (linker), 23.6 (linker) ppm; **^{19}F NMR** (564 MHz, $CDCl_3$) δ -206.31 (br dd, J_{FH} = 50.0, 29.0 Hz) ppm. **MALDI-MS** m/z $[M+Na]^+$ calcd for $C_{86}H_{100}FNO_{22}Na^+$ 1540.66; found 1540.81.

Tetramannoside S15

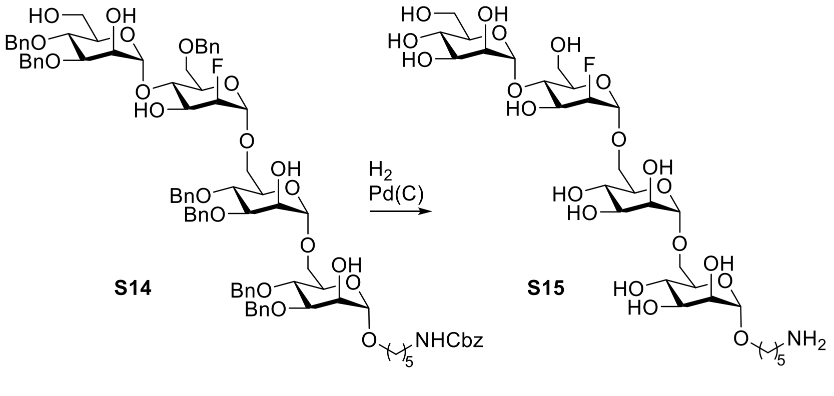

Global deprotection of tetramannoside **S14** was achieved using module H.
^{19}F NMR (376 MHz, D_2O) δ -205.50 ppm.
HRMS (ESI) m/z $[M+H]^+$ calcd for $C_{29}H_{52}FNO_{20}H^+$ 754.3140; found 754.3149.

Trimannoside 16

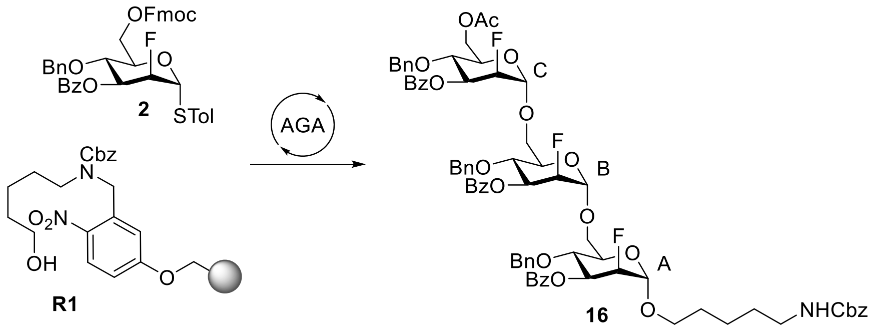

	Module	Conditions
	A: Resin Swelling	
3	B: Acid Wash	
	C: Thioglycoside Glycosylation	BB **2** (8.0 eq.), 0 °C for 5 min, 20 °C for 20 min
	D: Acidic capping	
	E: Fmoc deprotection	
	D: Acidic capping	

Trisaccharide **16** was cleaved from solid support as described in the post-automation manipulations. Removal of excess linker and grease by flash chromatography (Method A) afforded trisaccharide **16** as a white semi-solid (8.7 mg, 6.4 µmol, 51 % based on resin).

^{1}H NMR (599 MHz, $CDCl_3$) δ 8.16 – 8.06 (m, 6H, Bz), 7.64 – 7.55 (m, 3H, Bz), 7.51 – 7.42 (m, 7H, Bz, Cbz), 7.38 – 7.15 (m, 19H, Cbz, Bn), 5.64 (ddd, J_{FH} = 29.7 Hz and J = 9.7, 2.5 Hz, 1H, H-C3C), 5.57 (ddd, J_{FH} = 29.2 Hz and J = 9.7, 2.6 Hz, 1H, H-C3B), 5.53 (ddd, J_{FH} = 29.3 Hz and J = 9.7, 2.5 Hz, 1H, H-C3A), 5.20 (dd, J_{FH} = 7.0 Hz and J = 1.9 Hz, 1H, H-C1C), 5.11 (d, J_{FH} = 7.9 Hz, 1H, H-C1B), 5.06 (s, 2H, CH_2 Cbz), 4.98 (dt, J_{FH} = 50.0 Hz and J = 2.2 Hz, 1H, H-C2C), 4.93 (d, J_{FH} = 7.1 Hz, 1H, H-C1A), 4.92 (dt, J_{FH} = 50.3 Hz and J = 1.6 Hz, 1H, H-C2B), 4.83 (dt, J_{FH} = 50.3 Hz and J = 2.3 Hz, 1H, H-C2A), 4.77 (d, J = 11.1 Hz, 1H, CH_2Ph), 4.76 (d, J = 11.1 Hz, 1H, CH_2Ph), 4.71 (d, J = 10.9 Hz, 1H, CH_2Ph), 4.63 (d, J = 11.2 Hz, 1H, CH_2Ph), 4.63 (d, J = 11.1 Hz, 1H, CH_2Ph), 4.58 (d, J = 10.9 Hz, 1H, CH_2Ph), 4.39 (dd, J = 12.1, 2.2 Hz, 1H, H-C6C), 4.29 (dd, J = 12.1, 4.3 Hz, 1H, H-C6C), 4.13 (t, J = 9.9 Hz, 1H, H-C4B), 4.11 (t, J = 10.0 Hz, 1H, H-C4A), 4.10 (t, J = 9.9 Hz, 1H, H-C4C), 3.99 (dt, J = 10.1, 3.2 Hz, 1H, H-C5C), 3.99 – 3.92 (m, 2H, H-C5B, H-C6A), 3.90 (dd, J = 11.8, 4.3 Hz, 1H, H-C6B), 3.84 – 3.78 (m, 2H, H-C6A, H-C6B), 3.76 (q, J = 7.1 Hz, 1H, OCH_2 linker), 3.49 – 3.42 (m, 1H, OCH_2 linker), 3.25 – 3.13 (m, 2H, NCH_2 linker), 2.09 (s, 3H, Ac), 1.67 – 1.50 (m, 4H, linker), 1.45 – 1.36 (m, 2H, linker) ppm; **^{13}C NMR** (151 MHz, $CDCl_3$) δ 170.9 (C=O, Ac), 165.9, 165.8, 165.7 (C=O, Bz), 156.6 (C=O, Cbz), 137.7, 137.6, 137.4, 136.9, 133.58, 133.55, 133.5, 130.1, 130.03, 129.97, 129.8, 129.73, 129.68, 128.73, 128.69, 128.67, 128.64, 128.59, 128.57, 128.5, 128.3, 128.20, 128.17, 128.10, 128.08, 128.06, 127.99 (Carom), 97.8 (d, J = 29.0 Hz, C1C), 97.5 (d, J = 29.0 Hz, C1B), 97.2 (d, J = 28.6 Hz, C1A), 87.8 (d, J = 177.4 Hz, C2A), 87.64 (d, J = 177.4 Hz, C2C), 87.58 (d, J = 177.2 Hz, C2B), 75.4, 75.3 (CH_2Ph), 73.6 (d, J = 16.8 Hz, C3A), 73.33 (d, J = 17.0 Hz, C3B), 73.25 (d, J = 16.7 Hz, C3C), 73.2 (C4A), 72.9 (C4B, C4C), 71.61 (C5B), 71.58 (C5A), 70.1 (C5C), 68.3 (OCH_2 linker), 66.6 (CH_2 Cbz), 66.2 (C6A), 65.9 (C6B), 62.9 (C6C), 41.1 (NCH_2 linker), 29.8 (linker), 29.0 (linker), 23.6 (linker), 21.0 (Ac) ppm; **^{19}F NMR** (564 MHz, $CDCl_3$) δ -204.22 (ddd, J_{FH} = 50.2, 29.4, 7.2 Hz, 1F), -204.29 (ddd, J_{FH} = 50.4, 29.4, 7.4 Hz, 1F), -204.35 (ddd, J_{FH} = 50.1, 29.6, 7.1 Hz, 1F) ppm; **HRMS** (ESI) m/z $[M+Na]^+$ calcd for $C_{75}H_{78}F_3NO_{19}Na^+$ 1376.5012; found 1376.5007.

Trimannoside **S16**

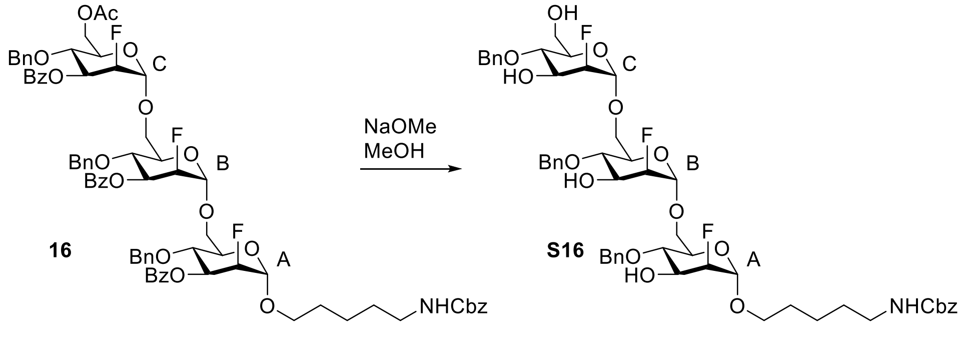

The ester PGs of trimannoside **16** (8.7 mg, 6.4 µmol, 1.0 eq.) were cleaved using the conditions of module G to give semi-protected tetramannoside **S16** (4.0 mg, 4.0 µmol, 63% (32 % from resin)) as a white semi-solid.

^{1}H NMR (599 MHz, $CDCl_3$) δ 7.38 – 7.27 (m, 20H, Bn, Cbz), 5.07 (s, 2H, CH_2 Cbz), 5.05 (d, J_{FH} = 7.6 Hz, 1H, H-C1C), 5.01 (d, J_{FH} = 7.1 Hz, 1H, H-C1B), 4.90 (d, J = 11.4 Hz, 1H, CH_2Ph), 4.88 (d, J_{FH} = 8.0 Hz, 1H, H-C1A), 4.85 (d, J = 11.4 Hz, 1H, CH_2Ph), 4.83 (d, J = 11.3 Hz, 1H, CH_2Ph), 4.71 (d, J = 11.2 Hz, 1H, CH_2Ph), 4.69 (dt, J_{FH} = 49.8 Hz and J = 2.1 Hz, 1H, H-C2C), 4.68 (dt, J_{FH} = 51.6 Hz and J = 2.4 Hz, 1H, H-C2B), 4.63 (dt, J_{FH} = 49.8 Hz and J = 2.1 Hz, 1H, H-C2A), 4.63 (d, J = 11.3 Hz, 1H, CH_2Ph), 4.59 (d, J = 11.2 Hz, 1H, CH_2Ph), 4.03 – 3.93 (m, 3H, H-C3A, H-C3B, H-C3C), 3.86 (dd, J = 11.3, 5.6 Hz, 1H, H-C6A), 3.83 (dd, J = 11.6, 3.9 Hz, 1H, H-C6B), 3.81 – 3.77 (m, 2H, H-C5B, H-C6C), 3.76 – 3.69 (m, 4H, H-C5A, H-C6A, H-C4C, H-C6C), 3.69 – 3.60 (m, 4H, H-C4B, H-C6B, H-C5C, OCH_2 linker), 3.57 (t, J = 9.5 Hz, 1H, H-C4A), 3.44 – 3.36 (m, 1H, OCH_2 linker), 3.18 – 3.10 (m, 2H, NCH_2 linker), 1.66 – 1.44 (m, 6H, linker) ppm; **^{13}C NMR** (151 MHz, $CDCl_3$) δ 156.7 (C=O, Cbz), 138.2, 138.2, 138.0, 136.6, 128.7, 128.7, 128.7, 128.6, 128.3, 128.2, 128.1, 127.9, 127.9 (Carom), 97.7 (d, J = 29.5 Hz, C1C), 97.4 (d, J = 29.5 Hz, C1B), 97.0 (d, J = 29.5 Hz, C1A), 90.0 (d, J = 173.4 Hz, C2A), 89.8 (d, J = 173.7 Hz, C2B), 89.7 (d, J = 174.0 Hz, C2C), 76.1 (C4A), 75.6 (C4B, C4C), 75.3 (CH_2Ph), 75.2 (2 x CH_2Ph), 72.0 (C5C), 71.6 (d, J = 17.8 Hz, C3A), 71.5 (br, C3B), 71.2 (d, J = 18.2 Hz, C3C), 71.1 (C5B), 71.0 (C5A), 68.1 (OCH_2 linker), 66.9 (CH_2 Cbz), 66.6 (C6A), 66.3 (C6B), 61.9 (C6C), 41.1 (NCH_2 linker), 29.8 (linker), 29.0 (linker), 24.9 (linker) ppm. **^{19}F NMR** (564 MHz, $CDCl_3$) δ -206.27 (m, 1F), -206.33 (m, 1F), -206.33 (m, 1F) ppm; **HRMS** (ESI) m/z $[M+Na]^+$ calcd for $C_{52}H_{64}F_3NO_{15}Na^+$ 1022.4120; found 1022.4122.

Trimannoside **S17**

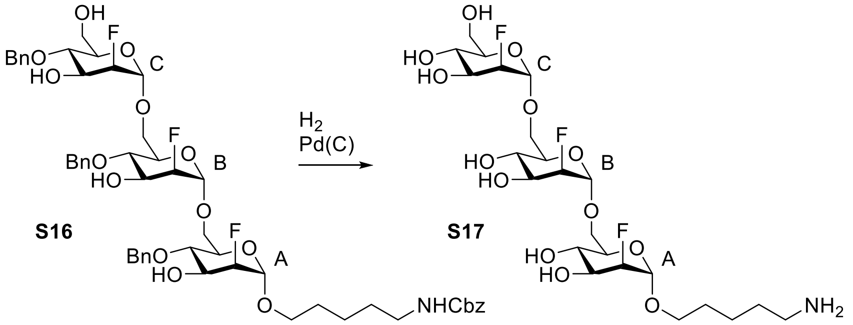

Global deprotection of trimannoside **S16** was achieved using module H.
^{19}F NMR (376 MHz, MeOD) δ -206.66, -206.75, -206.91 ppm.
HRMS (ESI) m/z $[M+H]^+$ calcd for $C_{23}H_{40}F_3NO_{13}H^+$ 596.2525; found 596.2525.

Pentamannoside 20

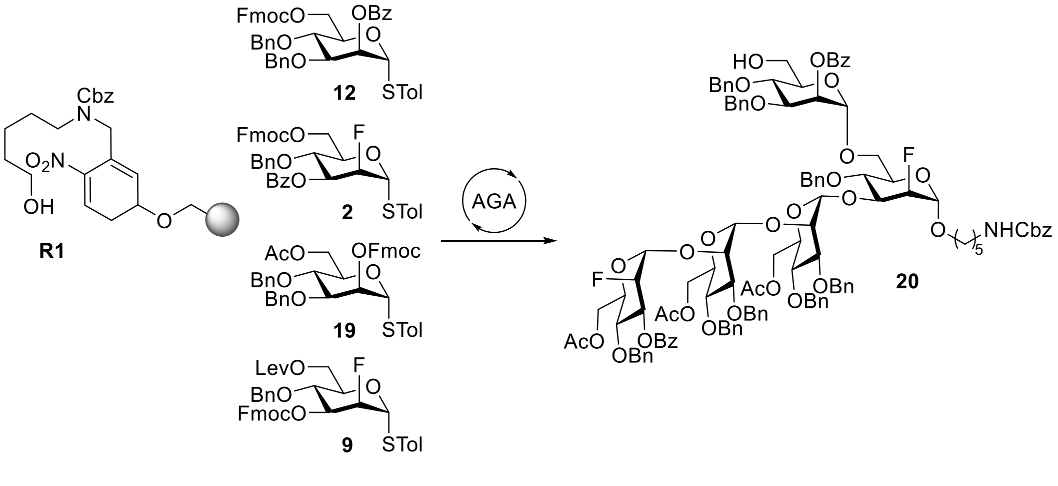

	Module	Conditions
	A: Resin Swelling	
	B: Acid Wash	
	C: Thioglycoside Glycosylation	BB **9** (8.0 eq.), -15 °C for 20 min, 0 °C for 0 min
	D: Acidic capping	
	E: Fmoc deprotection	
2	B: Acid Wash	
	C: Thioglycoside Glycosylation	BB **19** (5.0 eq.), 0-25°C for 15 min, -15 °C for 15 min
	C: Thioglycoside Glycosylation	BB **19** (5.0 eq.), 0-25°C for 15 min, -15 °C for 15 min
	D: Acidic capping	
	E: Fmoc deprotection	
	B: Acid Wash	
	C: Thioglycoside Glycosylation	BB **2** (8.0 eq.), 0 °C for 20 min, 20 °C for 10 min
	D: Acidic capping	
	E: Fmoc deprotection	
	B: Acid Wash	
	C: Thioglycoside Glycosylation	BB **12** (5.0 eq.), 0 °C for 20 min, 20 °C for 15 min
	C: Thioglycoside Glycosylation	BB **12** (5.0 eq.), 0 °C for 20 min, 20 °C for 15 min
	D: Acidic capping	
	E: Fmoc deprotection	

The pentasaccharide was cleaved from solid support as described in the post-automation manipulations to yield the crude pentasaccharide (10.4 mg, 4.9 µmol, 39%).
^{19}F NMR (376 MHz, $CDCl_3$) δ -203.43 ppm.
MALDI-MS m/z $[M+Na]^+$ calcd for $C_{119}H_{129}F_2NO_{31}Na^+$ 2128.84; found 2129.01

Crude MALDI-MS:

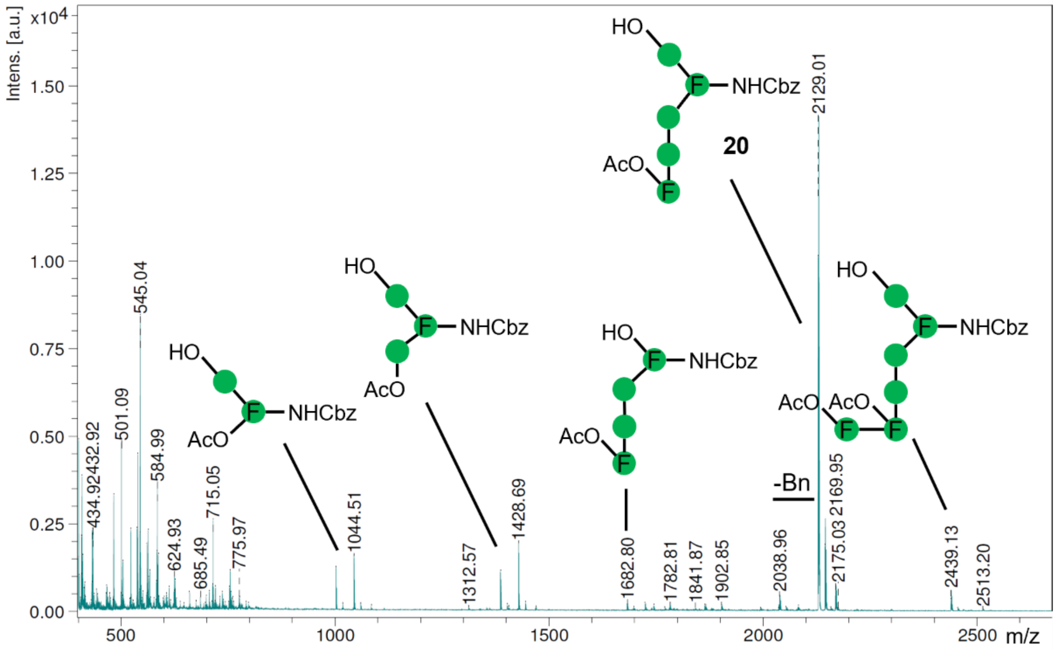

References

[1] C.S. Teschers, R. Gilmour, *Org. Process Res. Dev.* **2020**, *24*, 2234-2239.
[2] C. Bucher, R. Gilmour, *Angew. Chem. Int. Ed.* **2010**, *49*, 8724-8726.
[3] D. A. Evans, W. C. Black, *J. Am. Chem. Soc.* **1993**, *115*, 4497-4513.
[4] M. Boultadakis-Arapinis, P. Lemoine, S. Turcaud, L. Micouin, T. Lecourt, *J. Am. Chem. Soc.* **2010**, *132*, 15477-15479.
[5] C.J. Crawford, Y. Qiao, Y. Liu, D. Huang, W. Yan, P.H. Seeberger, S. Oscarson, S. Chen, *Org. Process Res. Dev.* **2021**, *25*, 1573-1578.
[6] A. A. Joseph, A. Pardo-Vargas, P. H. Seeberger*J. Am. Chem. Soc.* 2020, 142, 19, 8561–8564

NMR Spectra

^{1}H NMR spectrum (400 MHz, $CDCl_3$) of compound **S2**

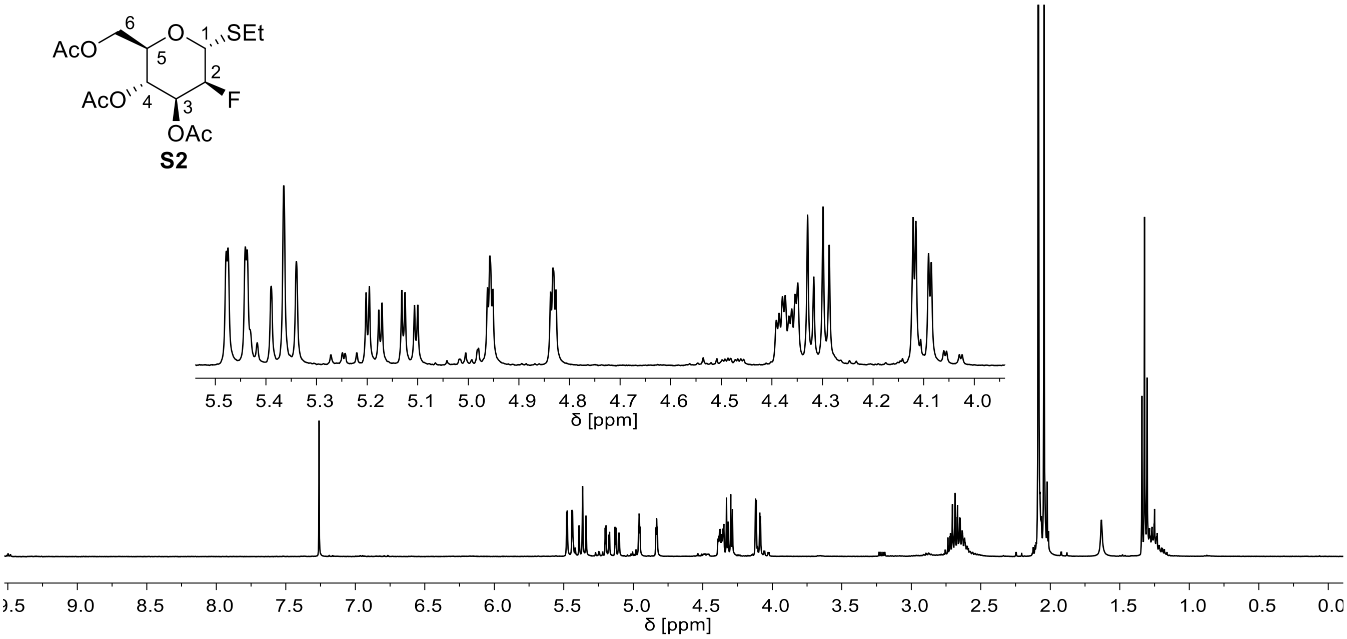

^{19}F NMR spectrum (376 MHz, $CDCl_3$) of compound **S2**

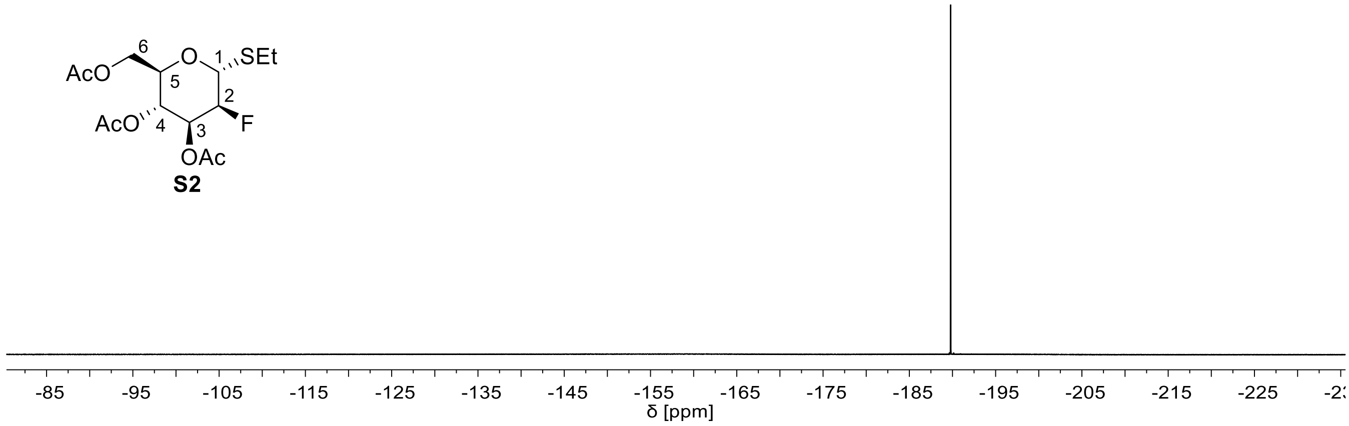

^{1}H NMR spectrum (400 MHz, CD_2Cl_2) of compound **S3**

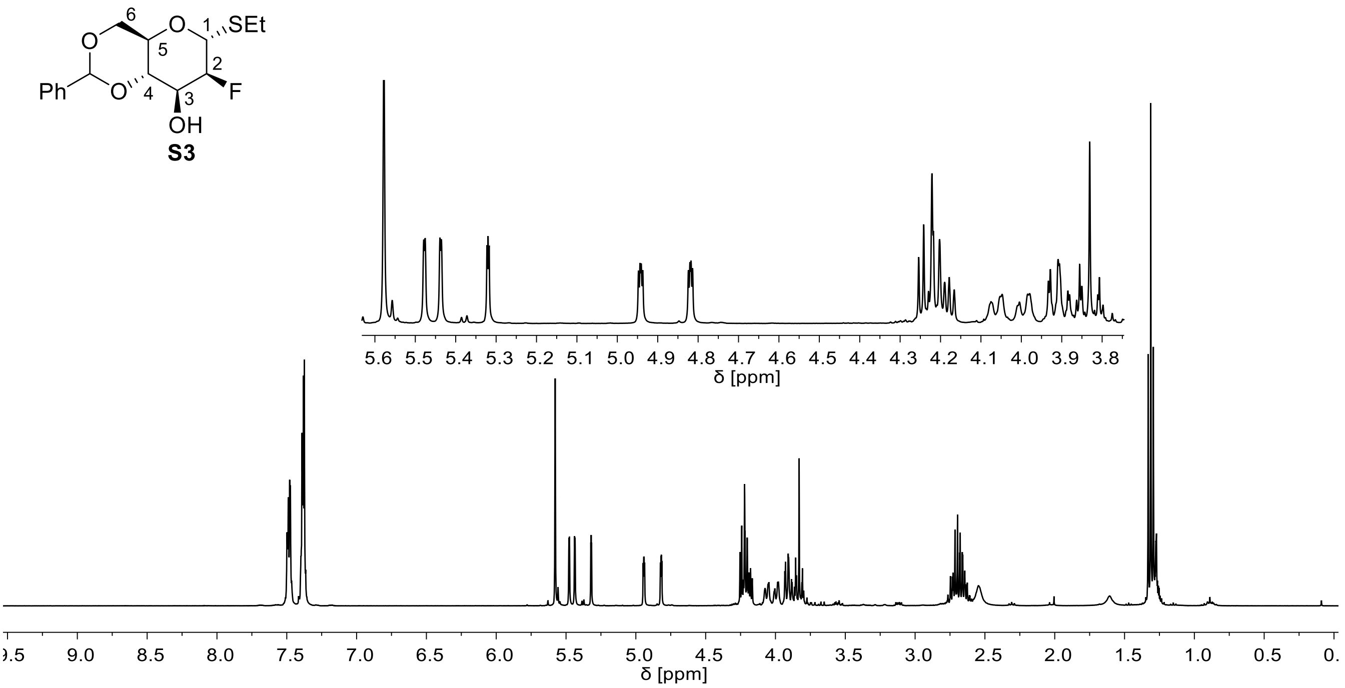

^{19}F NMR spectrum (376 MHz, CD_2Cl_2) of compound **S3**

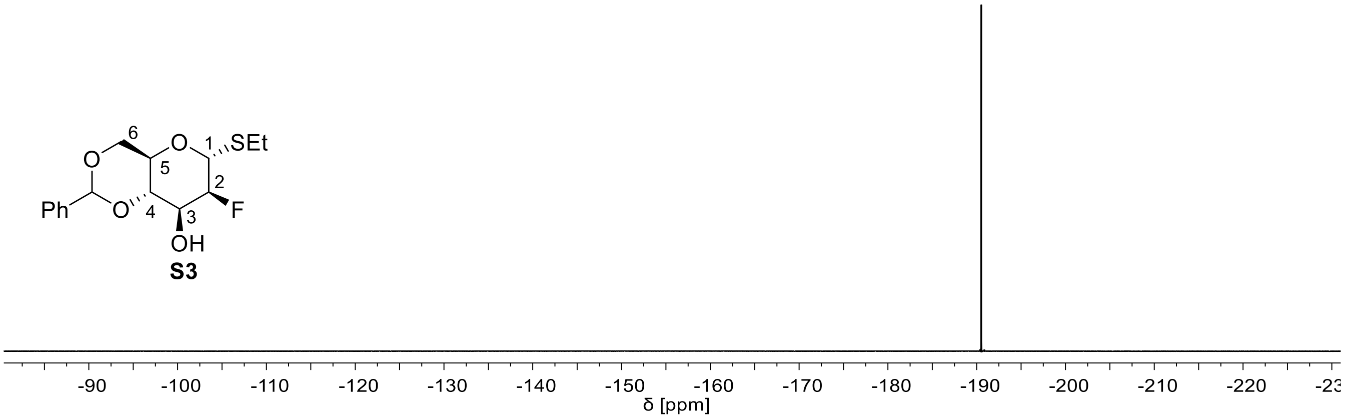

^{1}H NMR spectrum (599 MHz, CD_2Cl_2) of compound **S4**

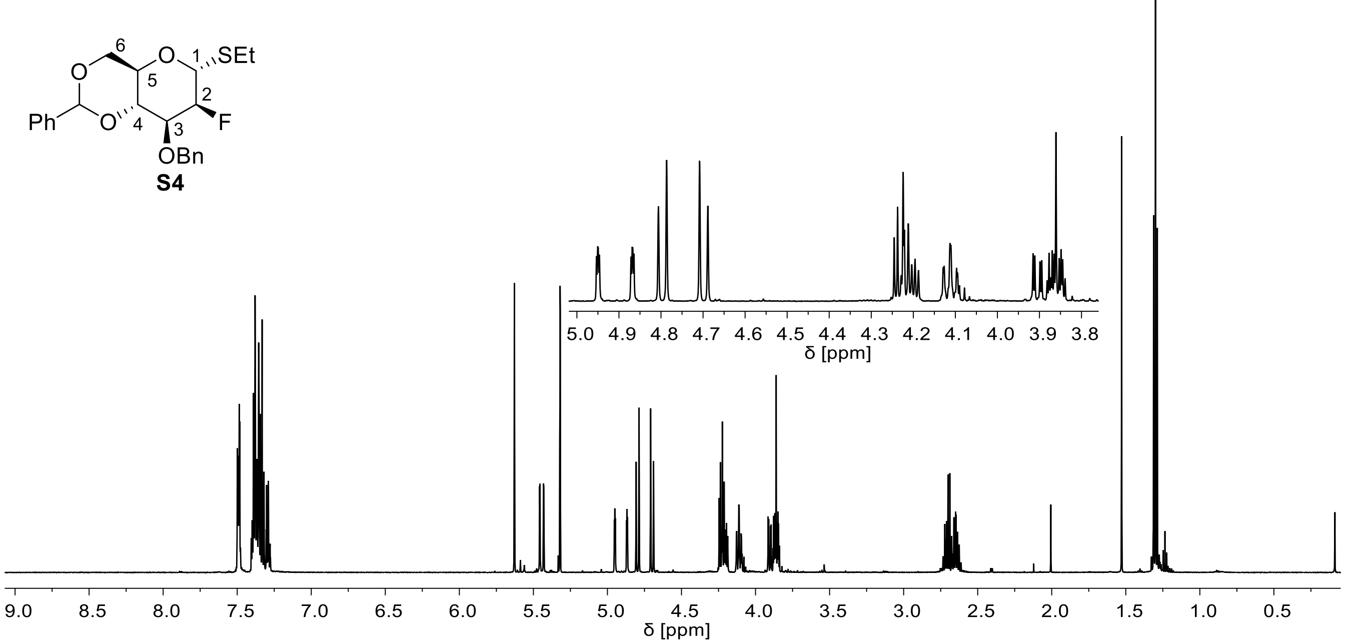

^{13}C NMR spectrum (151 MHz, CD_2Cl_2) of compound **S4**

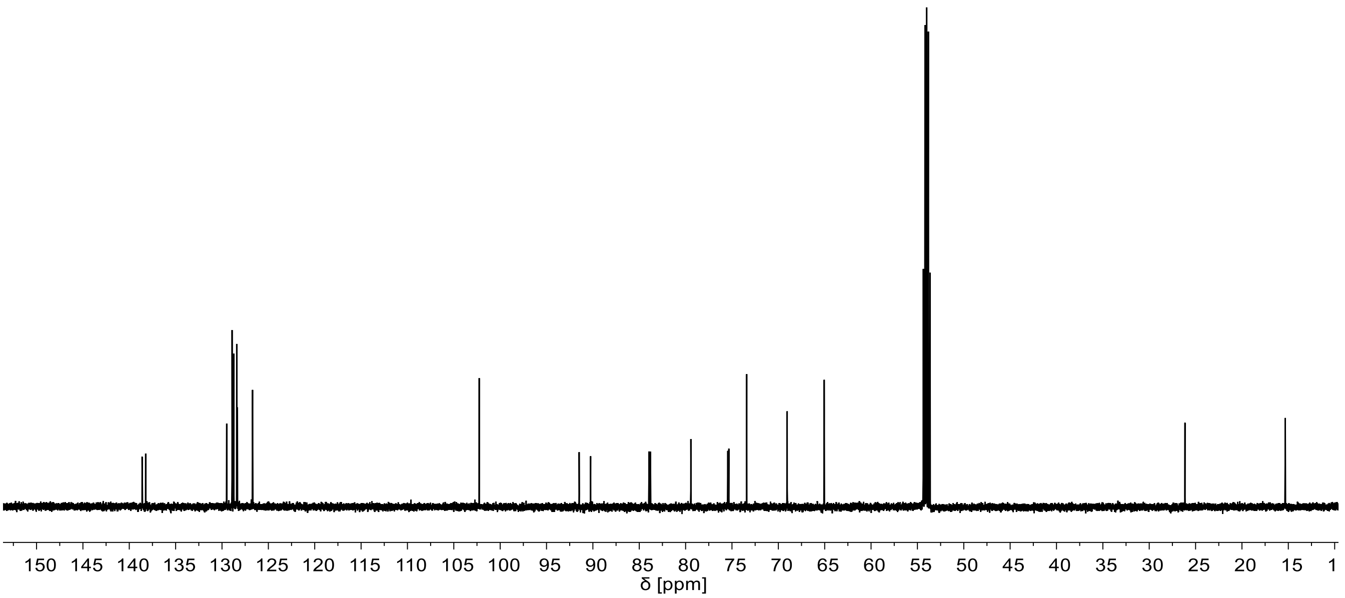

^{19}F NMR spectrum (564 MHz, CD_2Cl_2) of compound **S4**

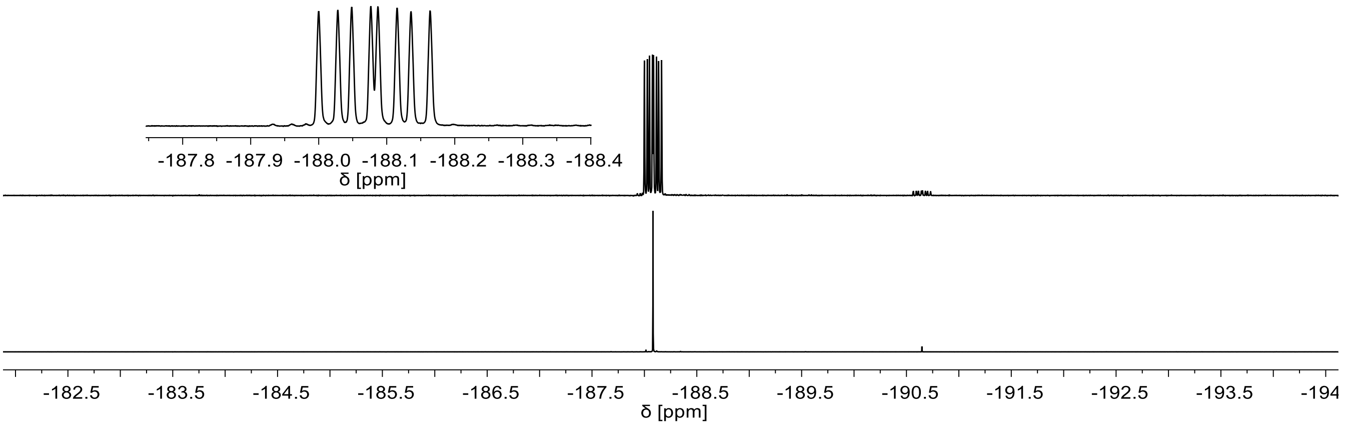

^{1}H NMR spectrum (599 MHz, $CDCl_3$) of compound **S5**

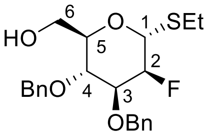

S5

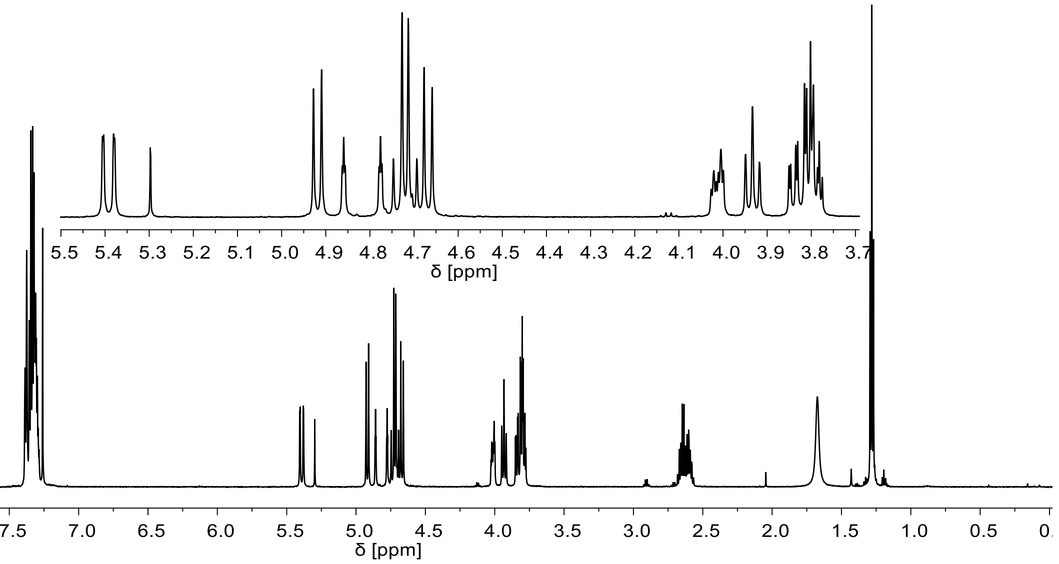

^{13}C NMR spectrum (151 MHz, $CDCl_3$) of compound **S5**

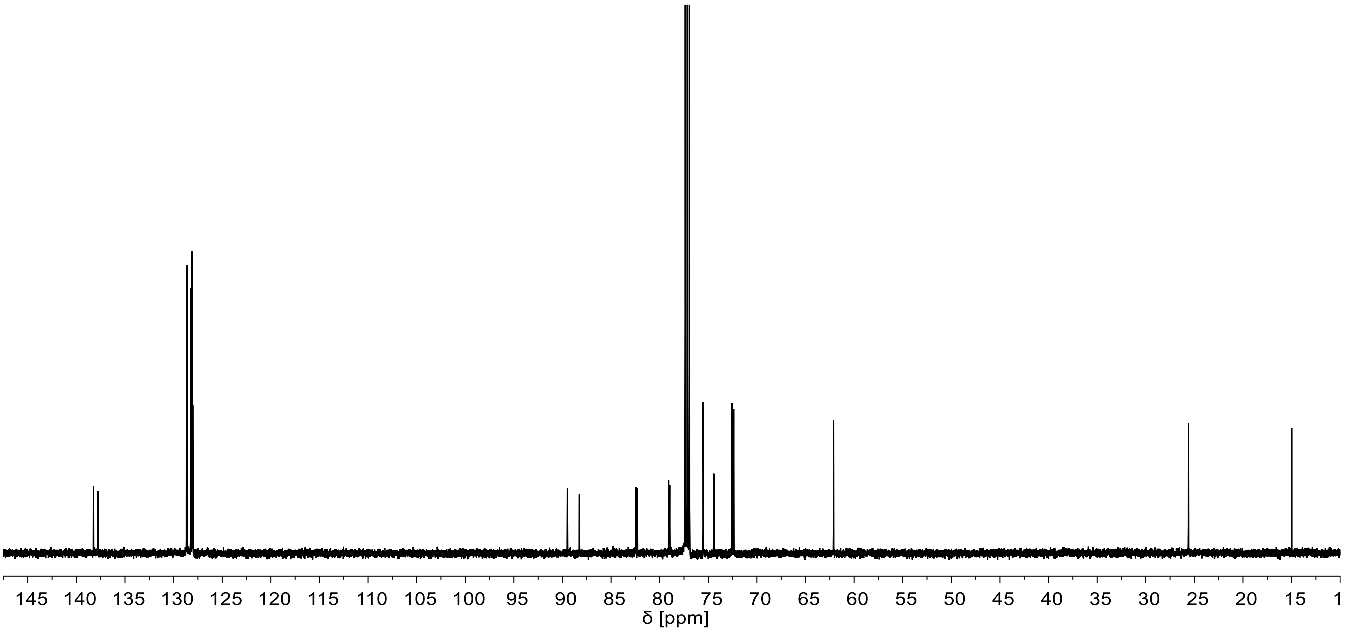

^{19}F NMR spectrum (564 MHz, $CDCl_3$) of compound **S5**

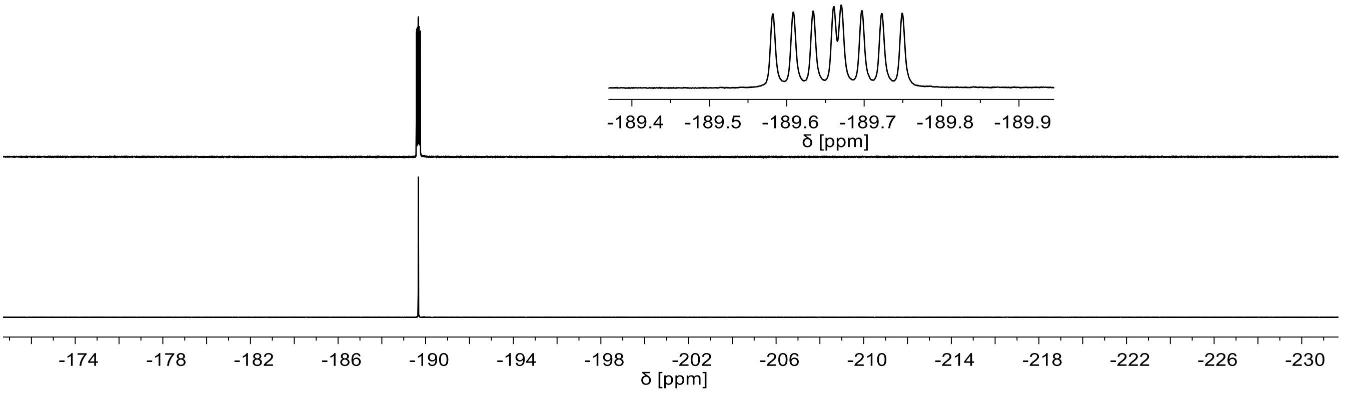

SUPPORTING INFORMATION

^{1}H NMR spectrum (599 MHz, $CDCl_3$) of compound **1**

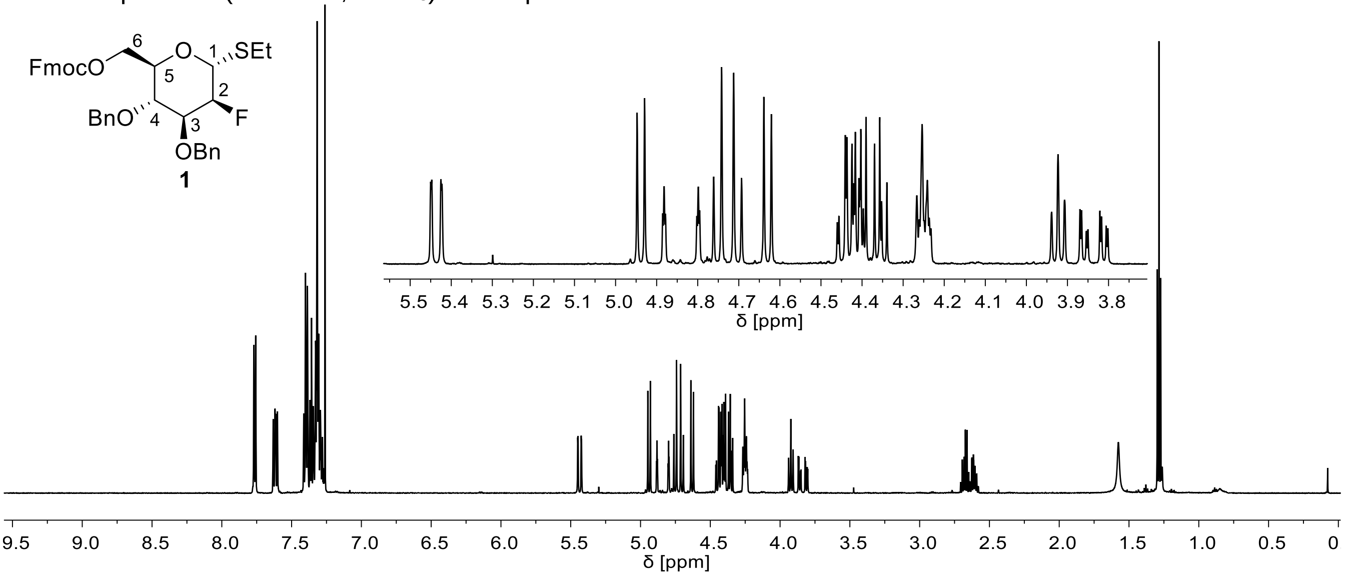

^{13}C NMR spectrum (151 MHz, $CDCl_3$) of compound **1**

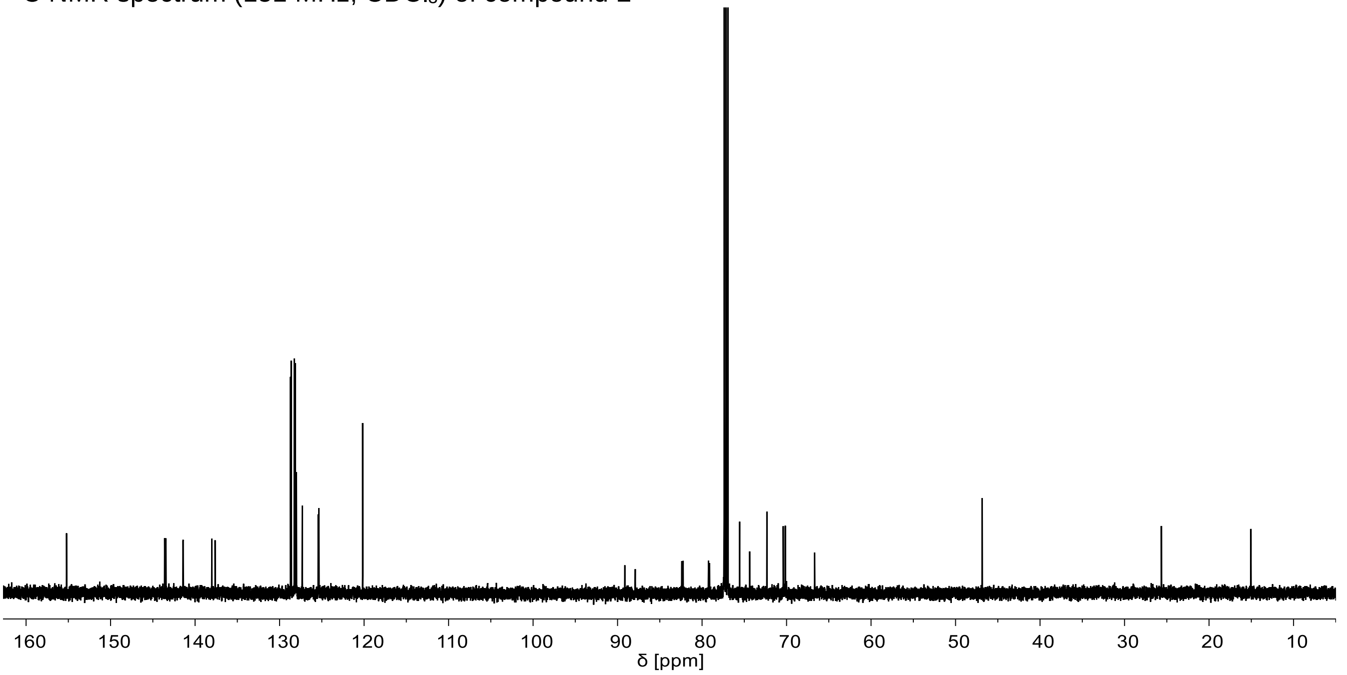

^{19}F NMR spectrum (564 MHz, $CDCl_3$) of compound **1**

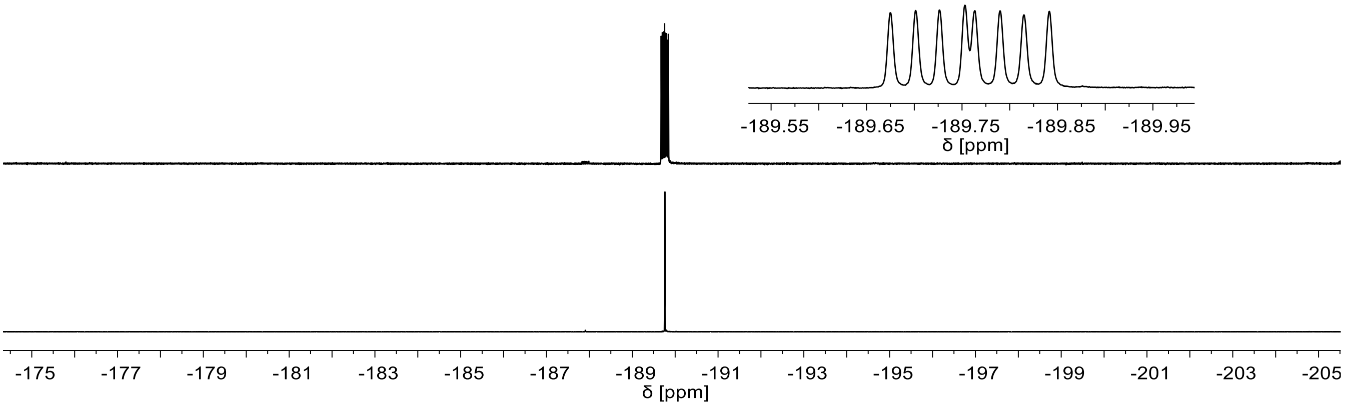

^{1}H NMR spectrum (599 MHz, $CDCl_3$) of compound **S6**

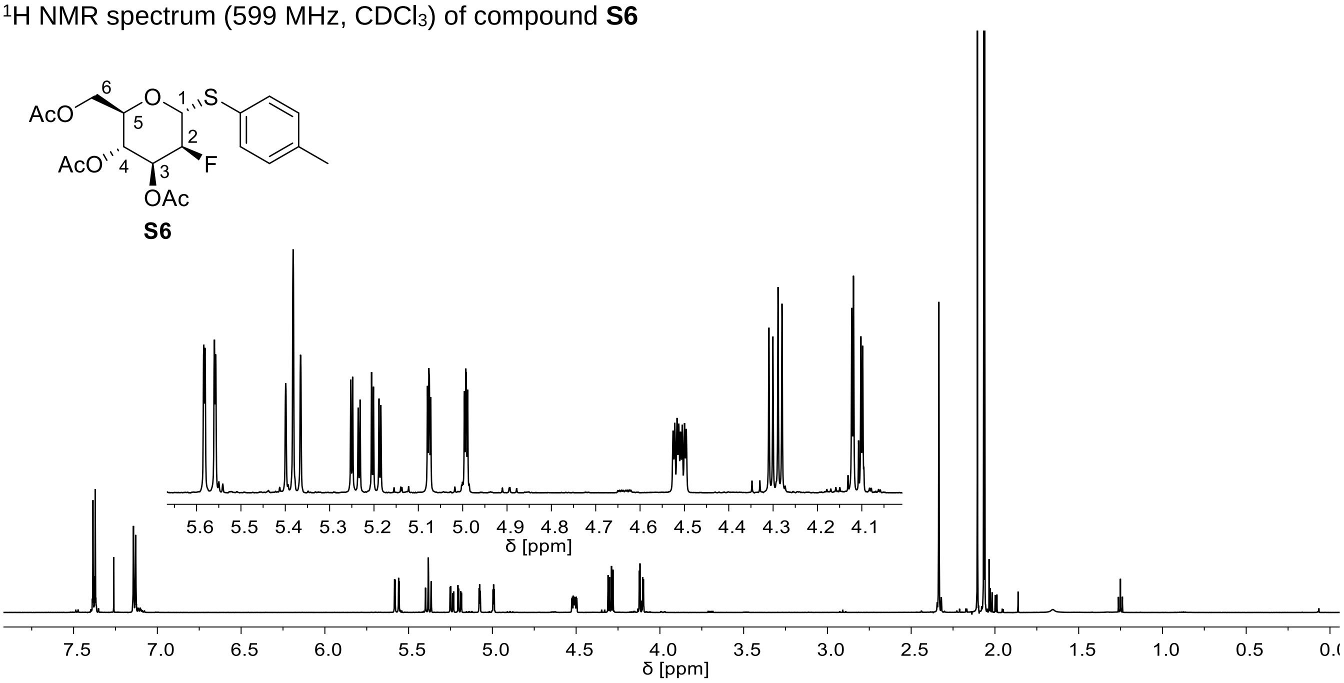

^{13}C NMR spectrum (151 MHz, $CDCl_3$) of compound **S6**

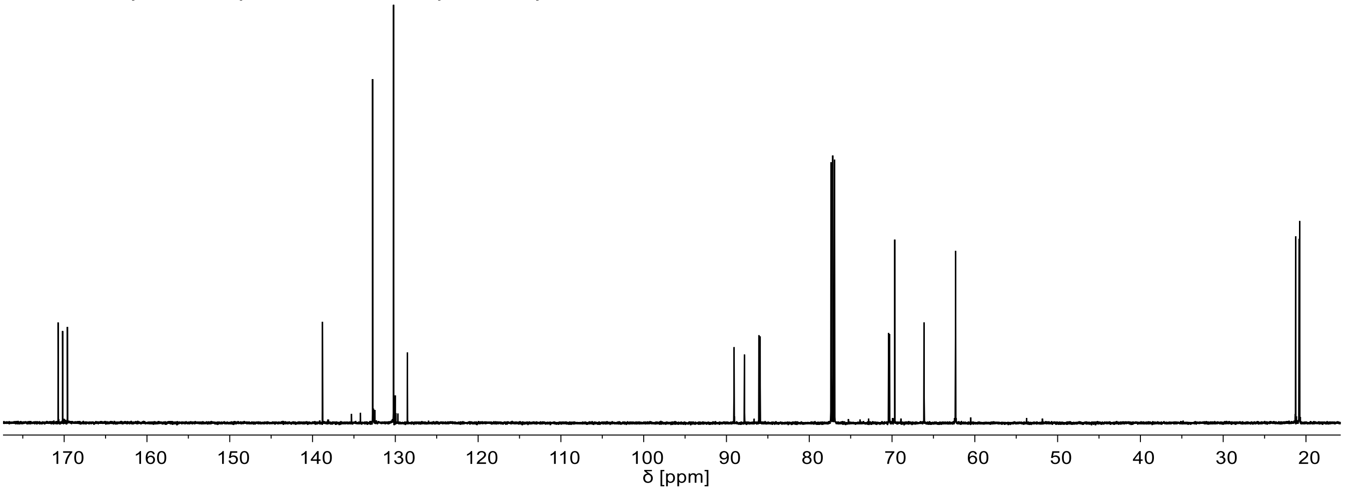

^{19}F NMR spectrum (564 MHz, $CDCl_3$) of compound **S6**

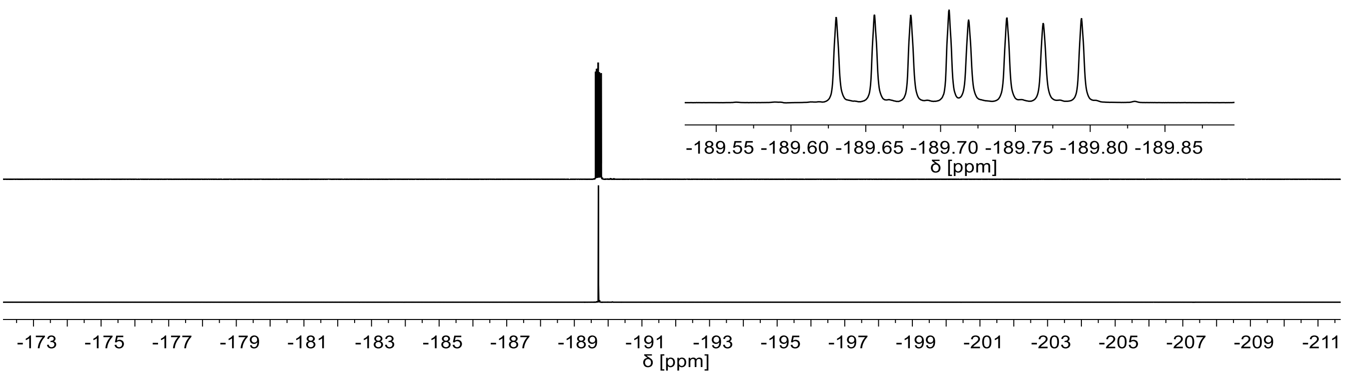

^{1}H NMR spectrum (599 MHz, CD_2Cl_2) of compound **S7**

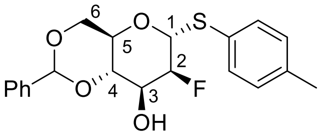

S7

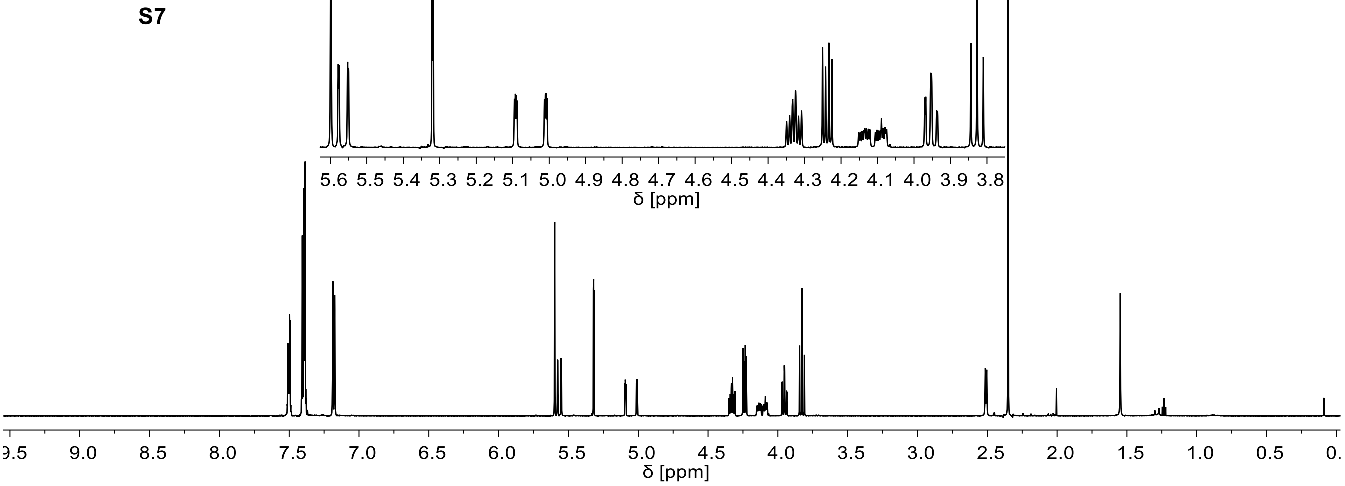

^{13}C NMR spectrum (151 MHz, CD_2Cl_2) of compound **S7**

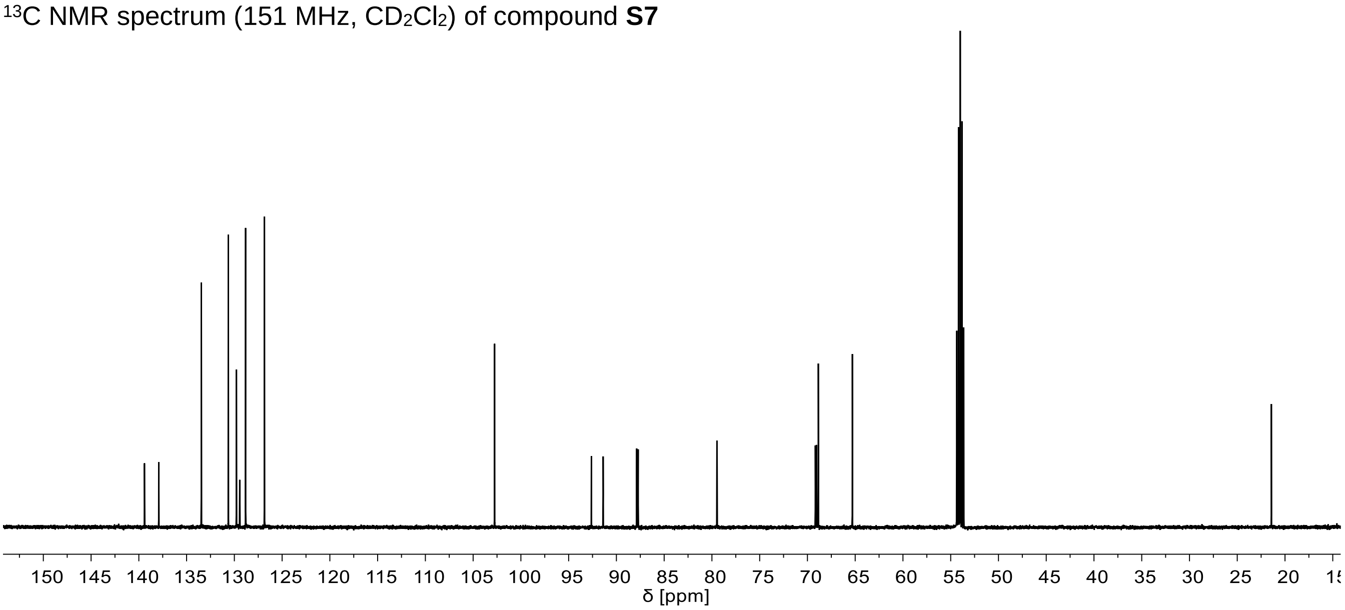

^{19}F NMR spectrum (564 MHz, CD_2Cl_2) of compound **S7**

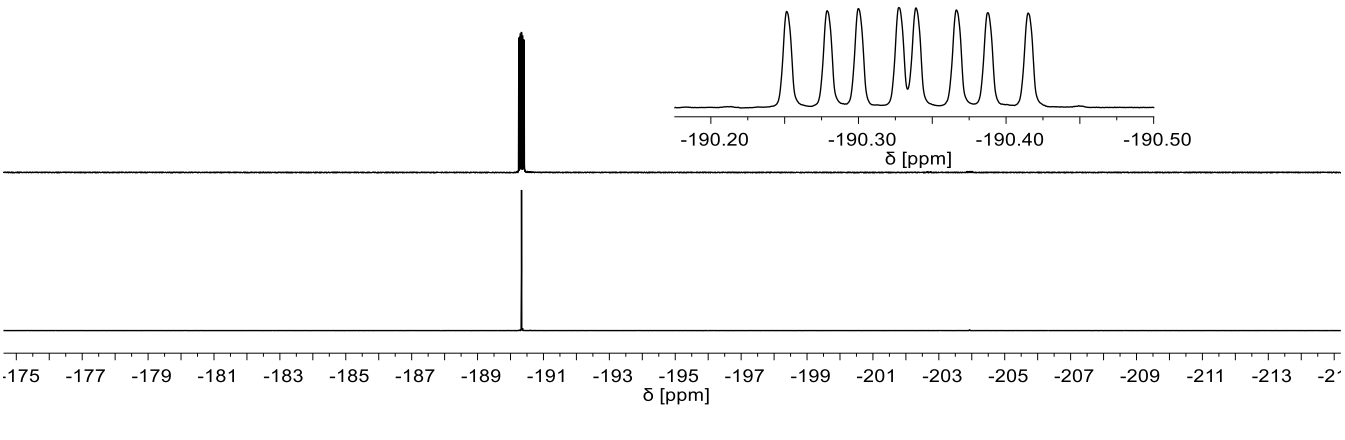

^{1}H NMR spectrum (599 MHz, $CDCl_3$) of compound **S8**

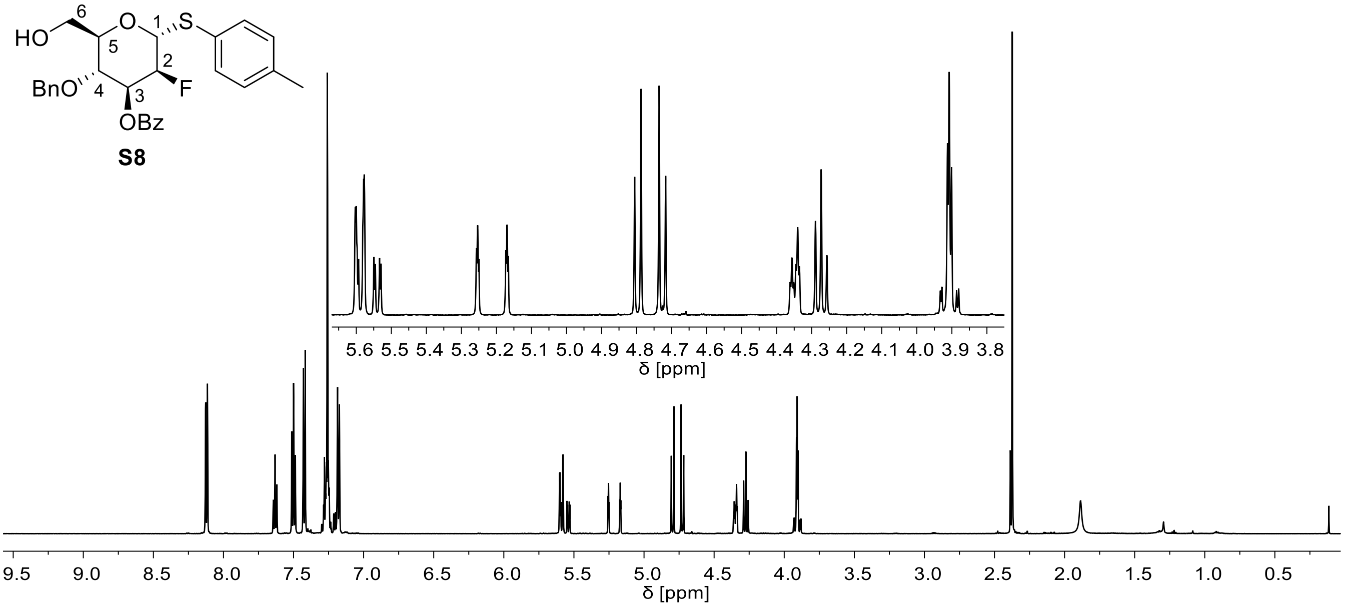

S8

^{13}C NMR spectrum (151 MHz, $CDCl_3$) of compound **S8**

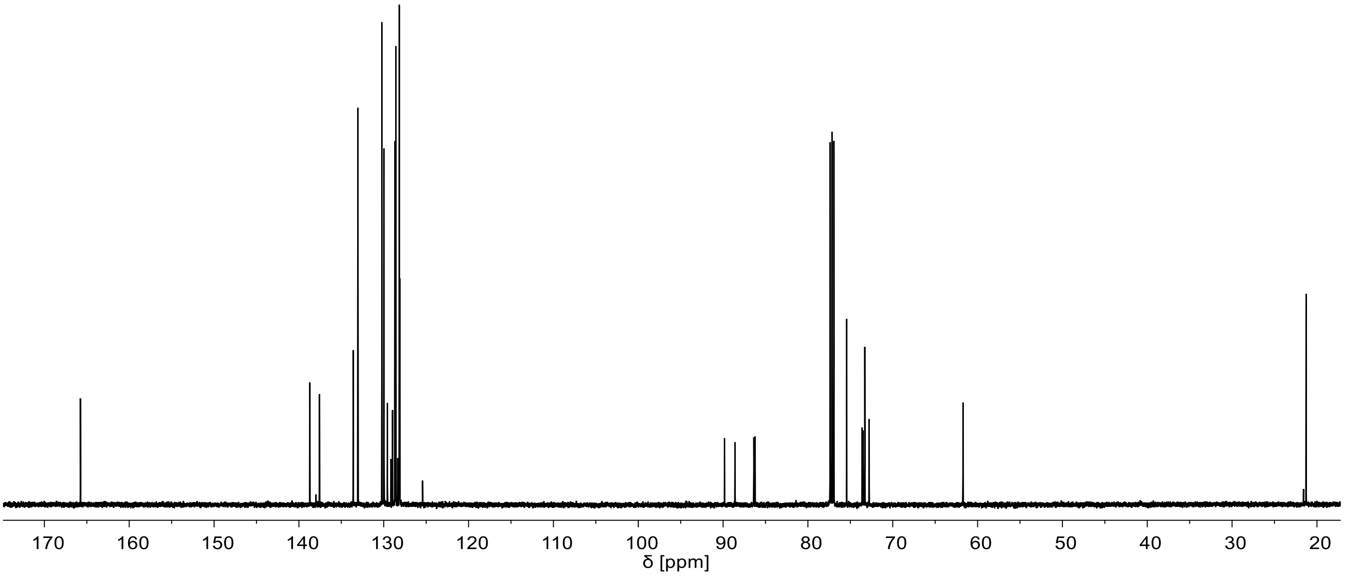

^{19}F NMR spectrum (564 MHz, $CDCl_3$) of compound **S8**

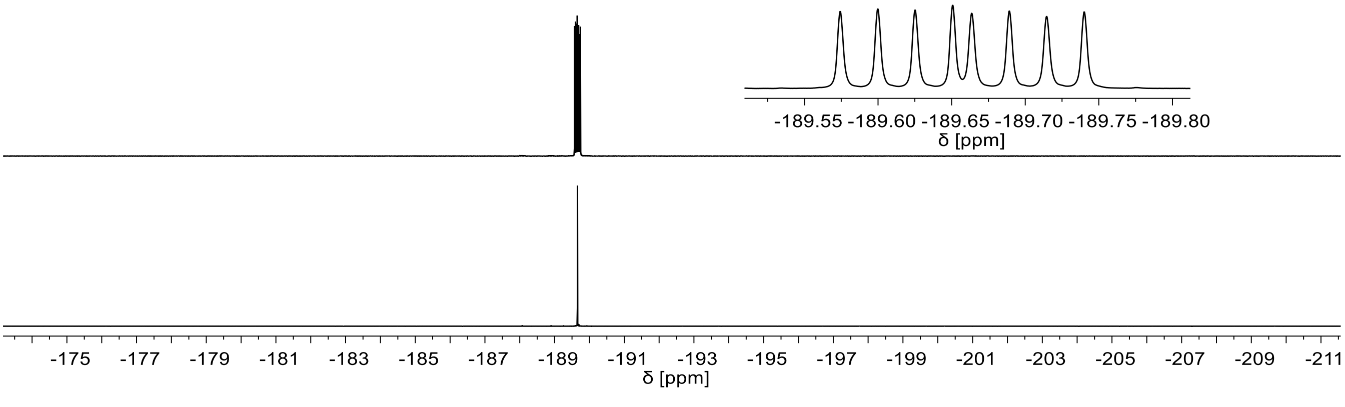

^{1}H NMR spectrum (599 MHz, $CDCl_3$) of compound **2**

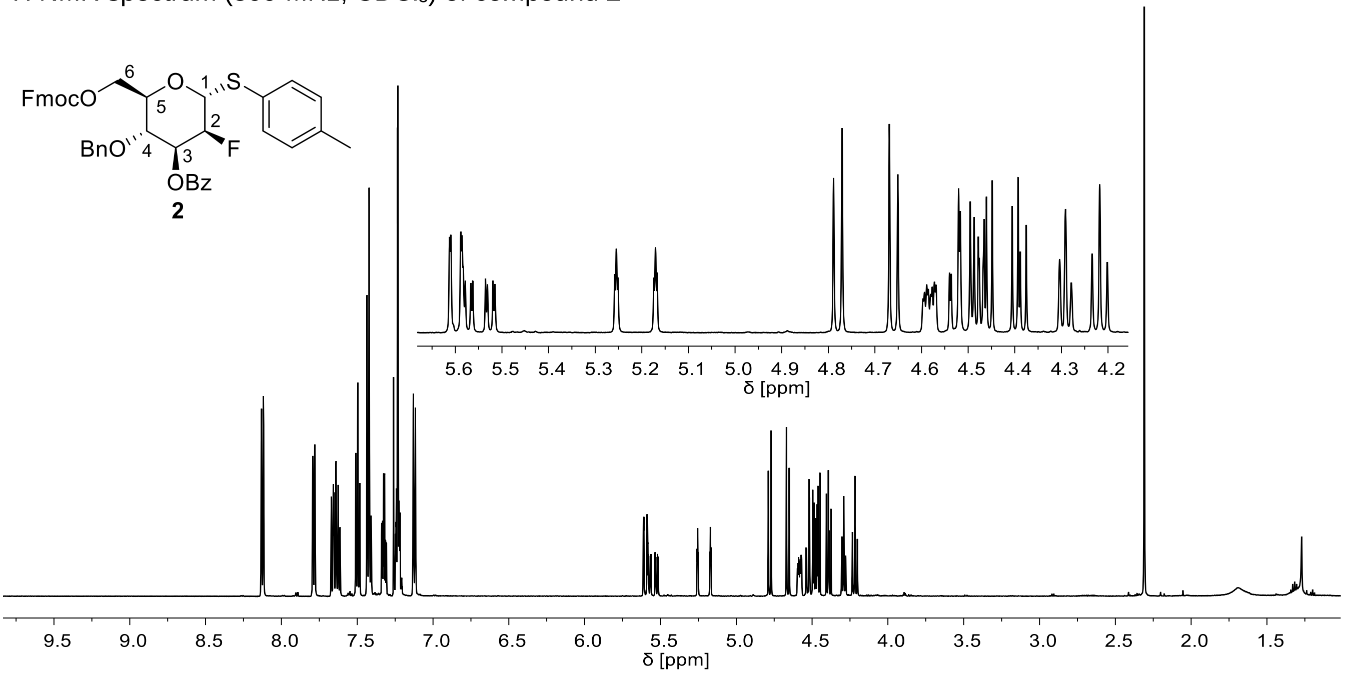

^{13}C NMR spectrum (151 MHz, $CDCl_3$) of compound **2**

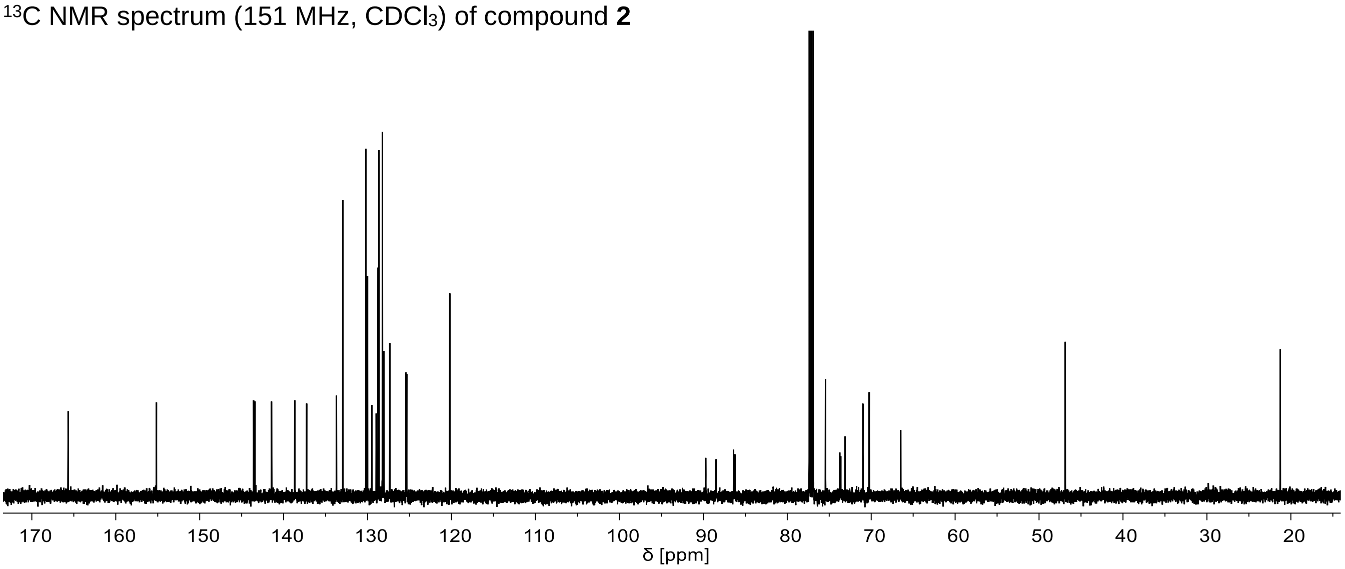

^{19}F NMR spectrum (564 MHz, $CDCl_3$) of compound **2**

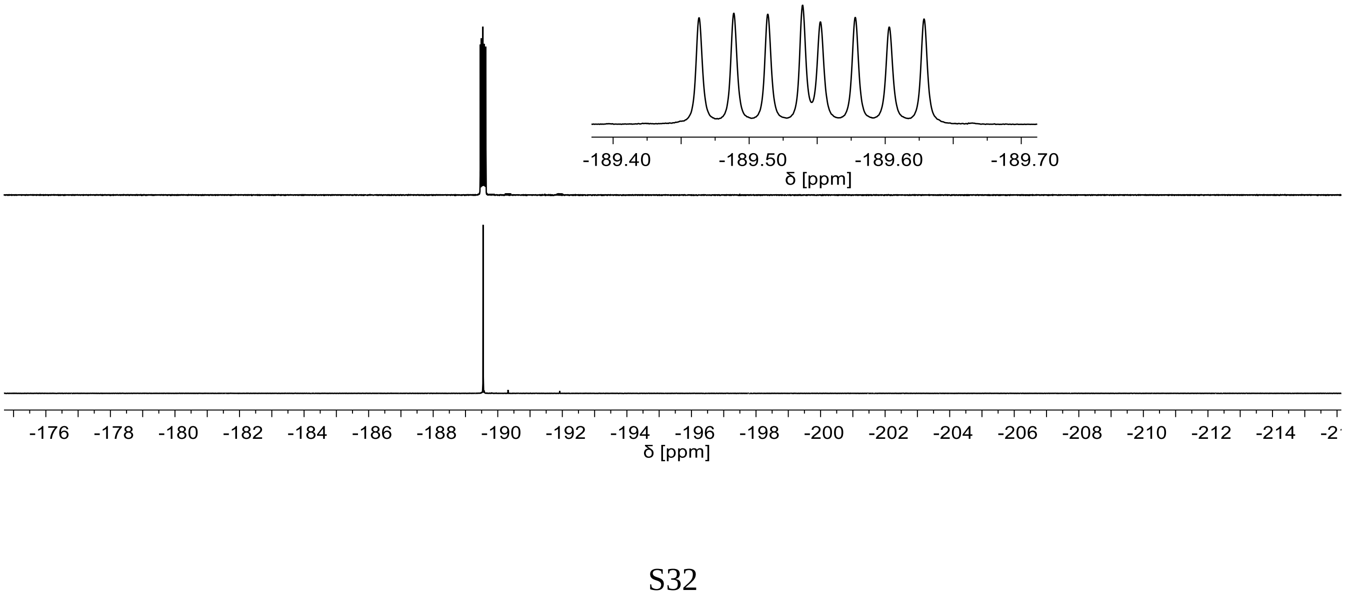

^1H NMR spectrum (599 MHz, $CDCl_3$) of compound **S9**

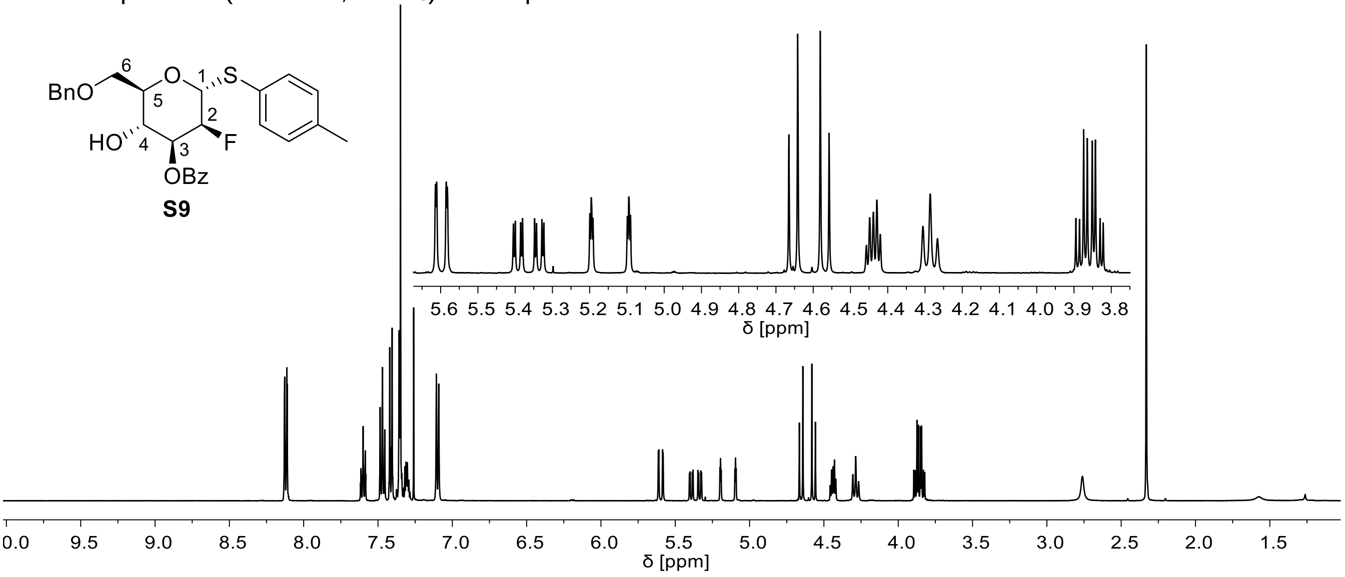

^{13}C NMR spectrum (151 MHz, $CDCl_3$) of compound **S9**

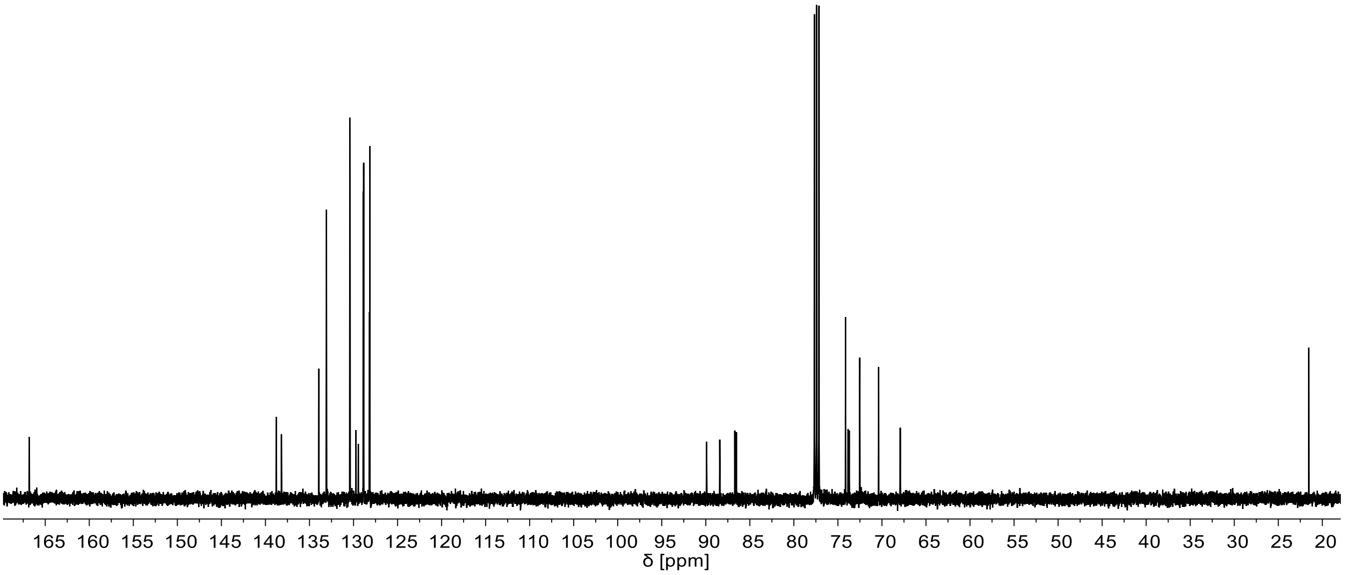

^{19}F NMR spectrum (564 MHz, $CDCl_3$) of compound **S9**

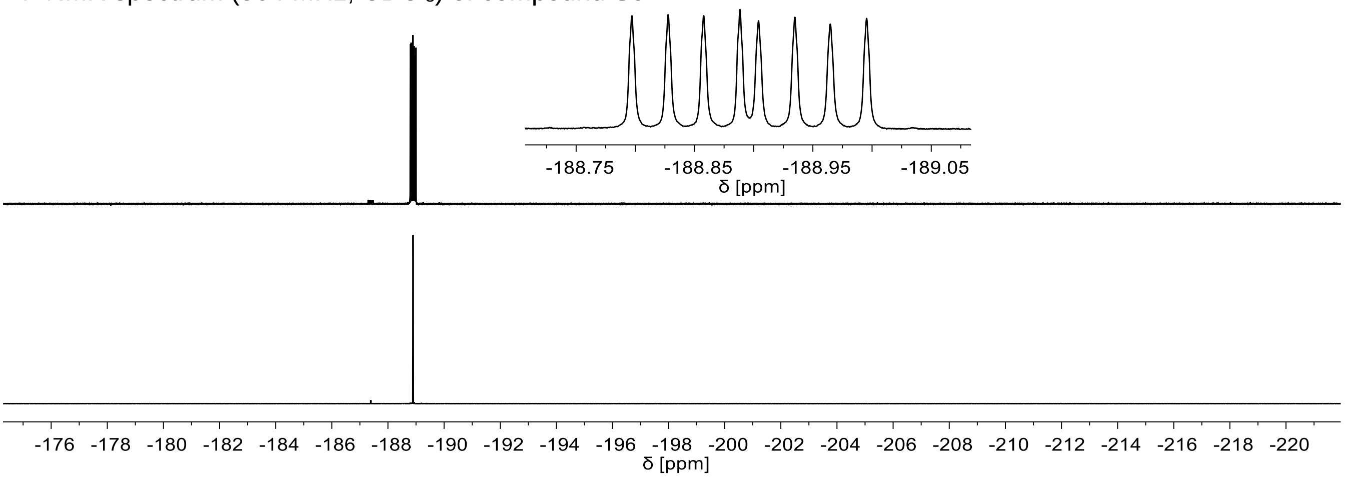

^{1}H NMR spectrum (599 MHz, $CDCl_3$) of compound **8**

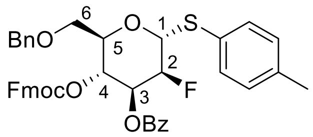

8

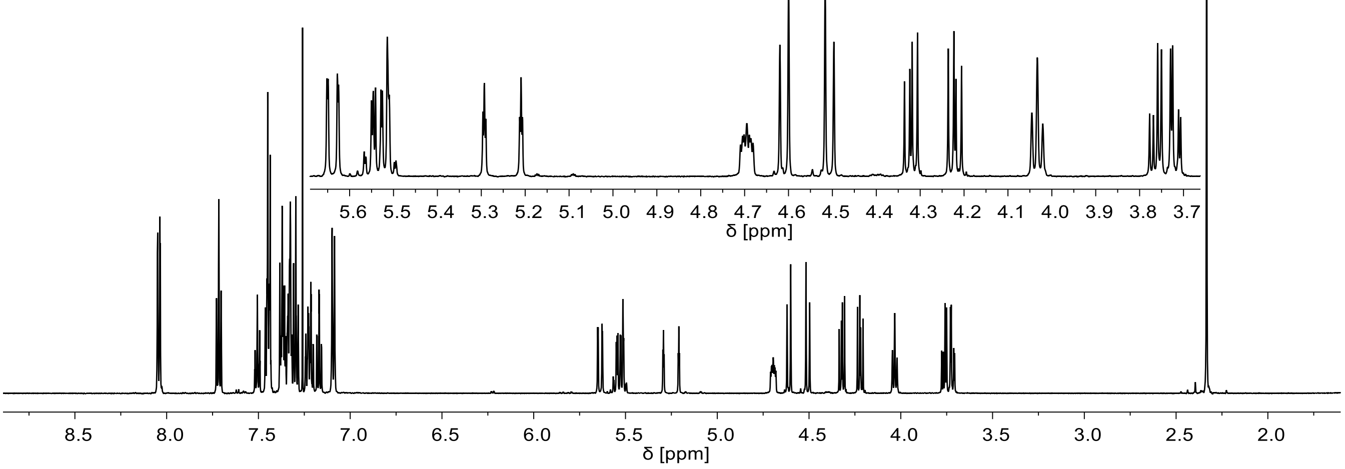

^{13}C NMR spectrum (599 MHz, $CDCl_3$) of compound **8**

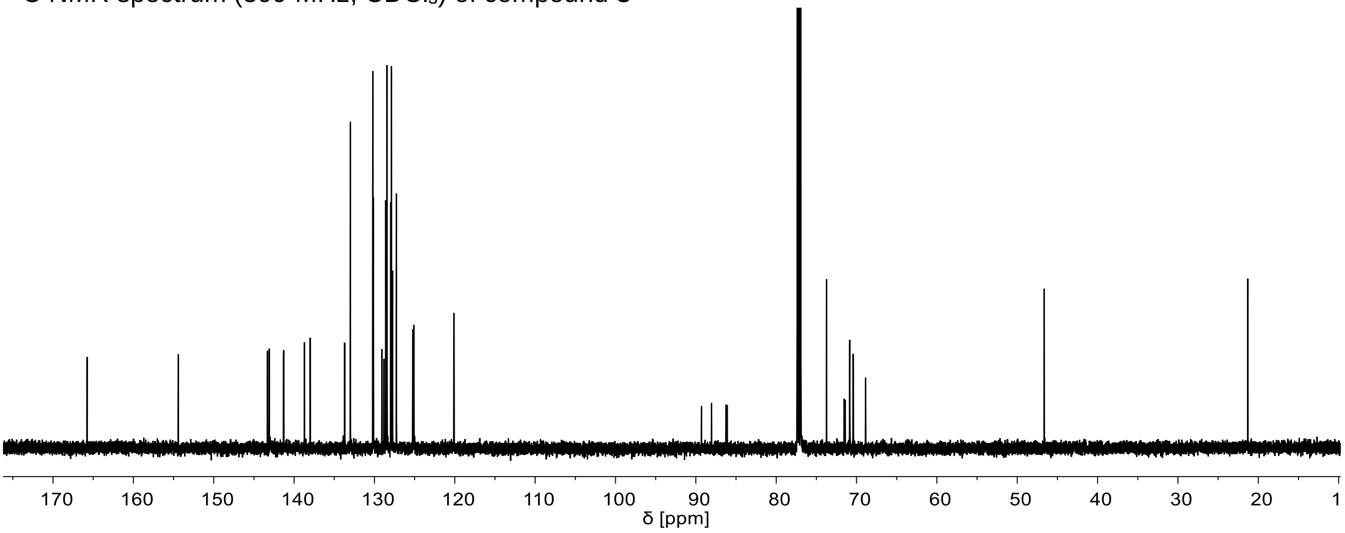

^{19}F NMR spectrum (564 MHz, $CDCl_3$) of compound **8**

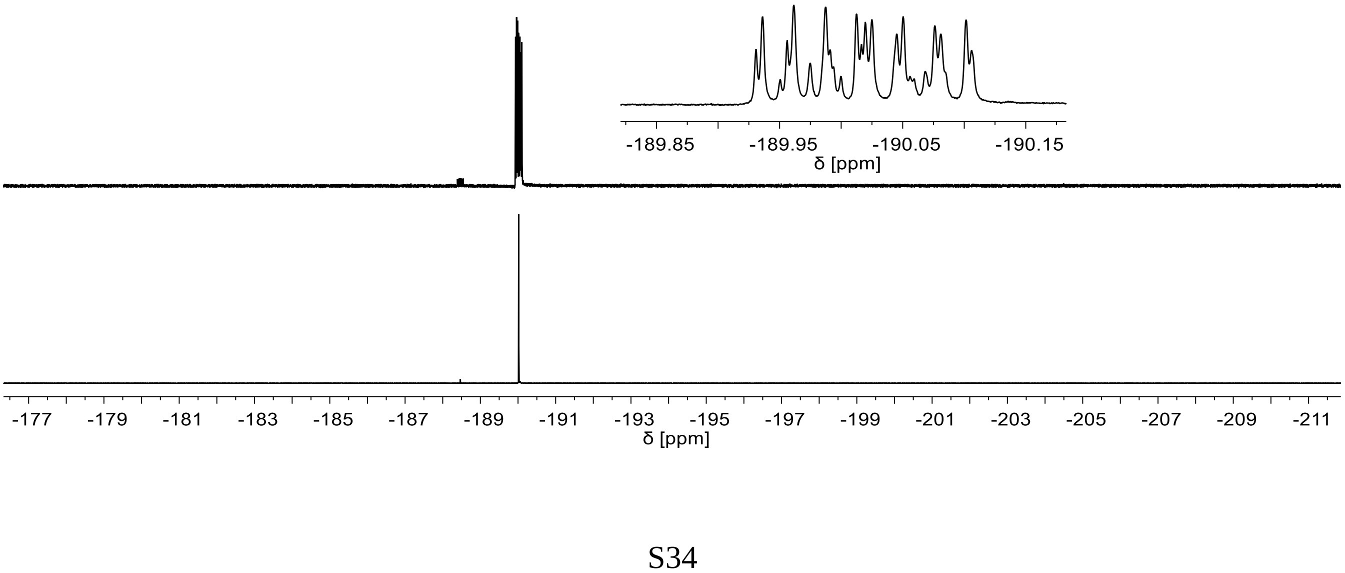

SUPPORTING INFORMATION

^{1}H NMR spectrum (599 MHz, CD_2Cl_2) of compound **S10**

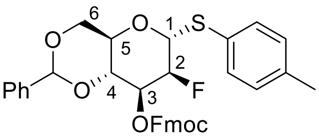

S10

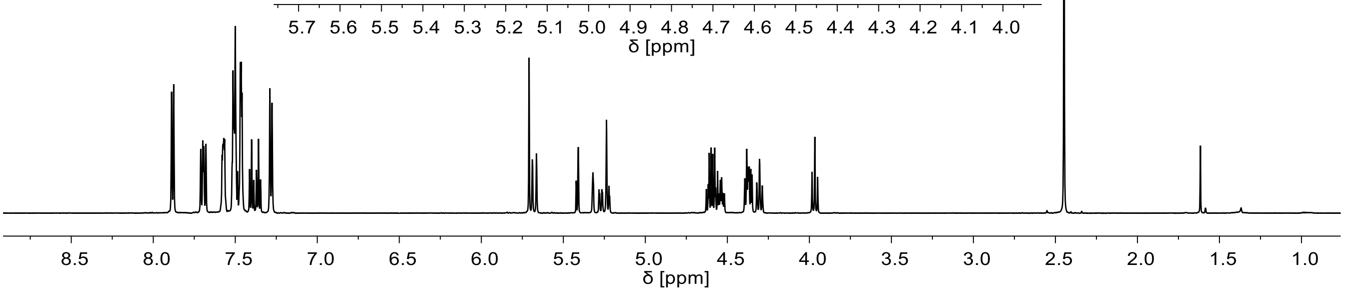

^{13}C NMR spectrum (151 MHz, CD_2Cl_2) of compound **S10**

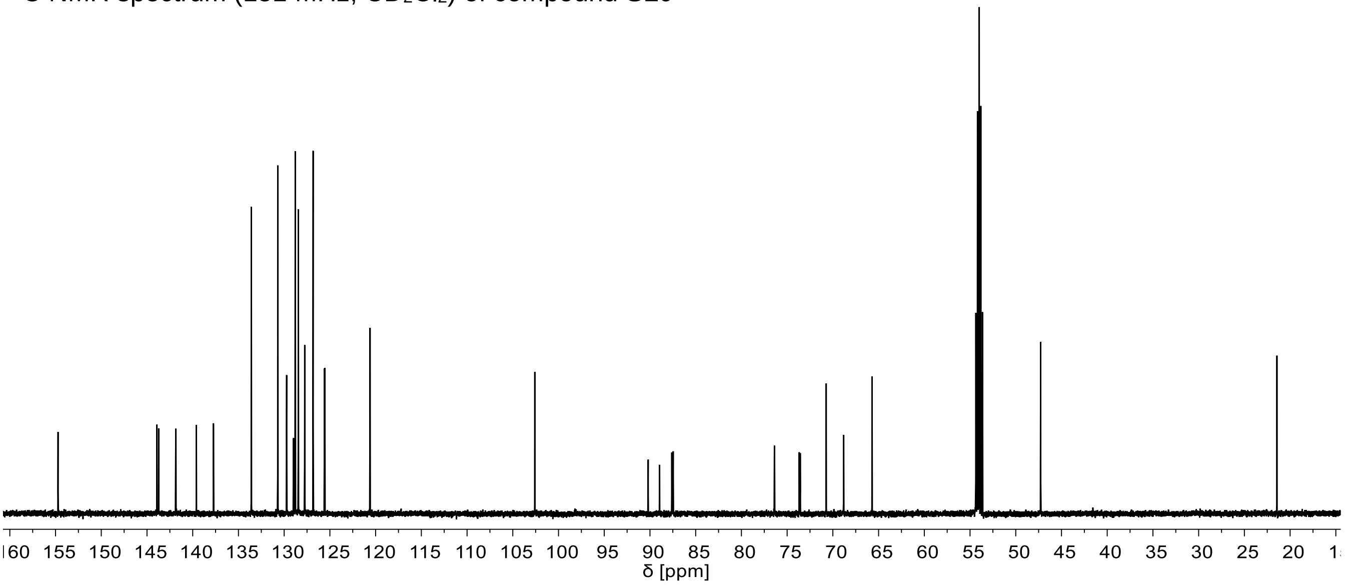

^{19}F NMR spectrum (564 MHz, CD_2Cl_2) of compound **S10**

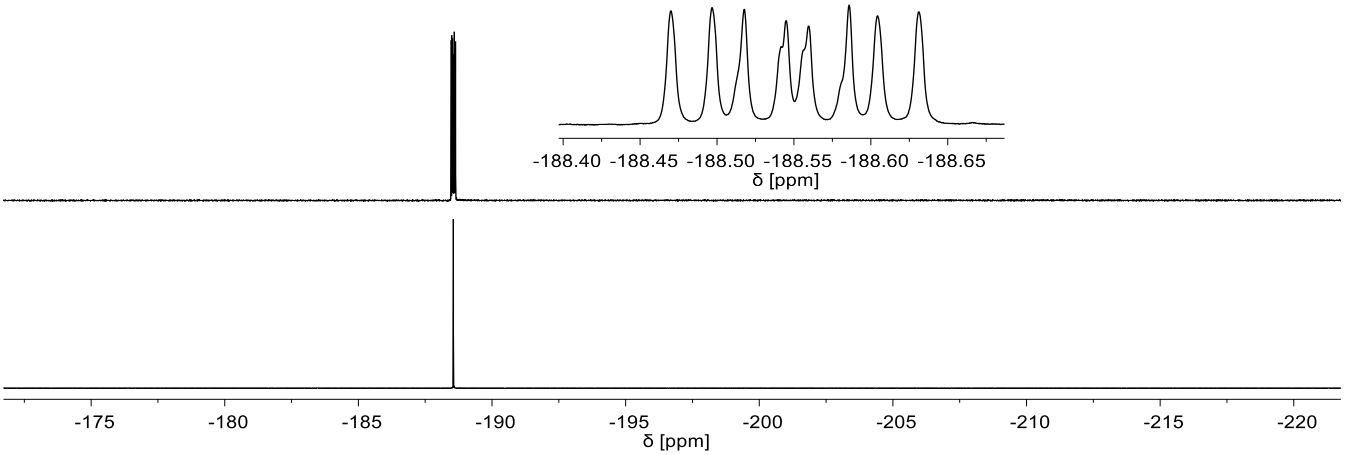

^{1}H NMR spectrum (599 MHz, $CDCl_3$) of compound **S11**

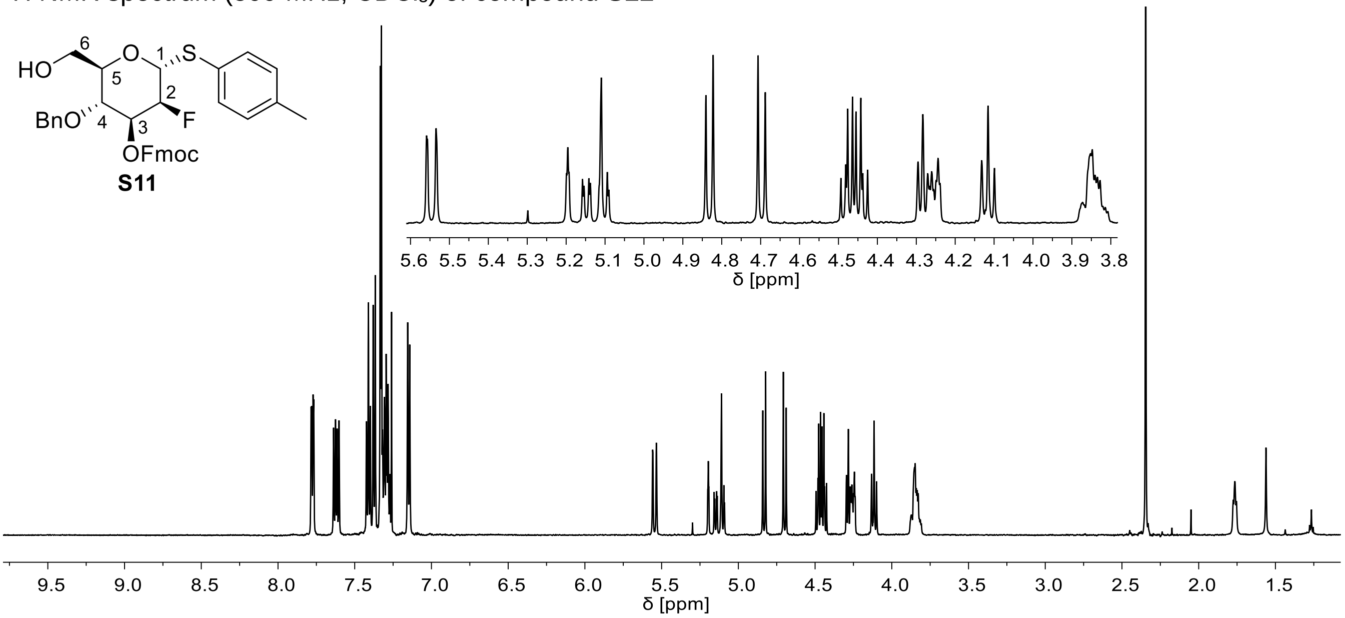

^{13}C NMR spectrum (151 MHz, $CDCl_3$) of compound **S11**

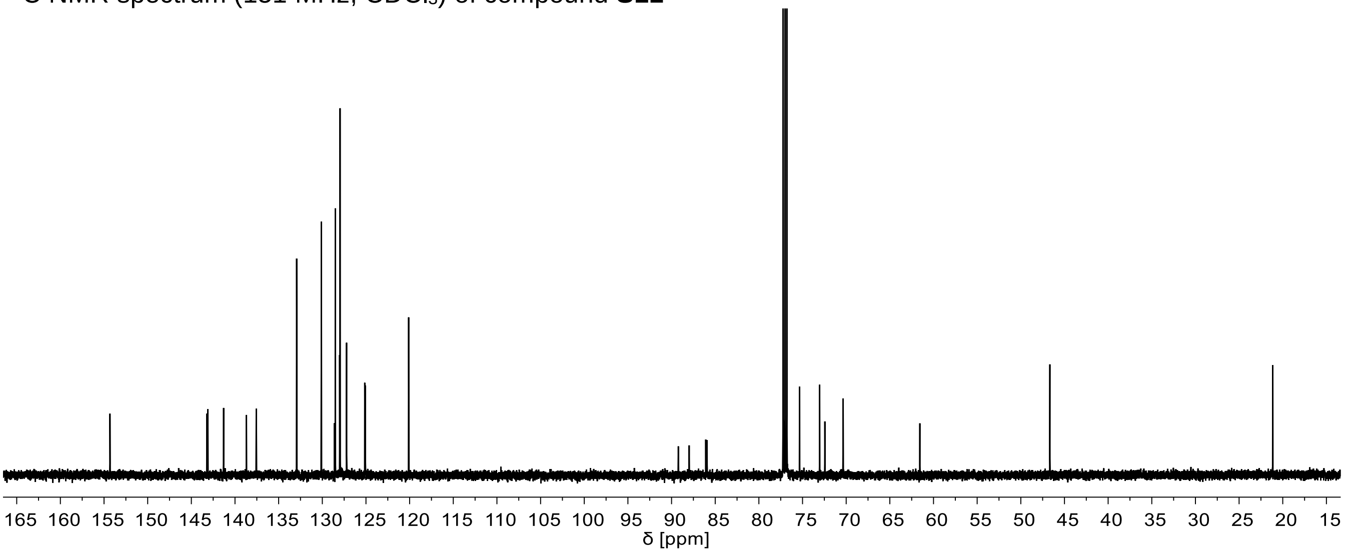

^{19}F NMR spectrum (564 MHz, $CDCl_3$) of compound **S11**

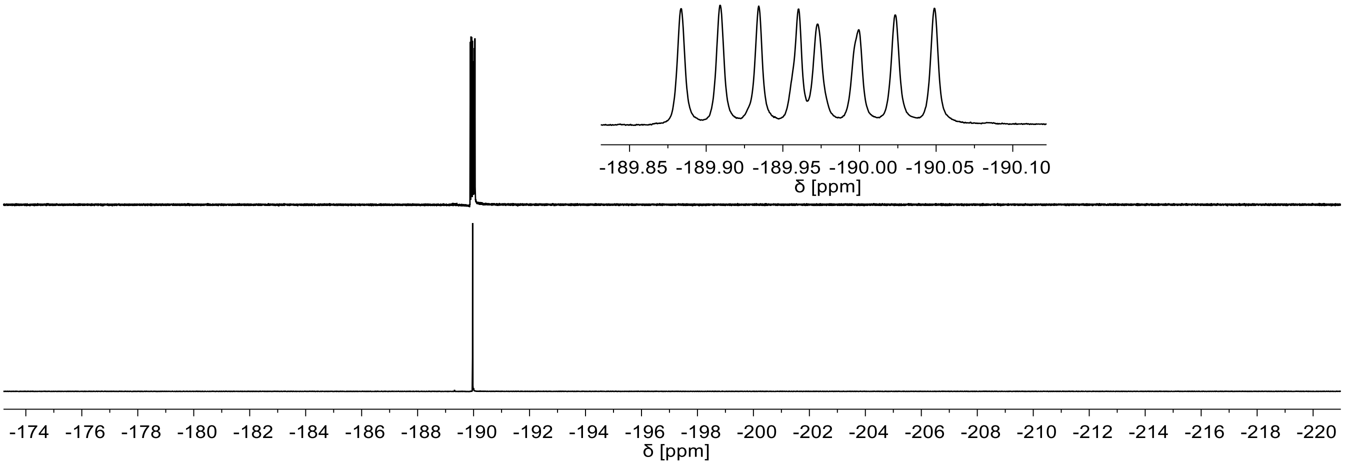

^{1}H NMR spectrum (500 MHz, $CDCl_3$) of compound **9**

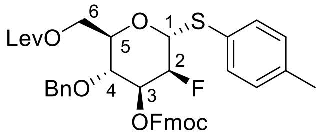

9

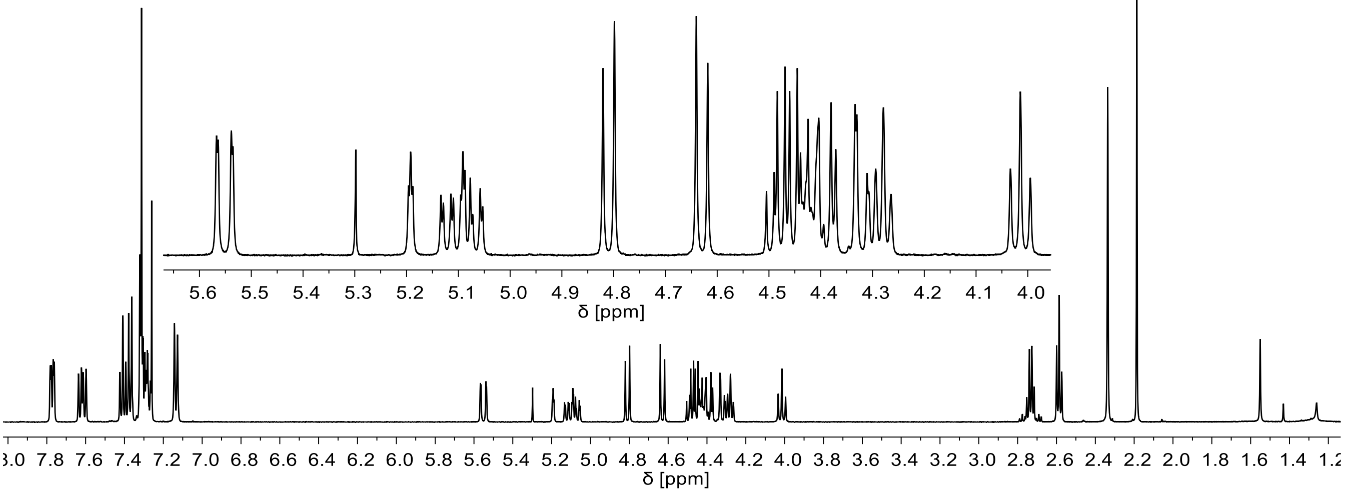

^{13}C NMR spectrum (126 MHz, $CDCl_3$) of compound **9**

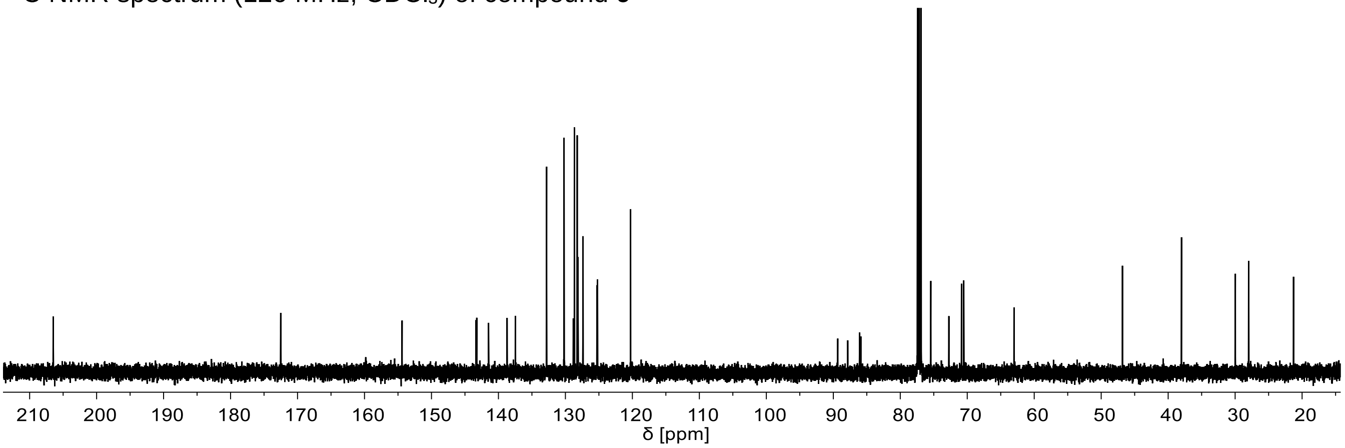

^{19}F NMR spectrum (570 MHz, $CDCl_3$) of compound **9**

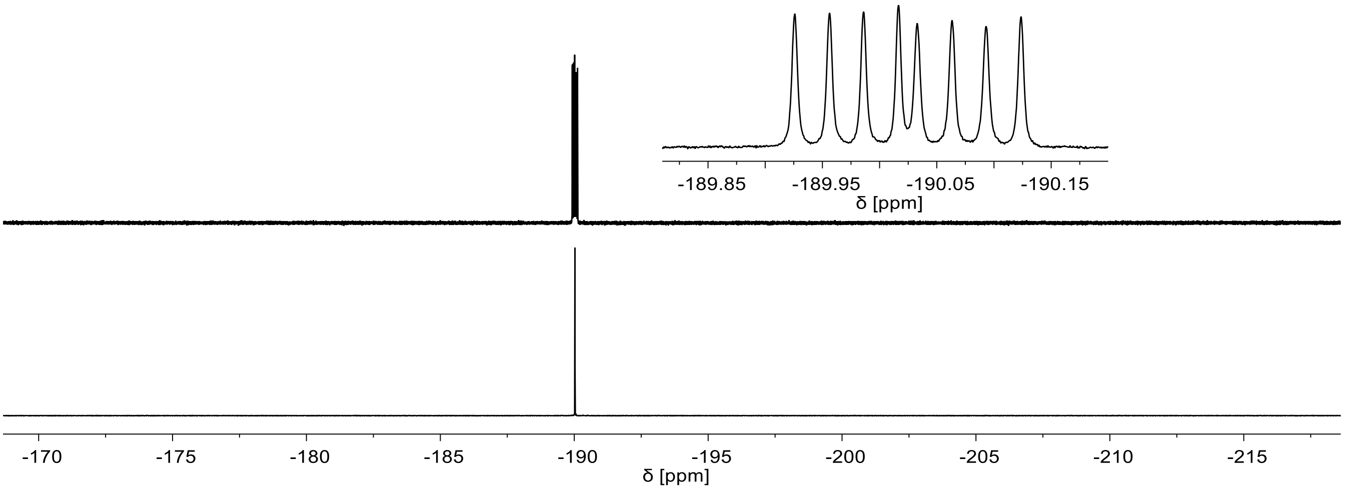

^{1}H NMR spectrum (599 MHz, $CDCl_3$) of compound **4a**

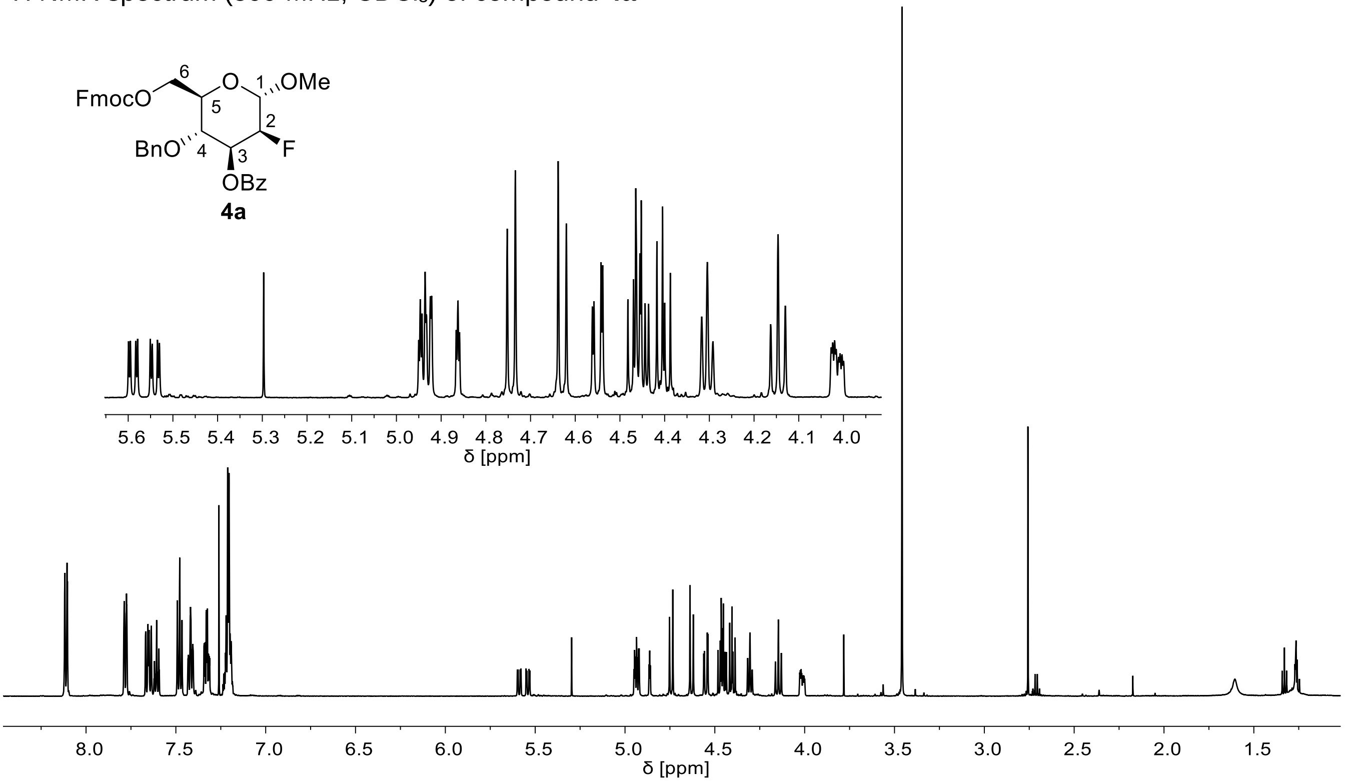

^{13}C NMR spectrum (151 MHz, $CDCl_3$) of compound **4a**

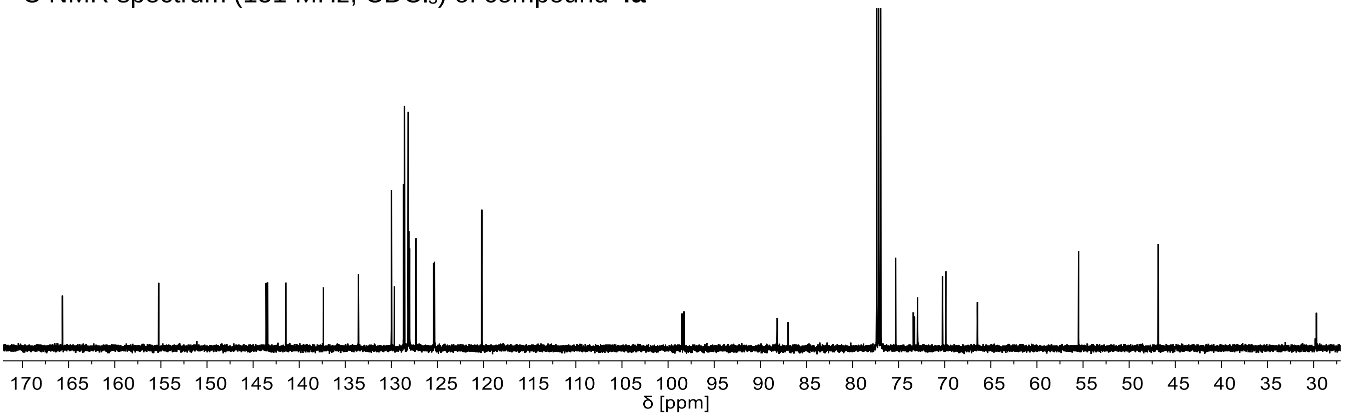

^{19}F NMR spectrum (599 MHz, $CDCl_3$) of compound **4a**

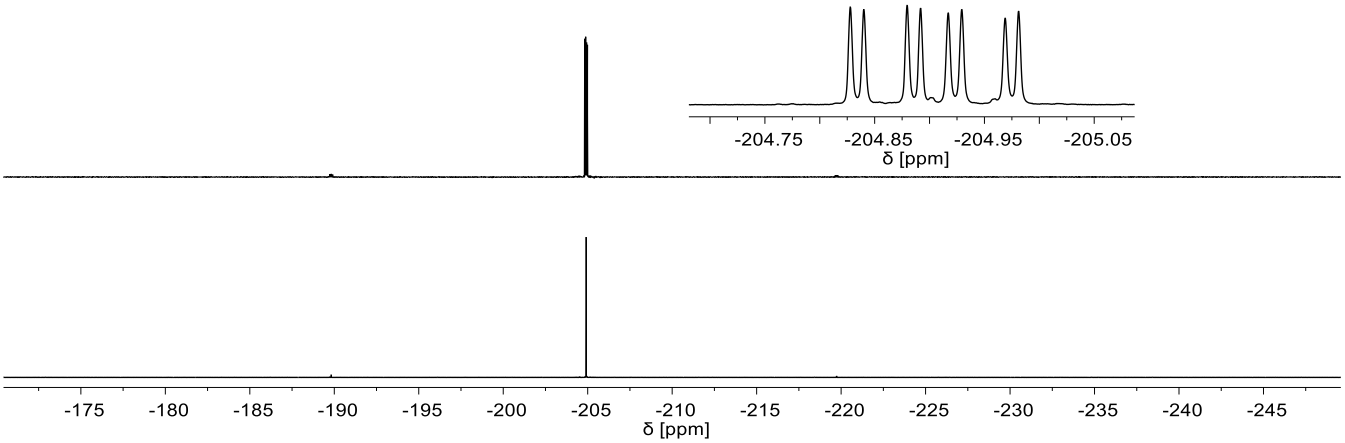

^{1}H NMR spectrum (500 MHz, $CDCl_3$) of compound **7**

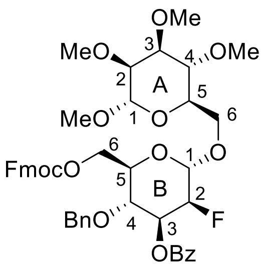

7

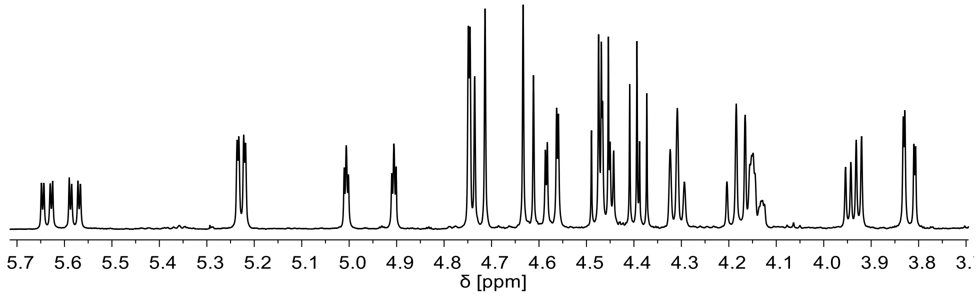

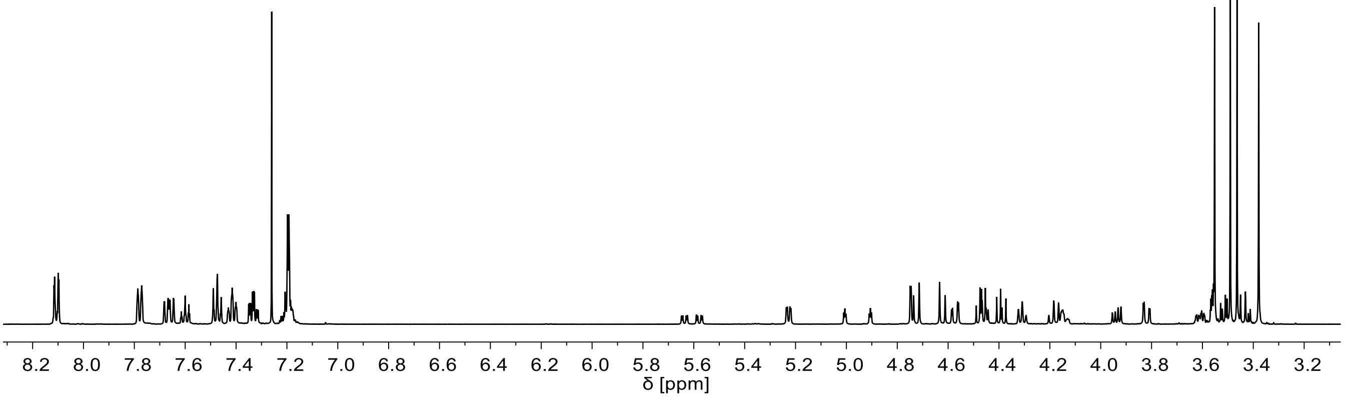

^{13}C NMR spectrum (126 MHz, $CDCl_3$) of compound **7**

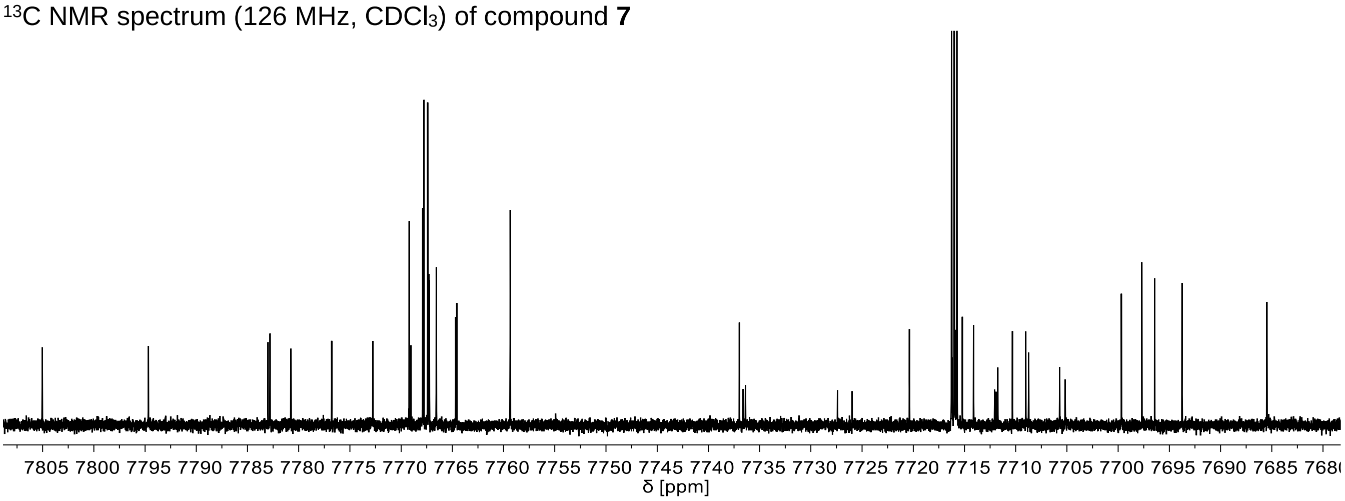

^{19}F NMR spectrum (470 MHz, $CDCl_3$) of compound **7**

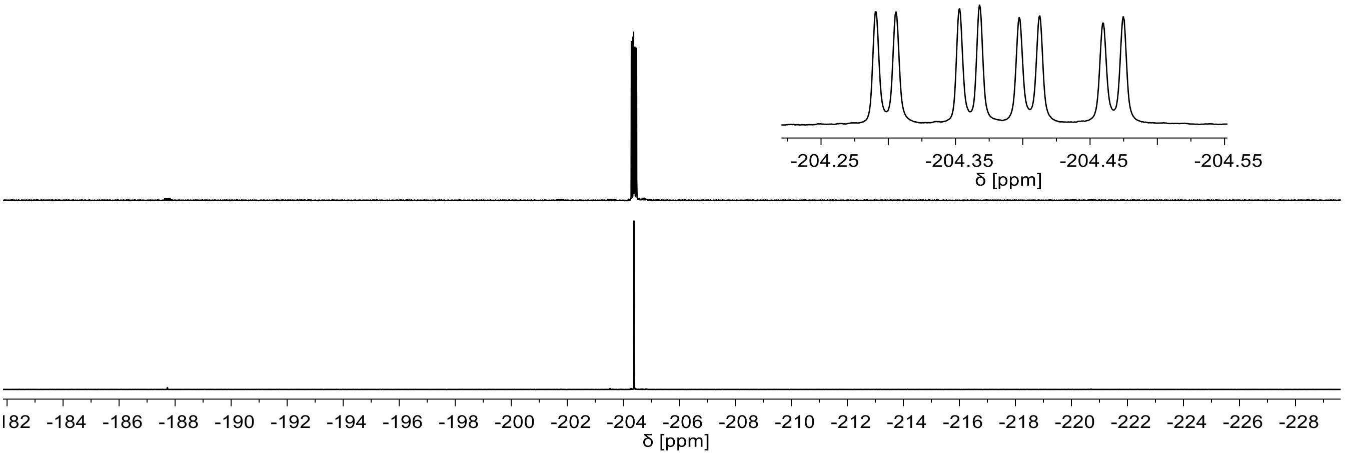

^{1}H NMR spectrum (500 MHz, $CDCl_3$) of compound **10**

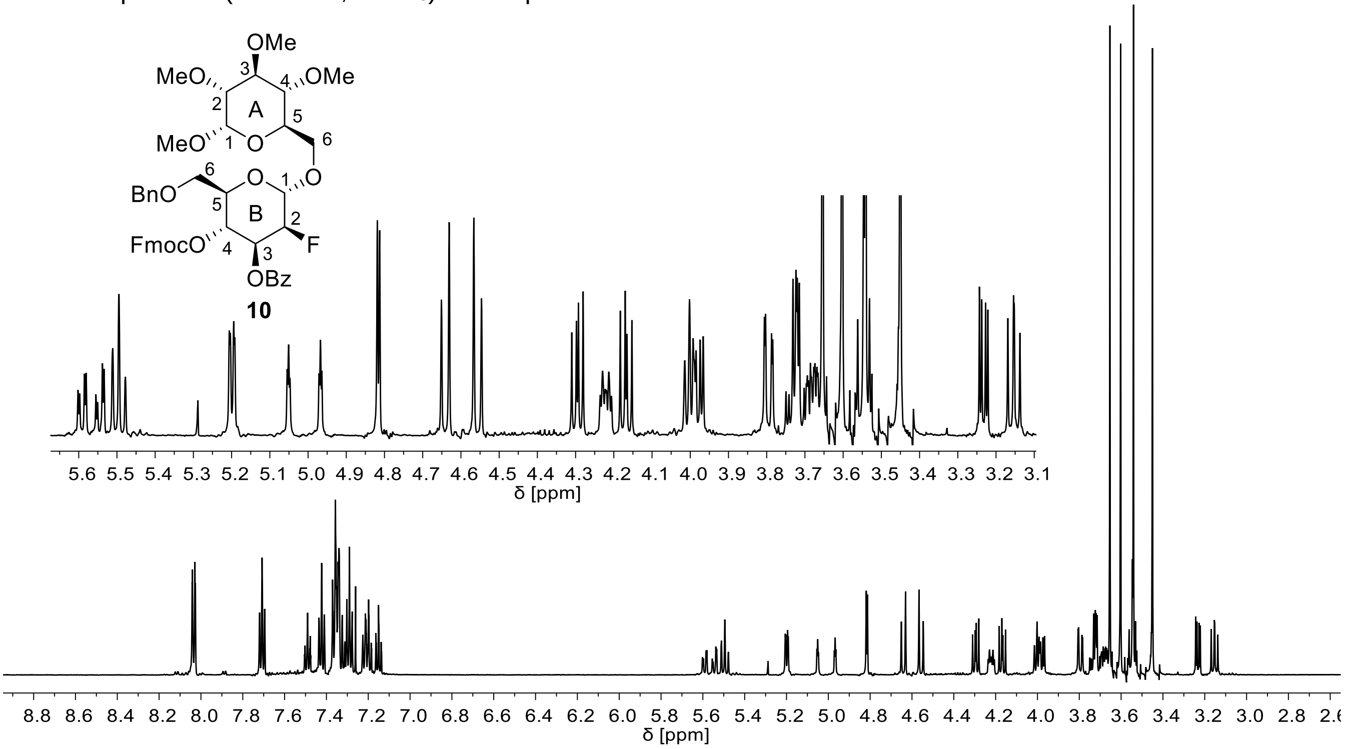

^{13}C NMR spectrum (126 MHz, $CDCl_3$) of compound **10**

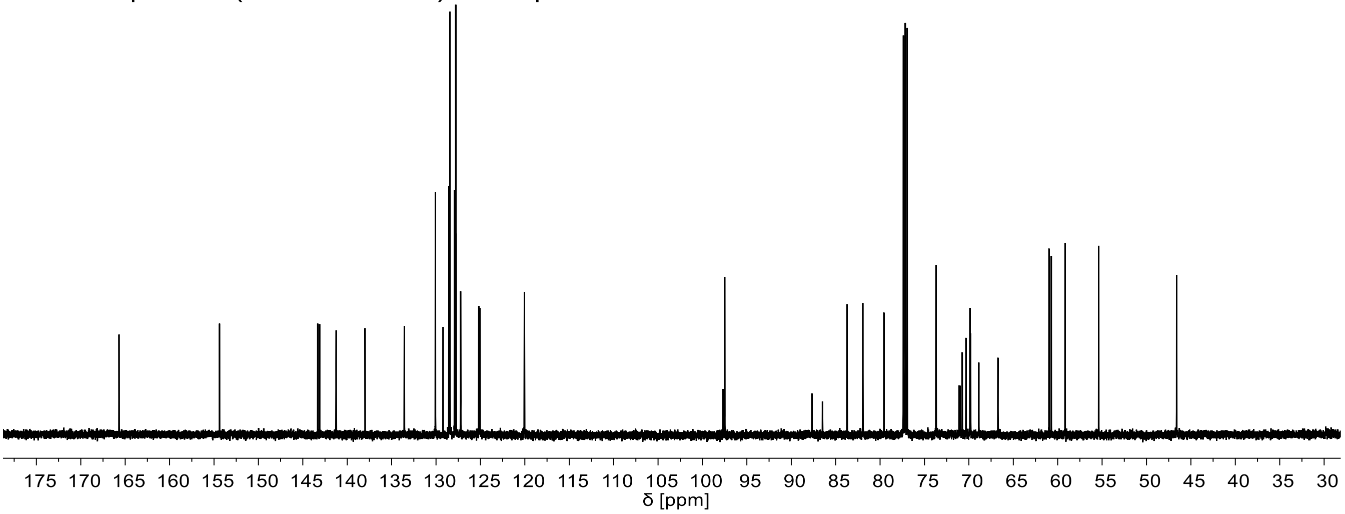

^{19}F NMR spectrum (470 MHz, $CDCl_3$) of compound **10**

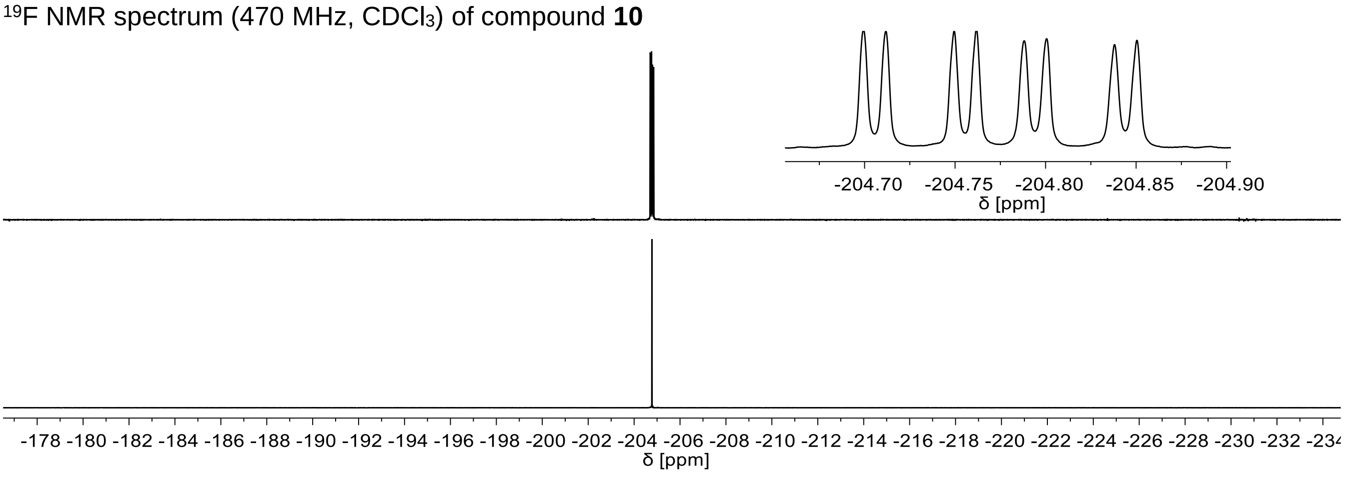

^{1}H NMR spectrum (599 MHz, $CDCl_3$) of compound **11**

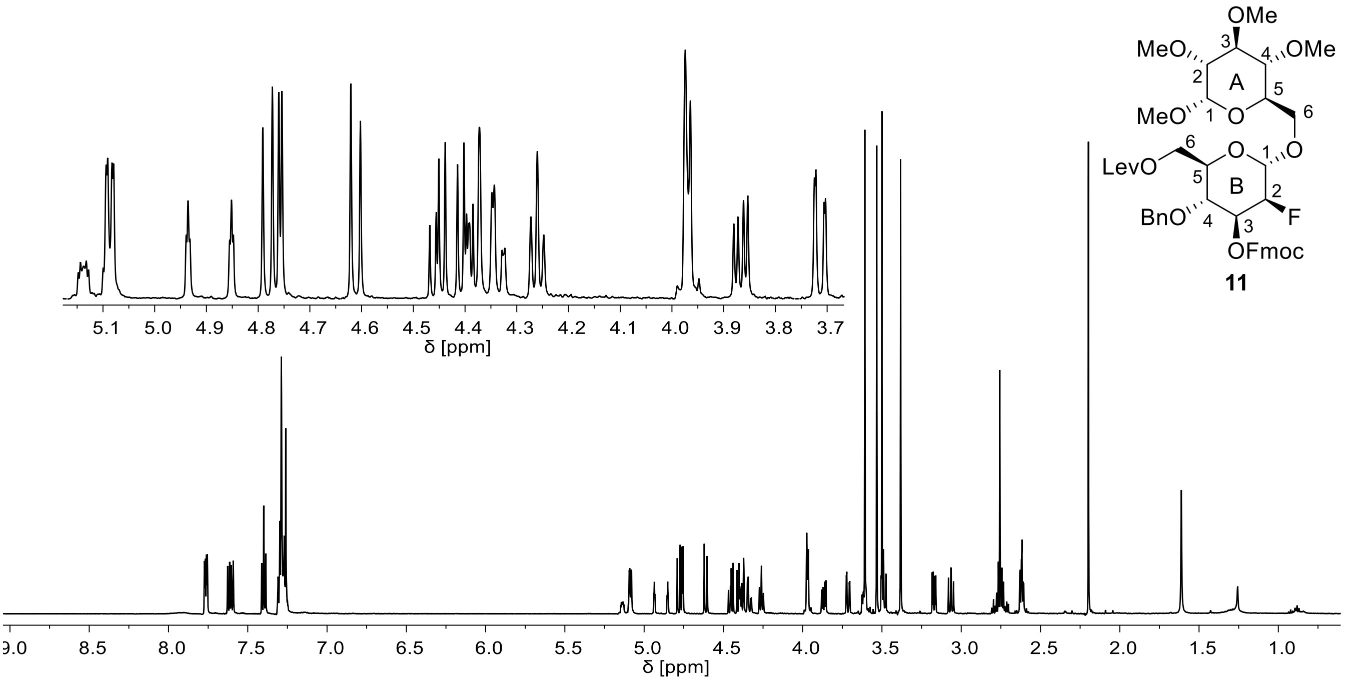

^{13}C NMR spectrum (151 MHz, $CDCl_3$) of compound **11**

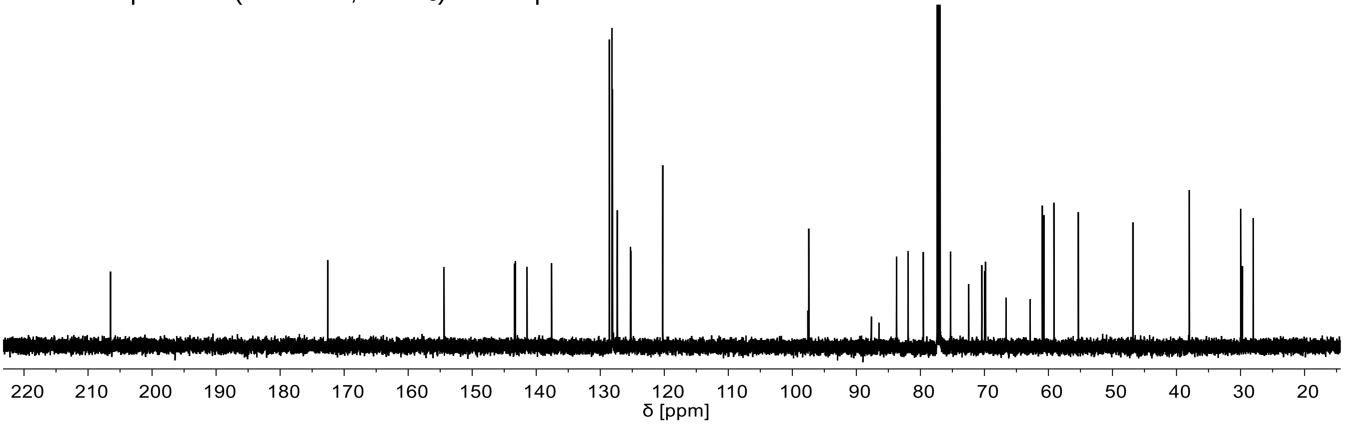

^{19}F NMR spectrum (564 MHz, $CDCl_3$) of compound **11**

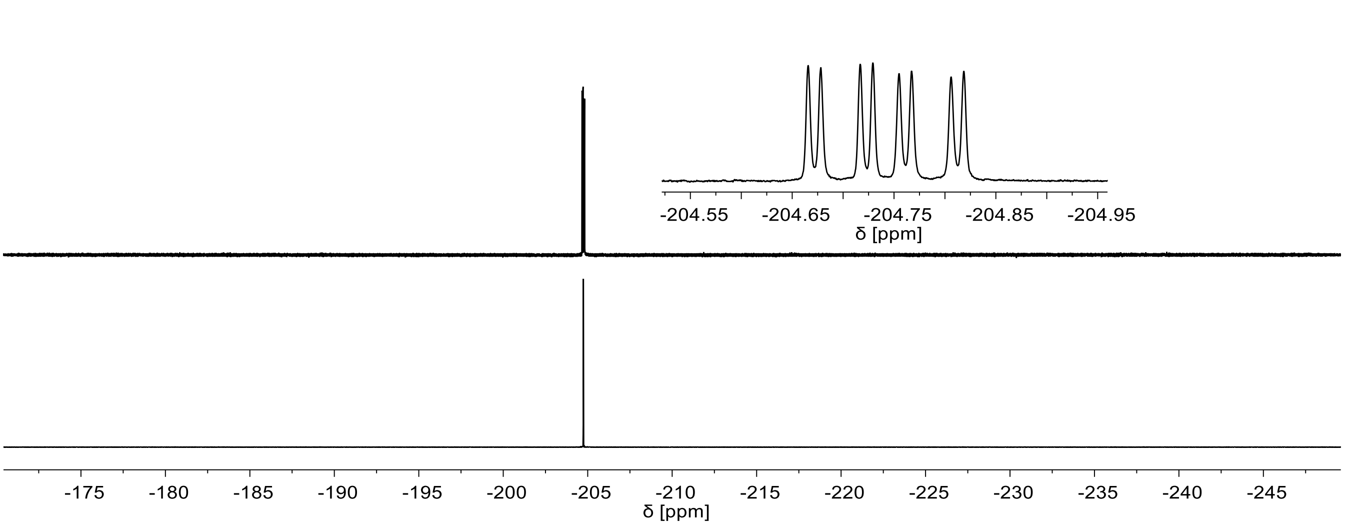

^{1}H NMR spectrum (500 MHz, $CDCl_3$) of compound **S12**

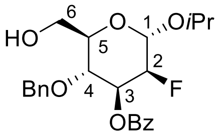

S12

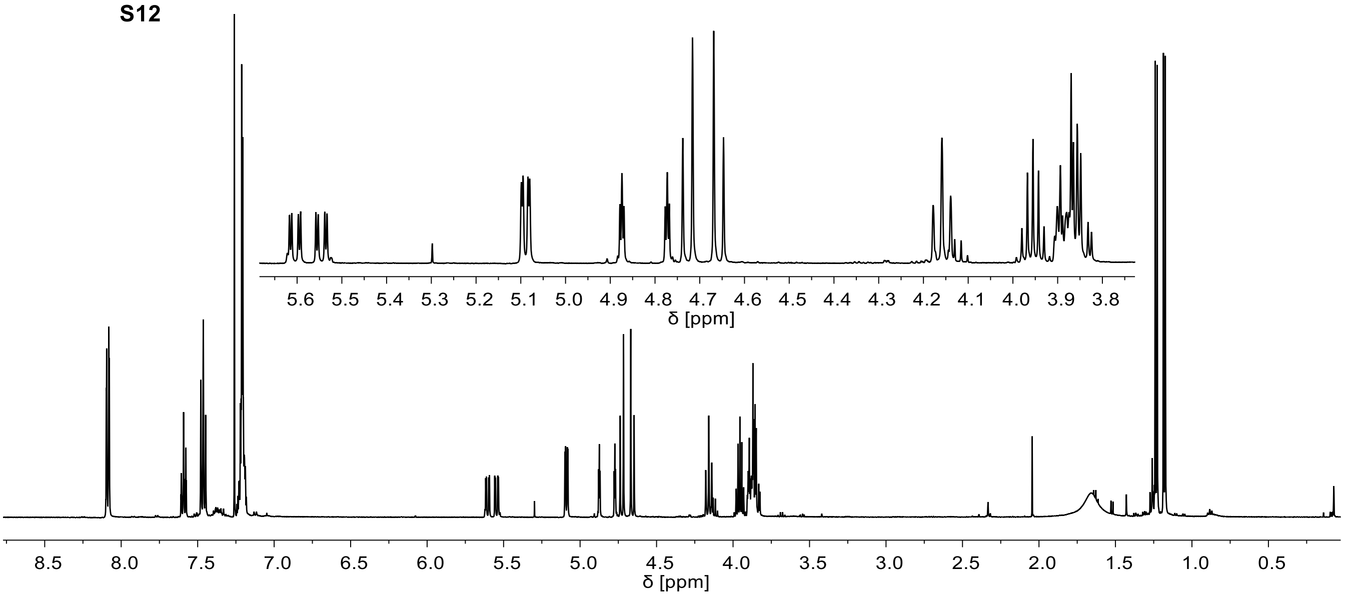

^{13}C NMR spectrum (126 MHz, $CDCl_3$) of compound **S12**

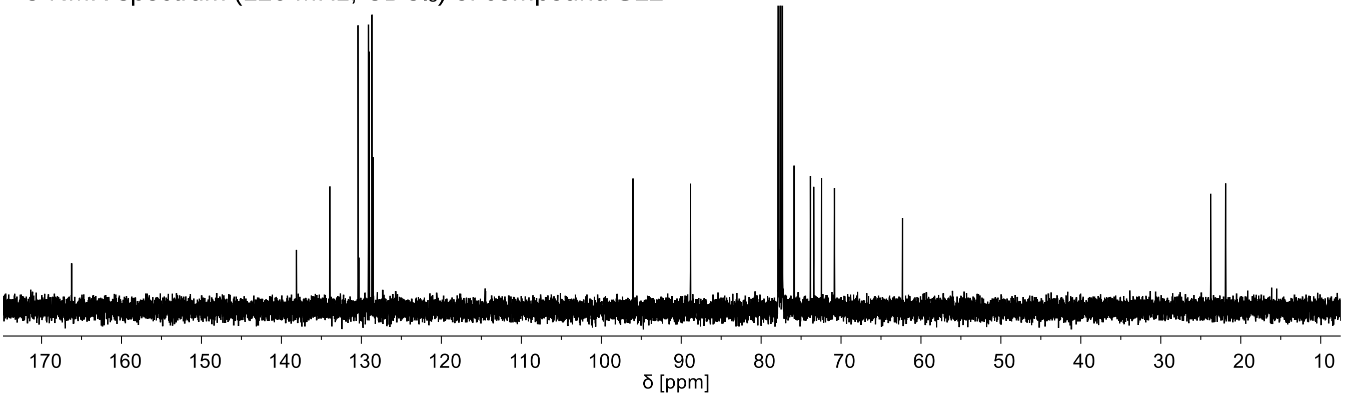

^{19}F NMR spectrum (470 MHz, $CDCl_3$) of compound **S12**

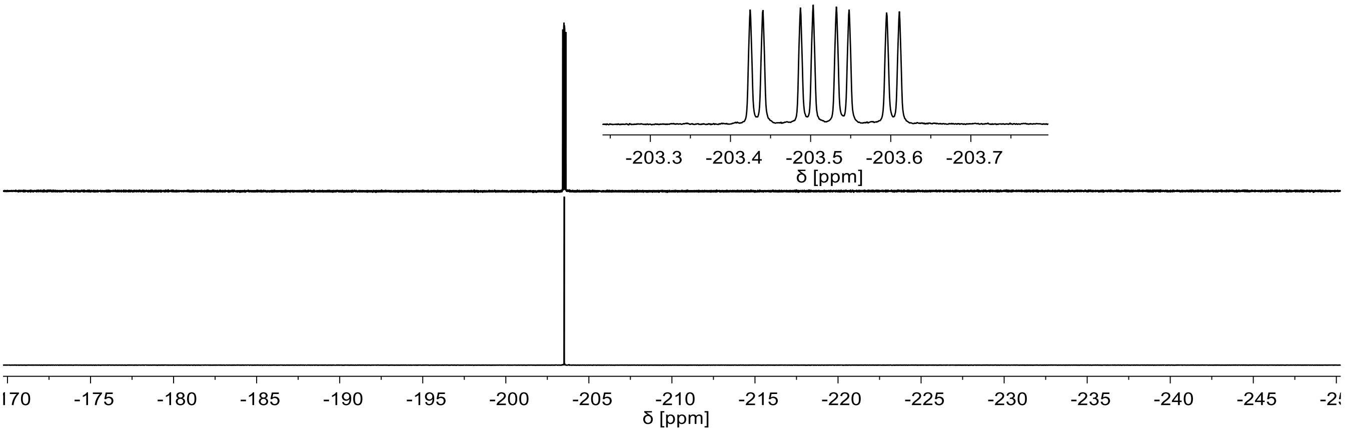

^{1}H NMR spectrum (599 MHz, $CDCl_3$) of compound **S13**

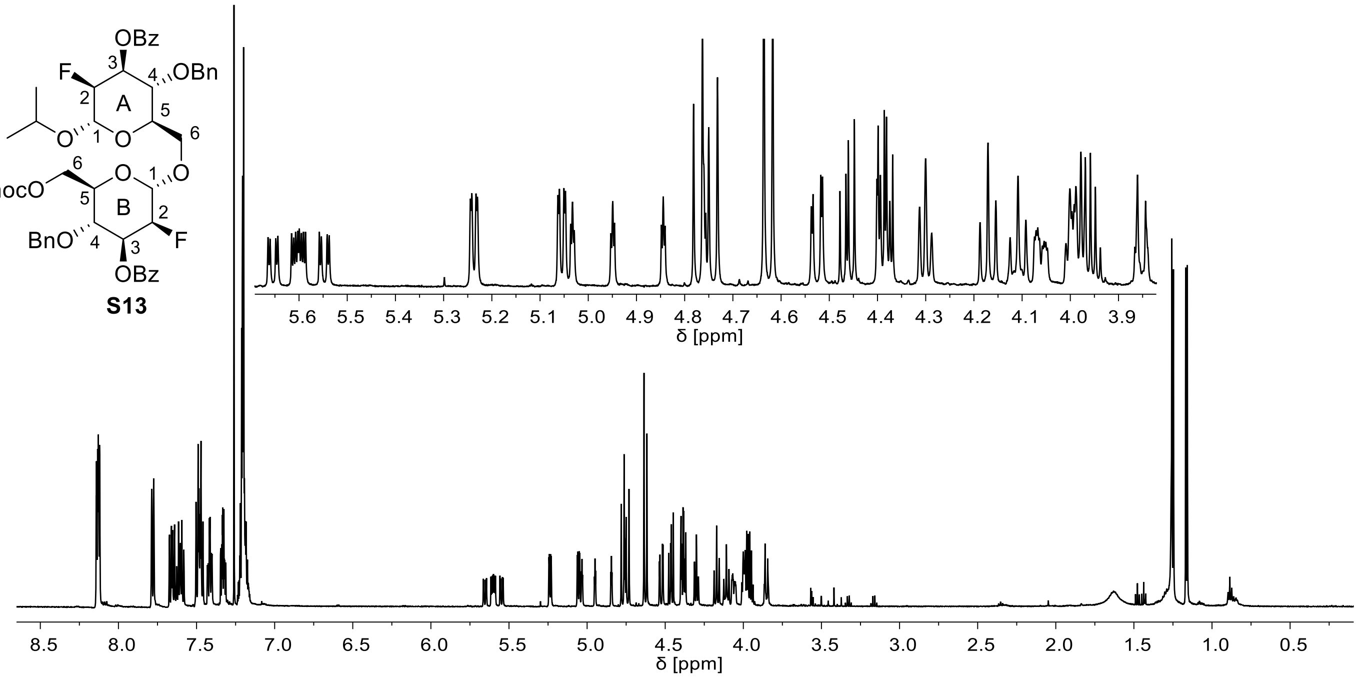

^{13}C NMR spectrum (151 MHz, $CDCl_3$) of compound **S13**

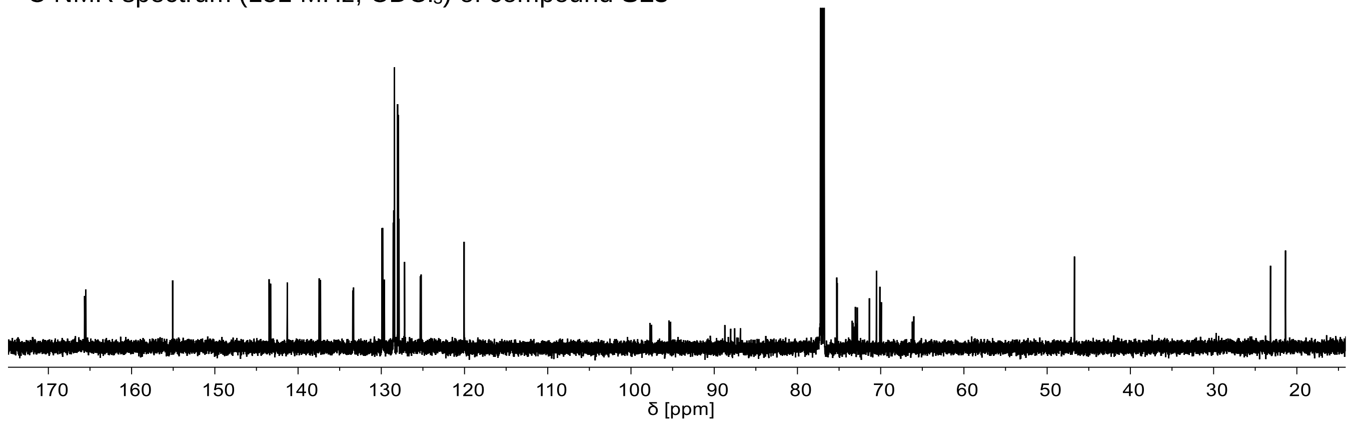

^{19}F NMR spectrum (564 MHz, $CDCl_3$) of compound **S13**

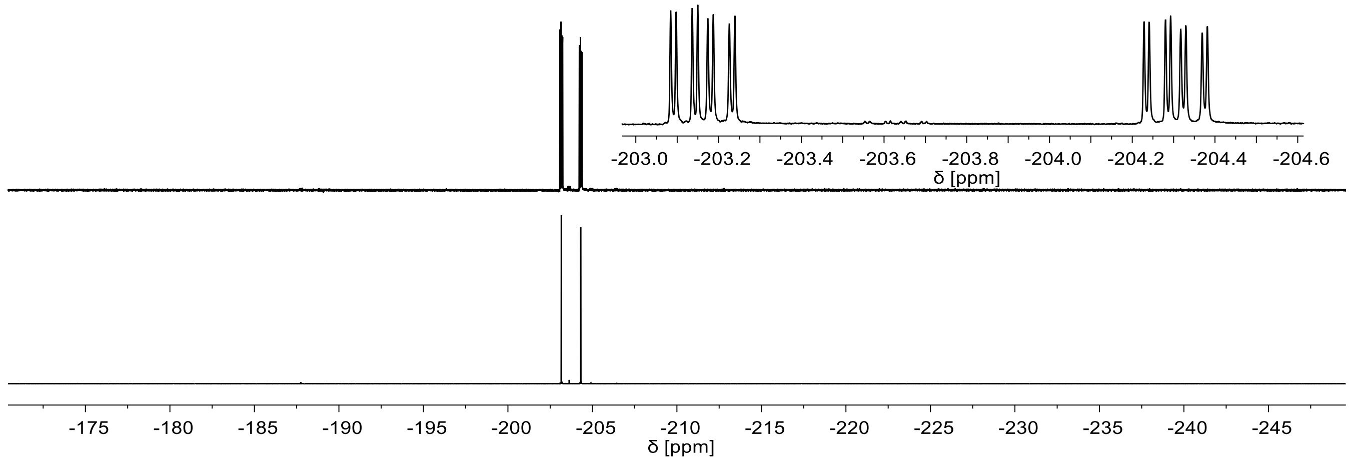

^1H NMR spectrum (599 MHz, $CDCl_3$) of compound **13**

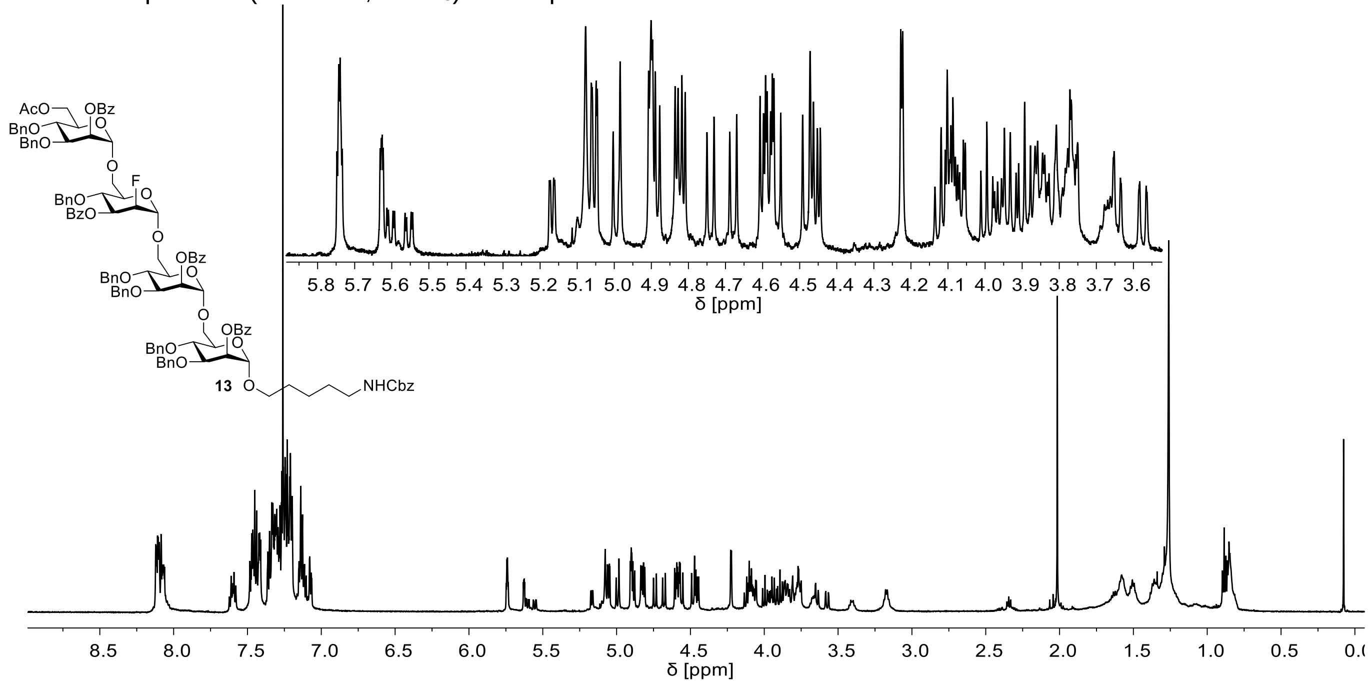

^{13}C NMR spectrum (151 MHz, $CDCl_3$) of compound **13**

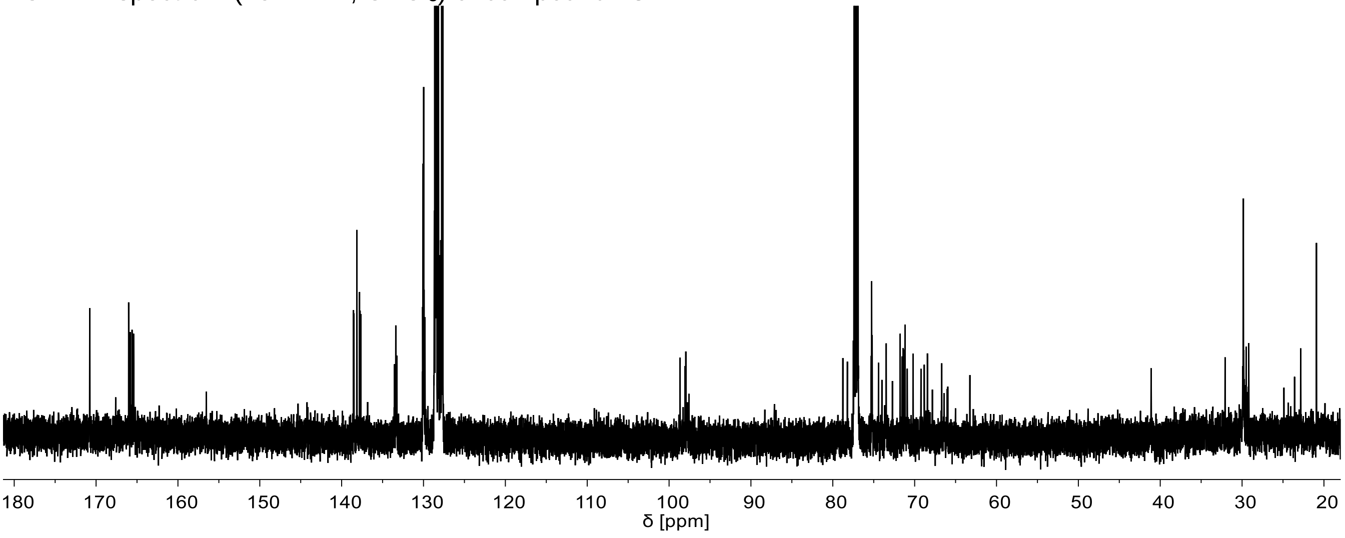

^{19}F NMR spectrum (564 MHz, $CDCl_3$) of compound **13**

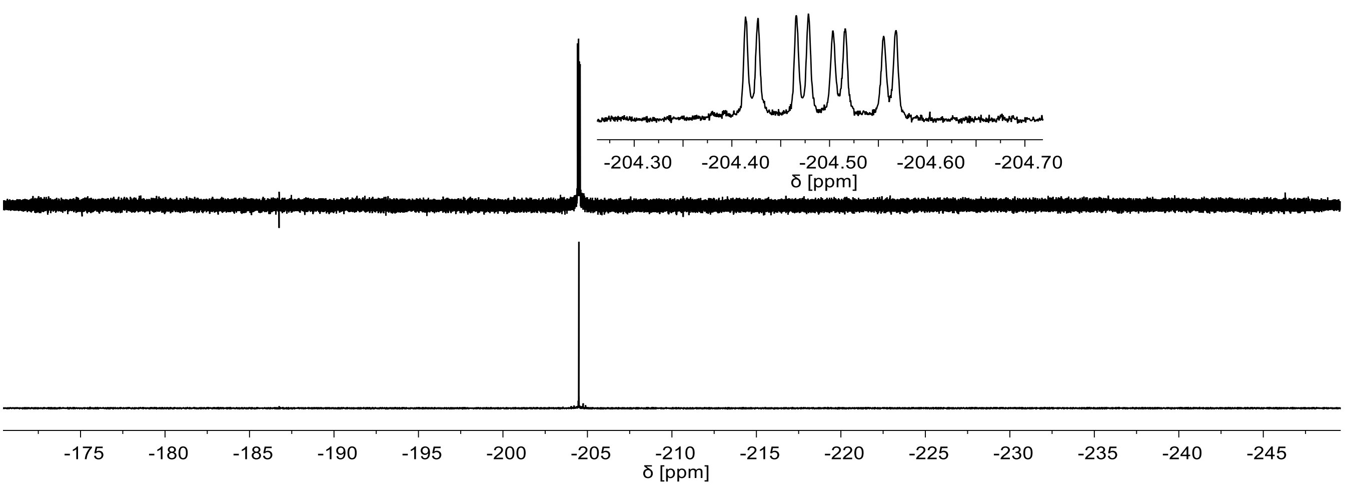

^{1}H NMR spectrum (599 MHz, $CDCl_3$) of compound **17**

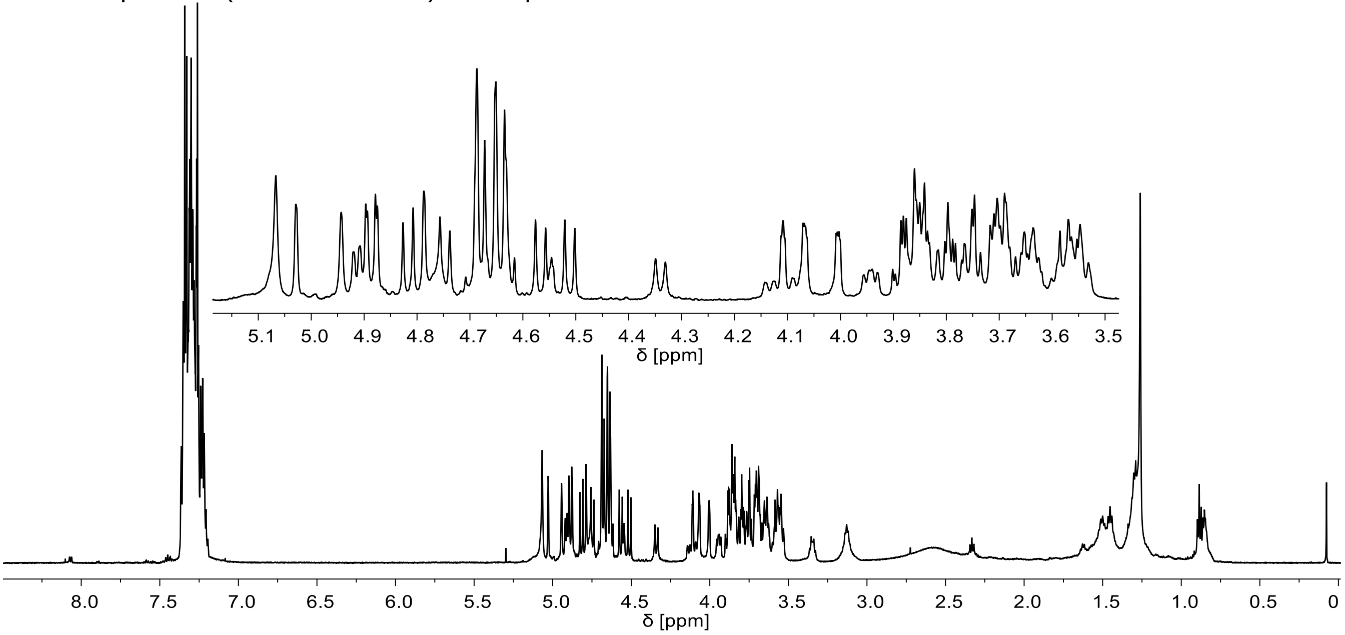

^{13}C NMR spectrum (151 MHz, $CDCl_3$) of compound **17**

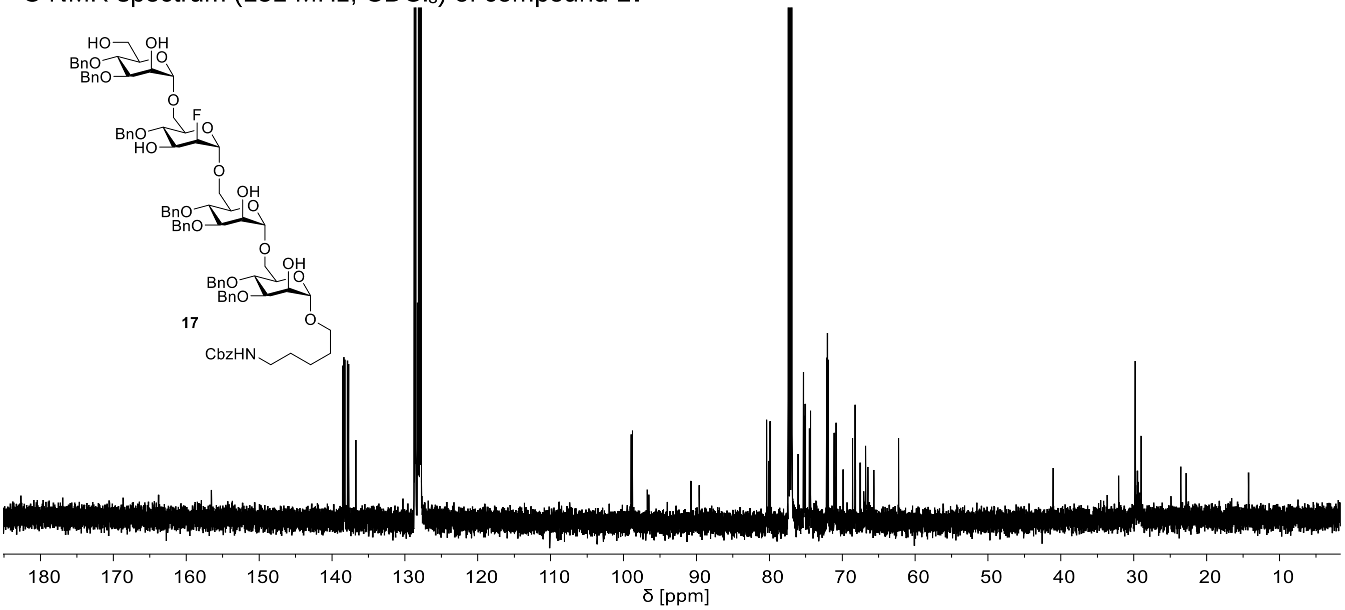

^{19}F NMR spectrum (564 MHz, $CDCl_3$) of compound **17**

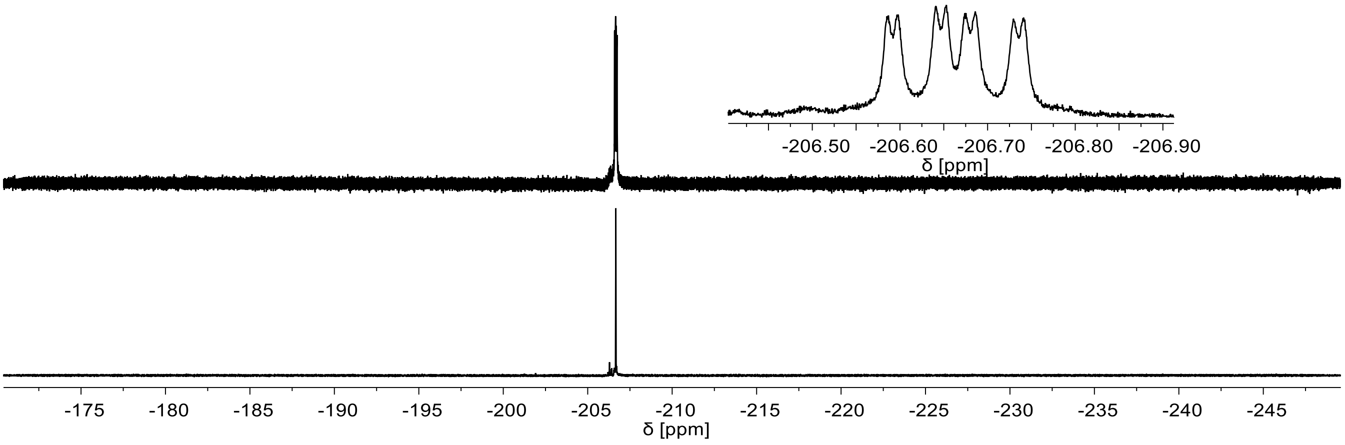

^{1}H NMR spectrum (599 MHz, D_2O) of compound **18**

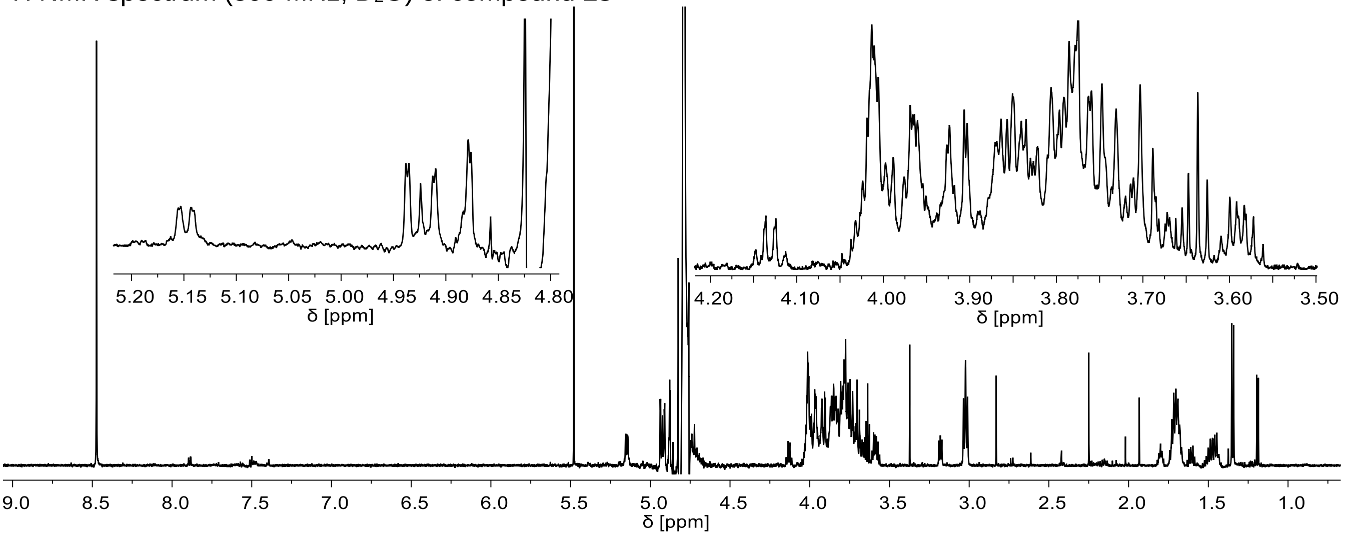

^{13}C NMR spectrum (599 MHz, D_2O) of compound **18**

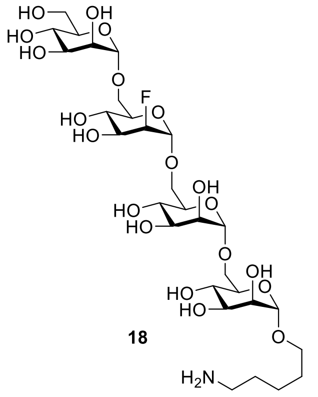

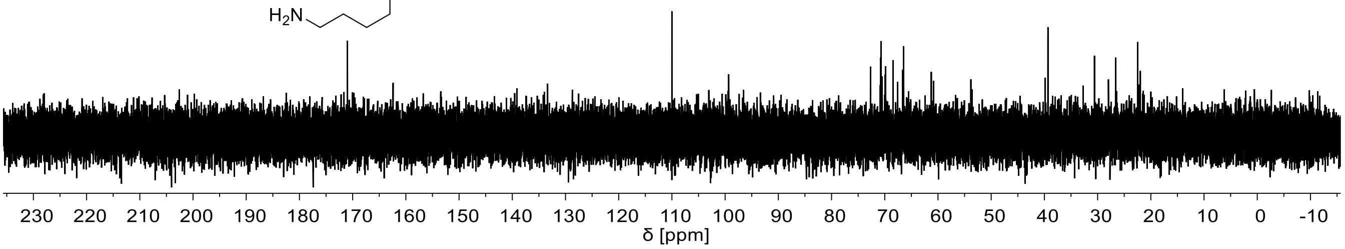

^{19}F NMR spectrum (564 MHz, D_2O) of compound **18**

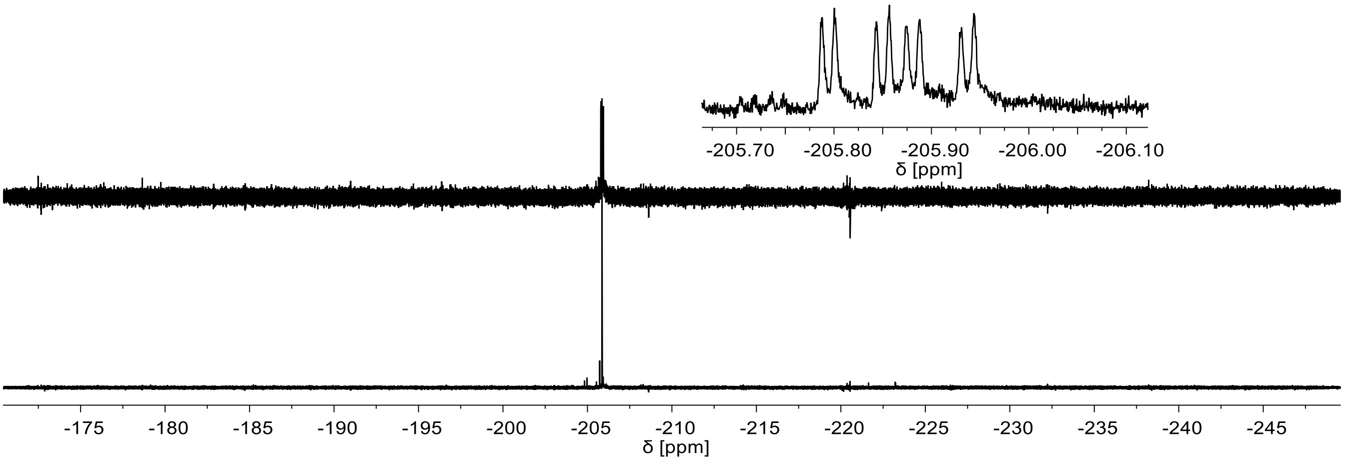

^{1}H NMR spectrum (599 MHz, $CDCl_3$) of compound **15**

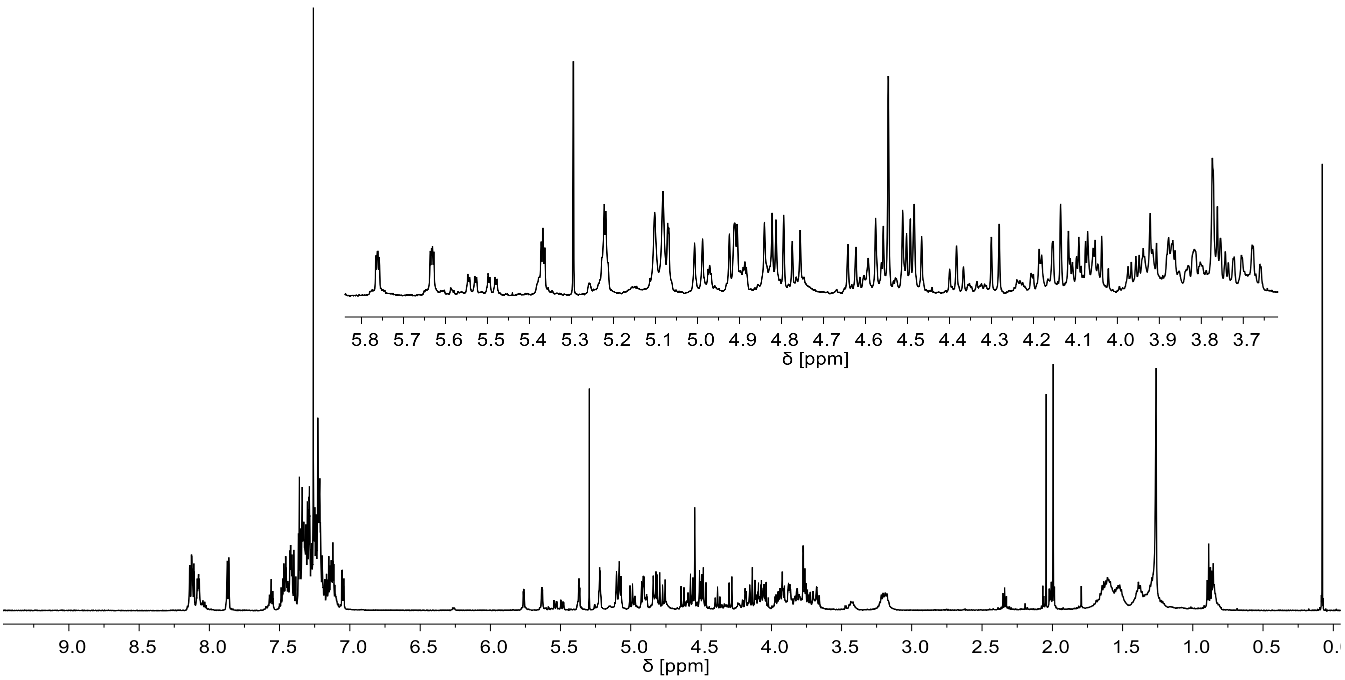

^{13}C NMR spectrum (151 MHz, $CDCl_3$) of compound **15**

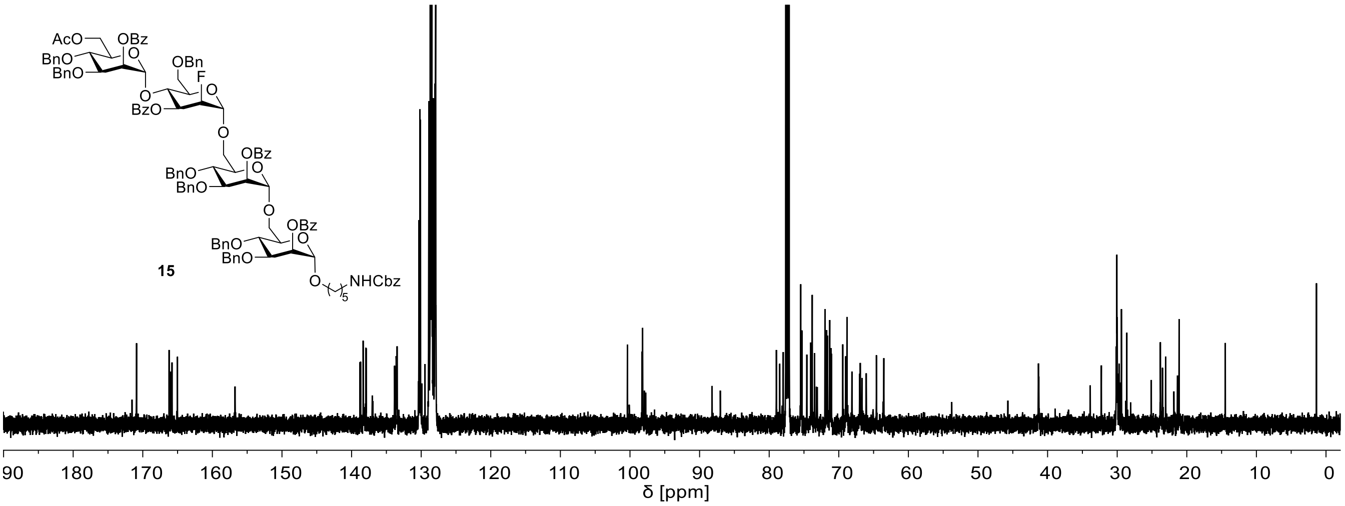

^{19}F NMR spectrum (564 MHz, $CDCl_3$) of compound **15**

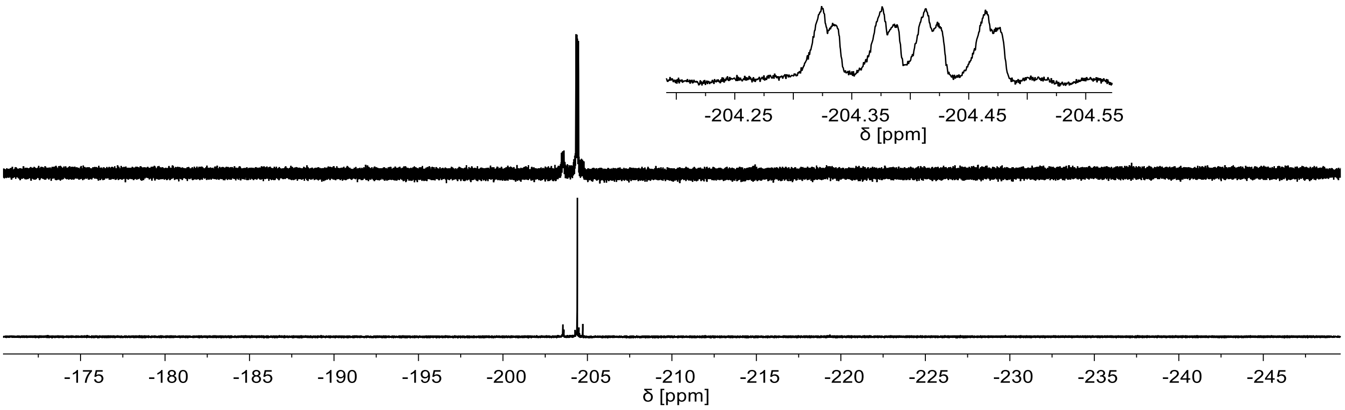

^1H NMR spectrum (599 MHz, $CDCl_3$) of compound **S14**

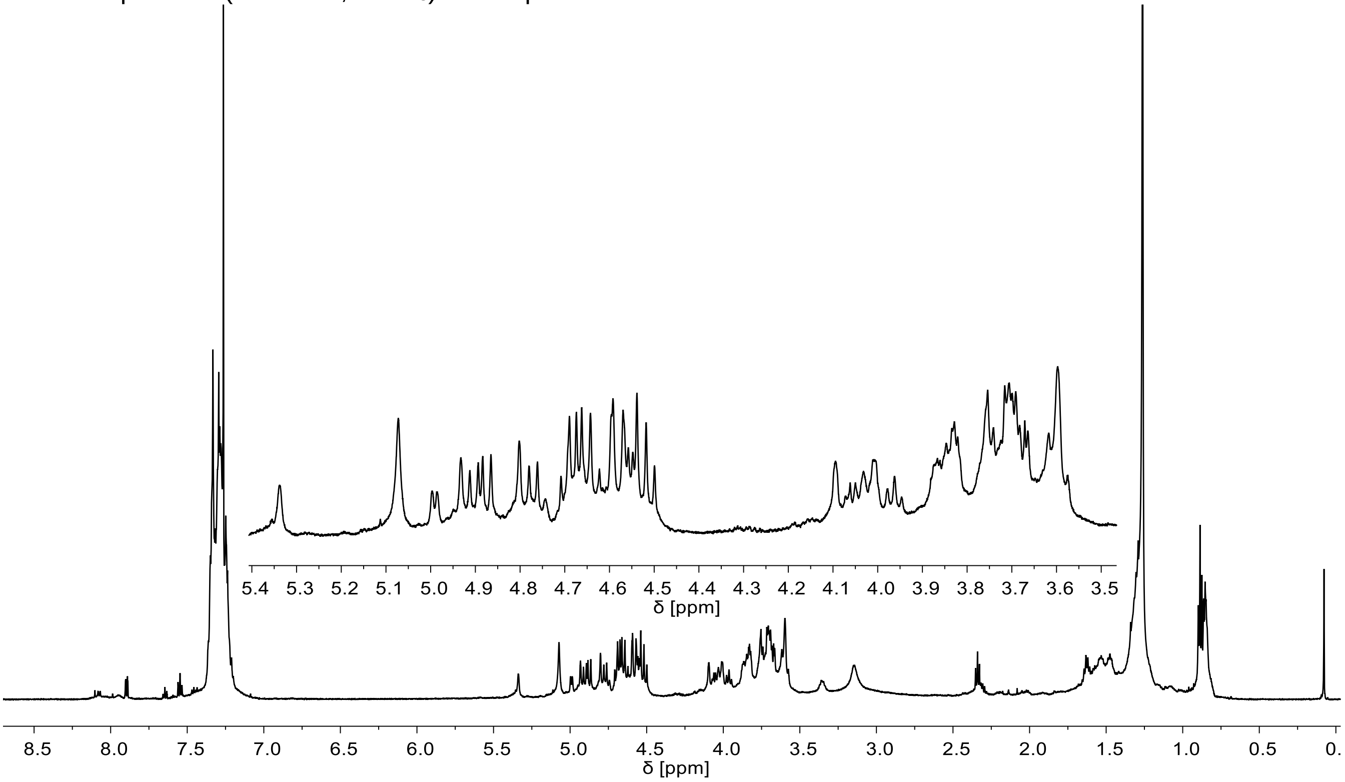

^{13}C NMR spectrum (151 MHz, $CDCl_3$) of compound **S14**

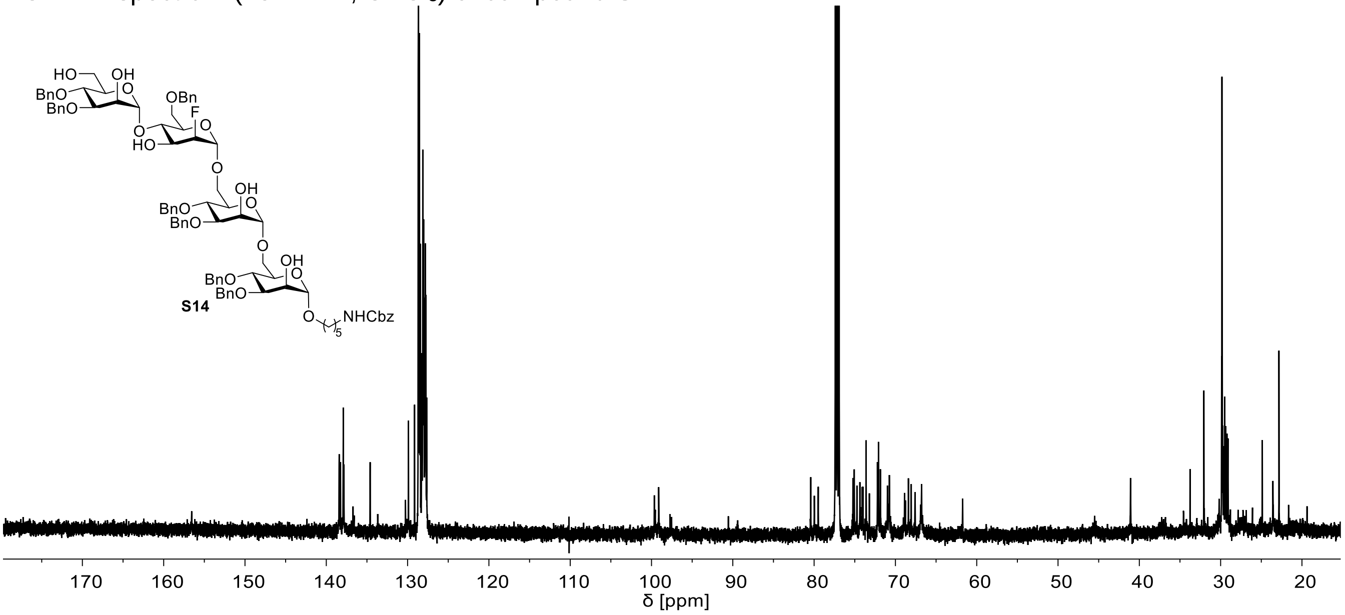

^{19}F NMR spectrum (564 MHz, $CDCl_3$) of compound **S14**

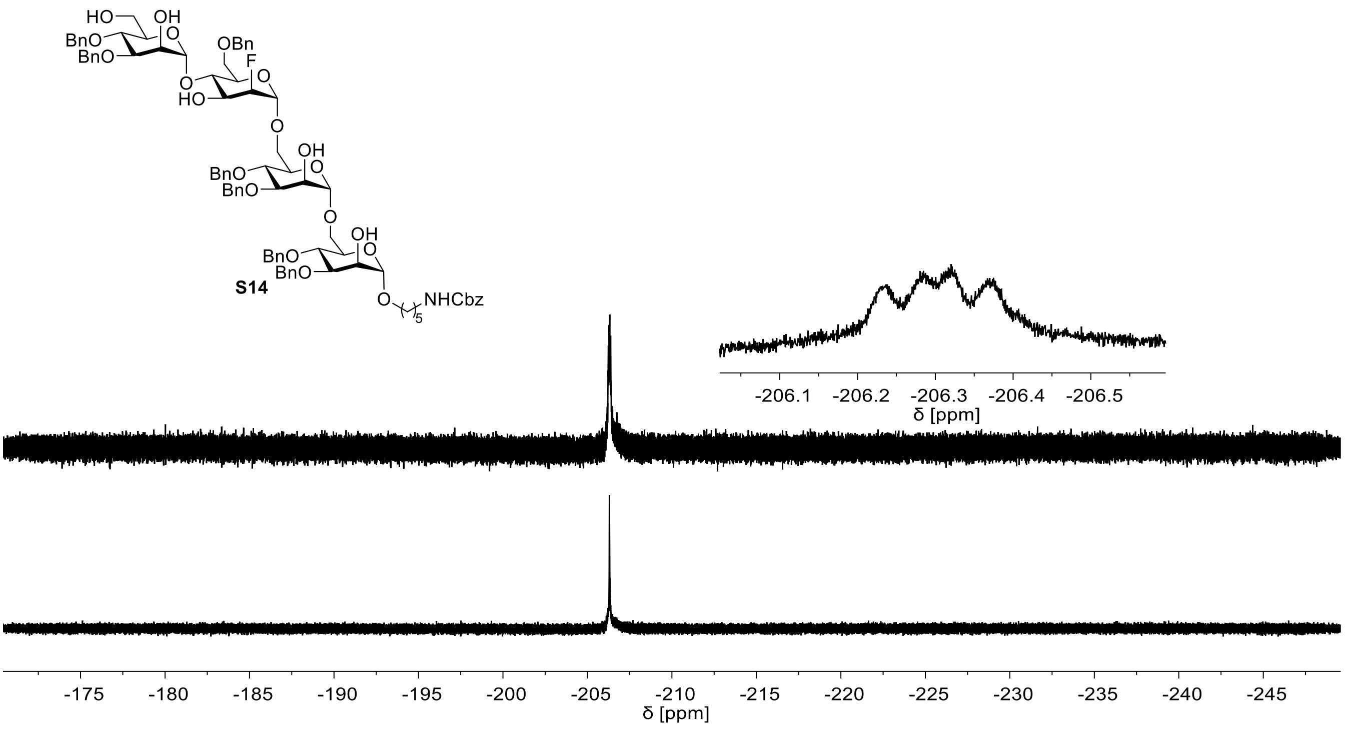

Crude $^{19}F\{^{1}H\}$ NMR spectrum (376 MHz, D_2O) of compound **S15**

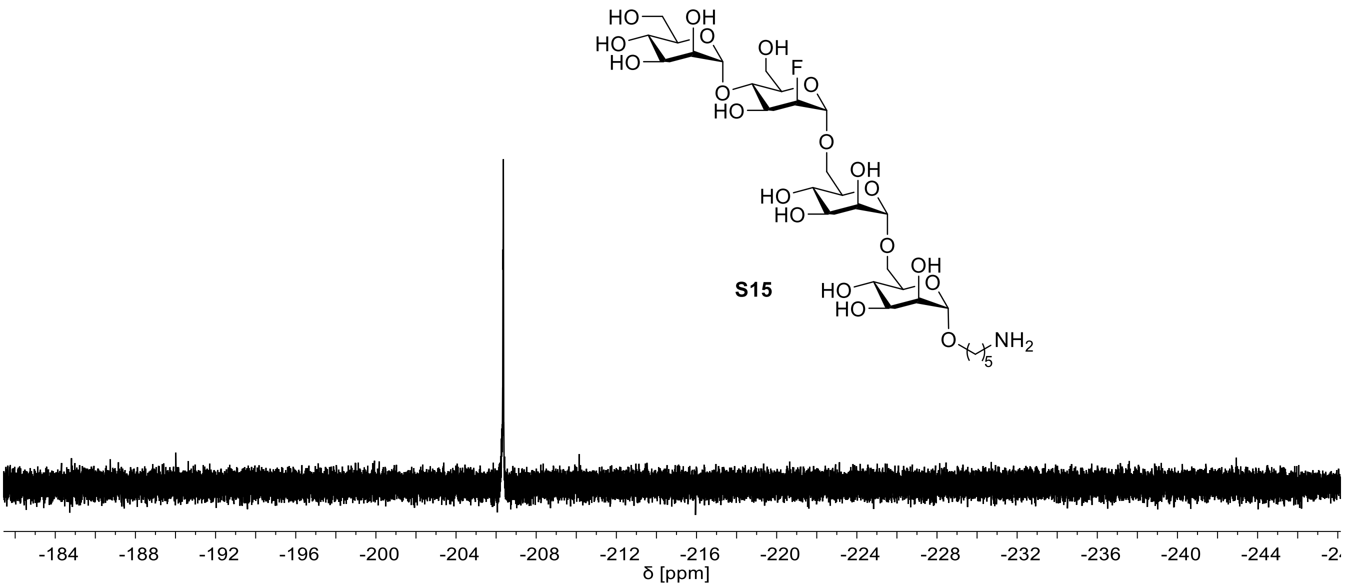

^{1}H NMR spectrum (599 MHz, $CDCl_3$) of compound **16**

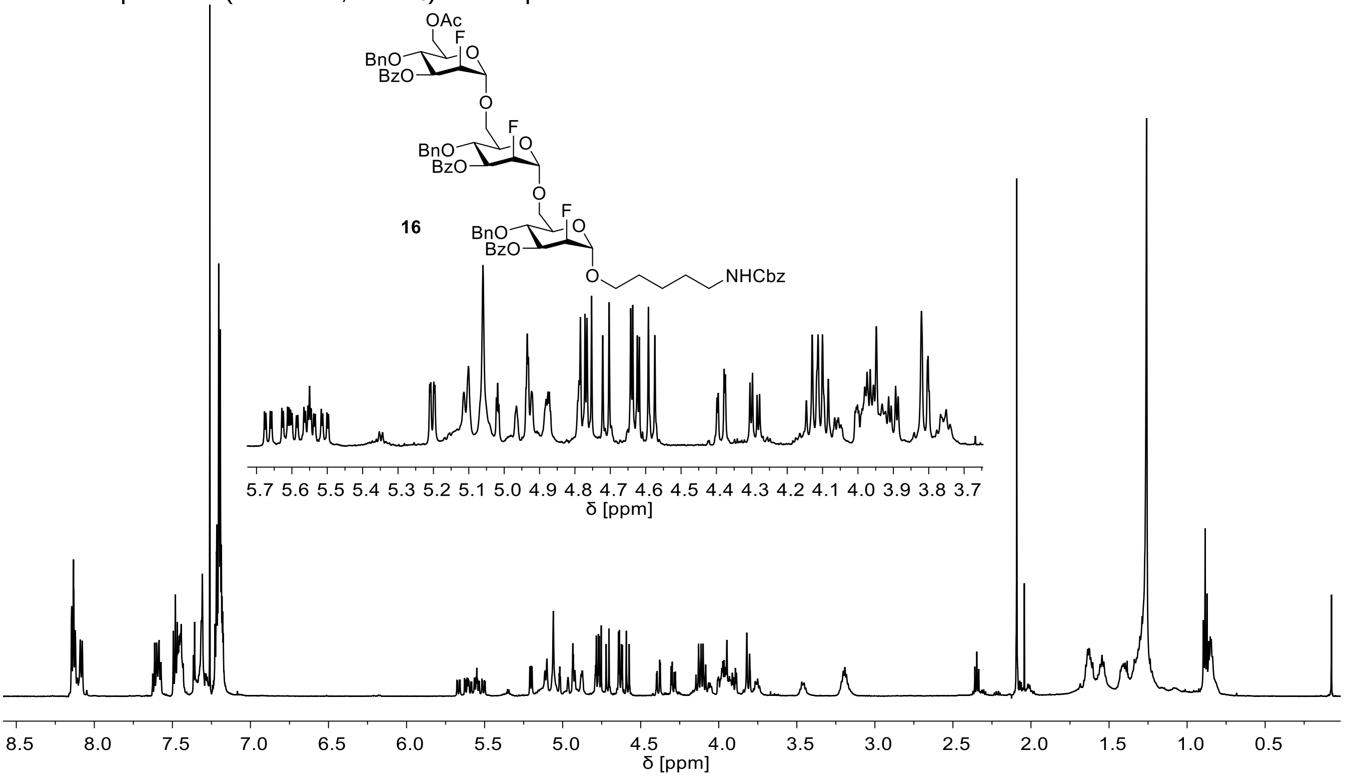

^{13}C NMR spectrum (151 MHz, $CDCl_3$) of compound **16**

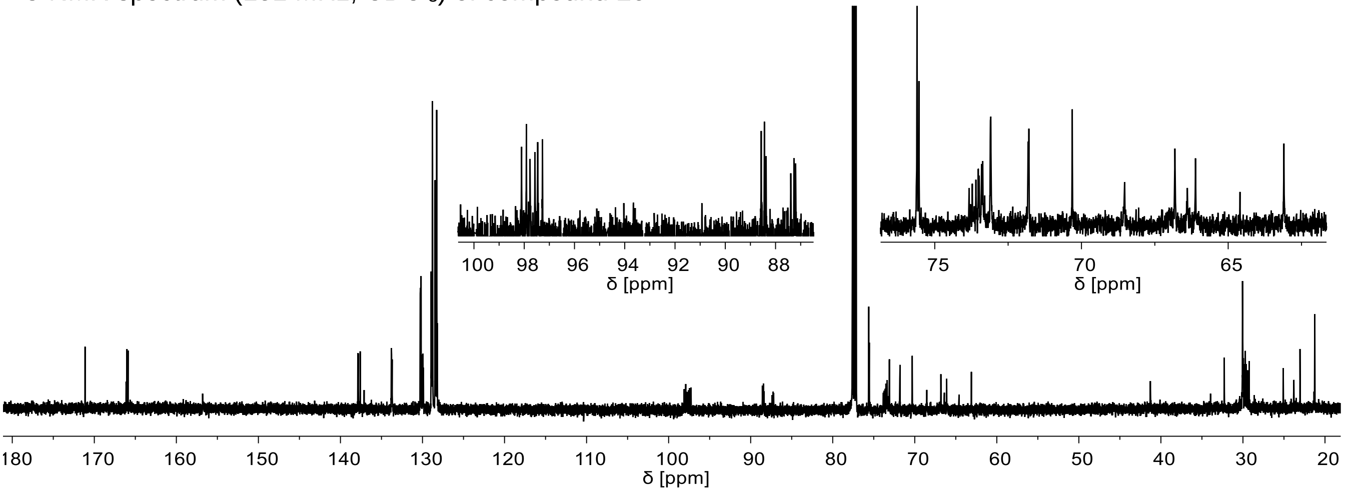

^{19}F NMR spectrum (564 MHz, $CDCl_3$) of compound **16**

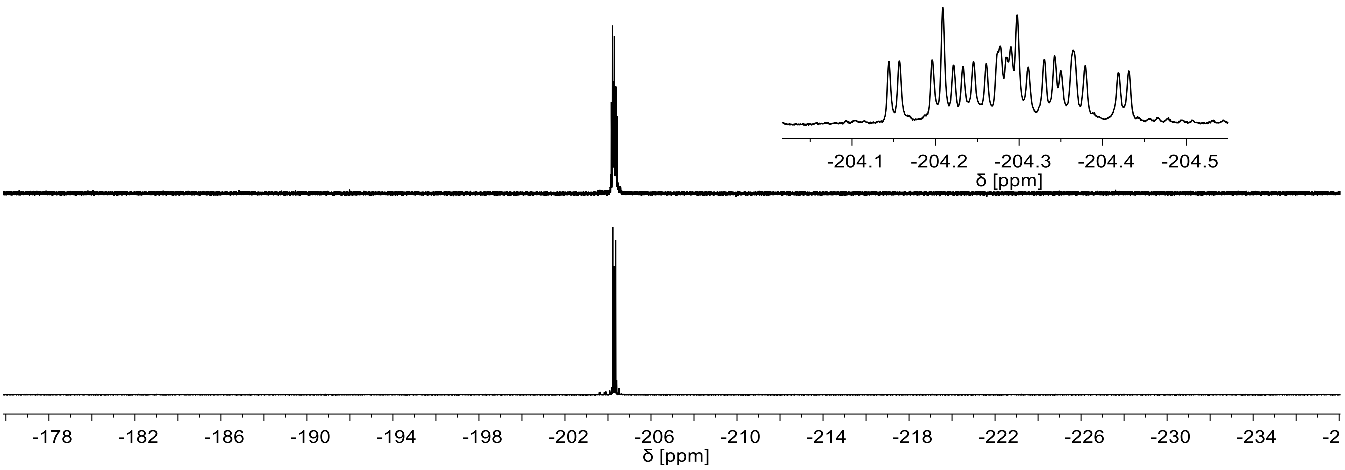

^{1}H NMR spectrum (599 MHz, $CDCl_3$) of compound **S16**

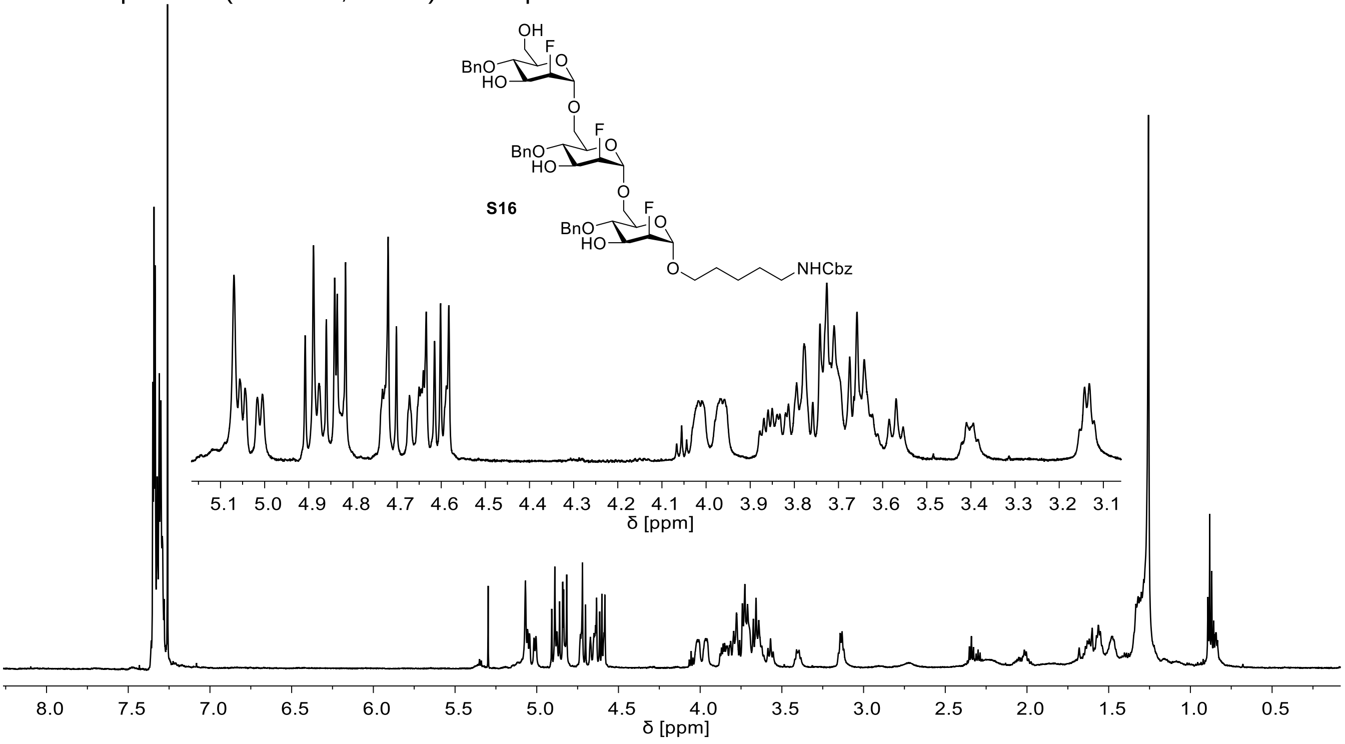

^{13}C NMR spectrum (5151 MHz, $CDCl_3$) of compound **S16**

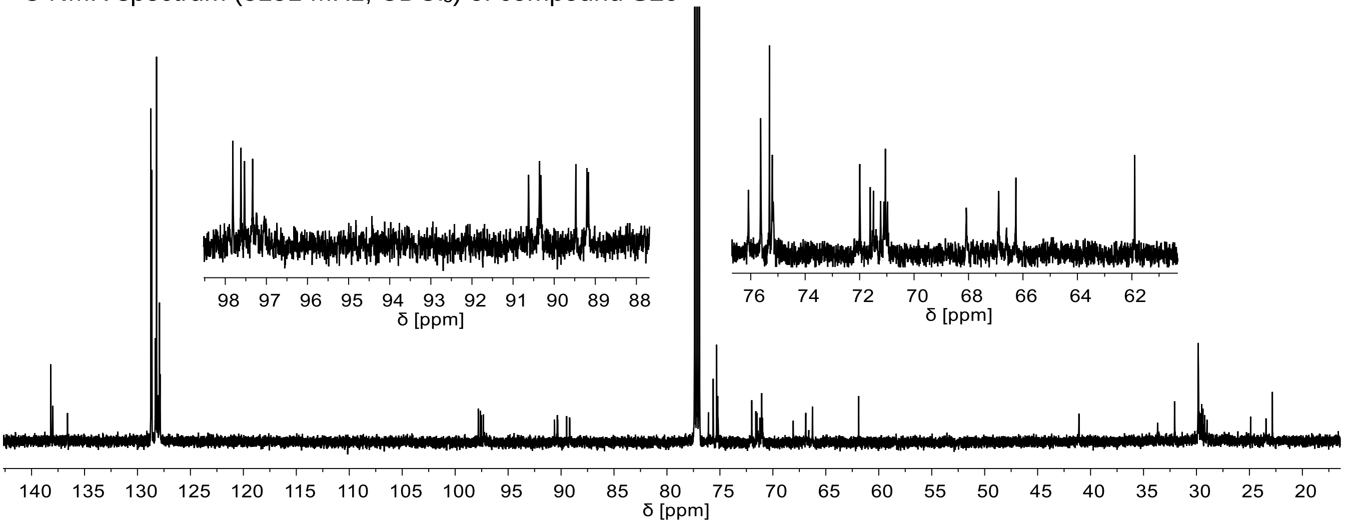

^{19}F NMR spectrum (564 MHz, $CDCl_3$) of compound **S16**

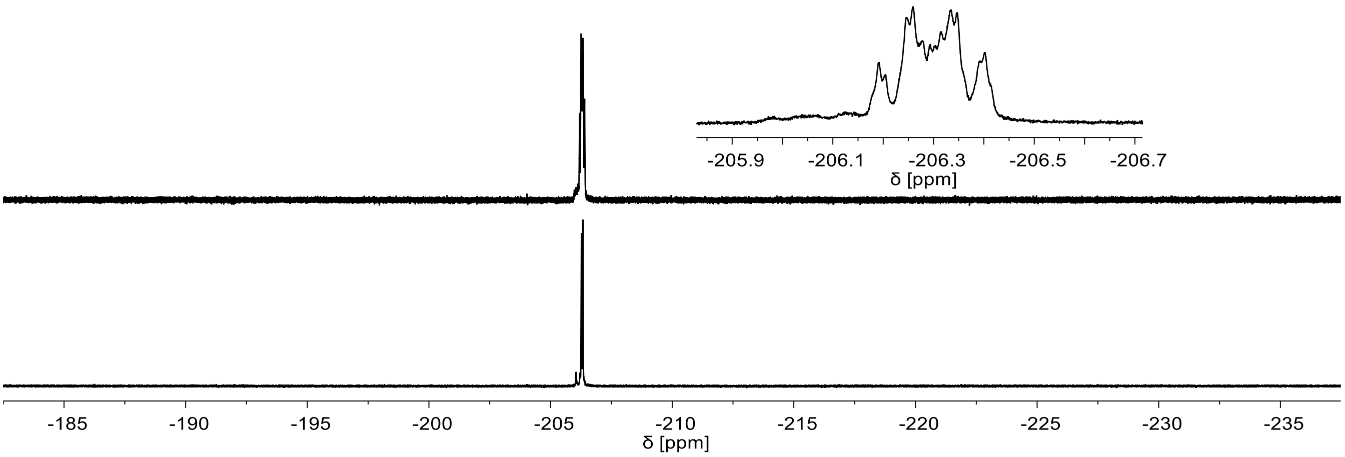

Crude $^{19}F\{^1H\}$ NMR spectrum (376 MHz, CD_3OD) of compound **S17**

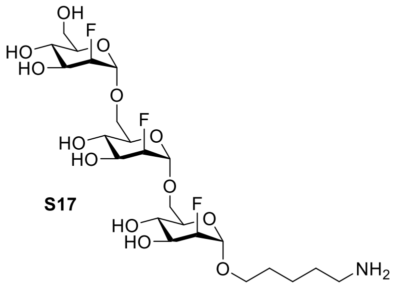

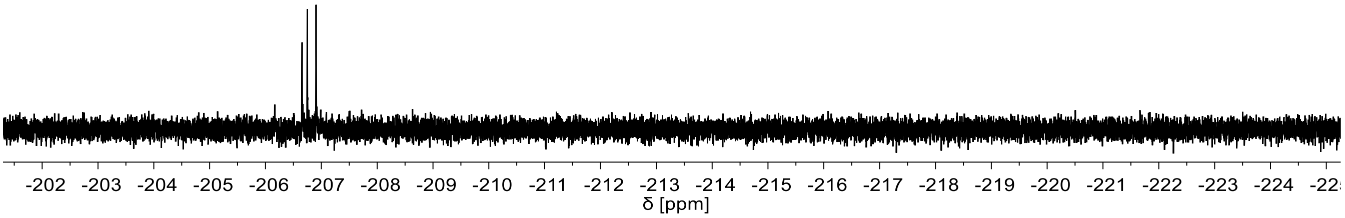

Crude $^{19}F\{^1H\}$ NMR spectrum (376 MHz, $CDCl_3$) of compound **20**

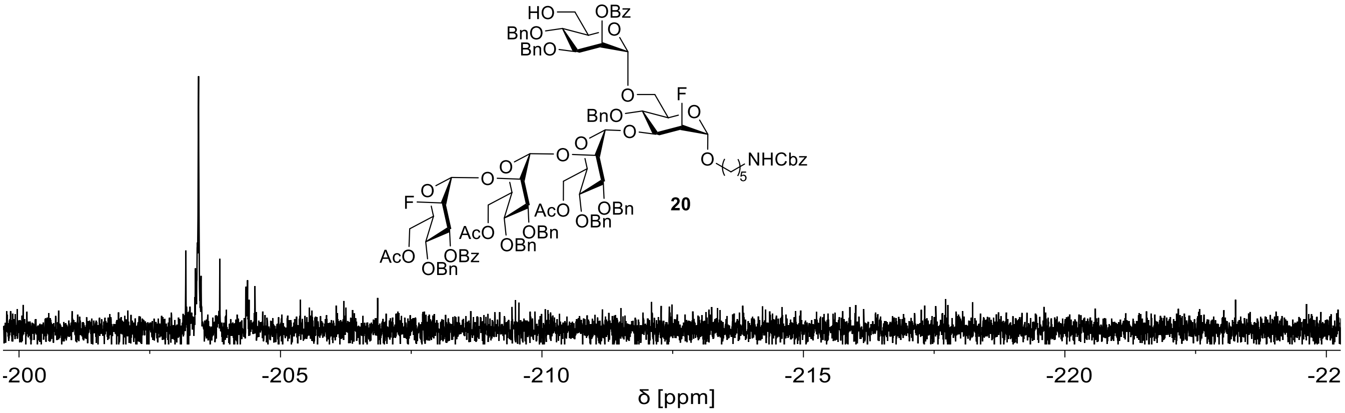

Supplement: Supplementary file 1 — Supporting Information [file ANIE-62-0-s001.pdf]
